# The roles of chromatin remodeling and 3D genome organization in cancers: from mechanistic insights to emerging treatment options

**DOI:** 10.1186/s12943-026-02763-x

**Published:** 2026-08-07

**Authors:** Chuqiao Luo, Ninglin Xia, Shin Yee Hui, U Sin Wong, Julia Y. Tsang, Hong Hu, Chi Man Tsang, Kwok Wai Lo, Peng Sun, Mumin Shao, Suresh K. Alahari, Wei Kang, Gary M. Tse, Dajiang Guo

**Affiliations:** 1 Department of Anatomical and Cellular Pathology and State Key Laboratory of Translational Oncology, Prince of Wales Hospital, The Chinese University of Hong Kong, Hong Kong, China; 2 Li Ka Shing Institute of Health Sciences, Faculty of Medicine, The Chinese University of Hong Kong, Hong Kong, China; 3 Department of Surgery, Shenzhen People’s Hospital (The Second Clinical Medical College, Jinan University, The First Affiliated Hospital, Southern University of Science and Technology), Shenzhen, Guangdong China; 4 State Key Laboratory of Oncology in South China; Collaborative Innovation Center for Cancer Medicine, Guangzhou, Guangdong China; 5 Department of Pathology, Sun Yat-Sen University Cancer Center, Guangzhou, Guangdong China; 6 Department of Pathology, Shenzhen Traditional Chinese Medicine Hospital, The Fourth Clinical Medical College of Guangzhou University of Chinese Medicine, Shenzhen, Guangdong China; 7 Department of Biochemistry and Molecular Biology, Louisiana State University Health Sciences Center at New Orleans, New Orleans, LA 70112 USA; 8 LSU-LCMC Cancer Center, Louisiana State University Health Sciences Center at New Orleans, New Orleans, LA 70112 USA

**Keywords:** Chromatin Remodeling, SWI/SNF Complex, Cohesin Complex, ARID1A, Chromosomal Architecture

## Abstract

Chromatin remodeling comprises a set of molecular mechanisms that regulate gene transcription, DNA replication, and DNA repair by altering nucleosome structure. Previous studies have found that chromatin remodelers are heavily mutated in cancer patients, and targeting aberrant chromatin remodeling activities holds great potential for clinical benefit. Despite these promising findings, several significant hurdles remain before this strategy can be successfully transitioned from bench to bedside. The classic concept of chromatin remodeling focuses on the linear 2D chromatin structural level. While the emergence of sophisticated sequencing modalities has underscored the significance of 3D chromatin architecture, the mechanistic underpinnings and broader implications for cancer biology continue to be largely elusive. This review provides a comprehensive synthesis of the mechanisms and functional roles of classical chromatin remodelers within both physiological and neoplastic contexts. Furthermore, we integrate emerging insights regarding the cohesin complex as a primary mediator of three-dimensional (3D) genomic organization. By proposing a hierarchical framework that distinguishes between 'first-level' (classical) and 'higher-order' chromatin remodeling, we aim to provide a more holistic understanding of the integrated regulatory networks governing chromatin architecture. Furthermore, we have systematically cataloged the landscape of therapeutic strategies targeting chromatin remodelers in oncology. By evaluating the divergence between clinically approved therapies and those currently in developmental pipelines, we delineate the primary challenges confronting the field and propose strategic directions for future research. Collectively, we have delineated the multifaceted contributions of chromatin remodeling to cancer progression. The strategic modulation of these remodeling processes represents a vital frontier in the development of novel therapeutic interventions and is likely to emerge as a primary focus for future cancer management strategies.

## Introduction to chromatin remodeling

Chromatin remodeling refers to the dynamic alternation of chromatin structure and accessibility driven by associated factors, which in turn regulate gene expression. As early as the 1970 s, researchers observed that changes in nucleosome arrangement could lead to altered transcriptional states [1]. These changes were identified not as a simple loss of nucleosomes, but as chromatin structural rearrangements along the linear DNA sequence, while the core histones remained in place [2].

**Fig. 1:**
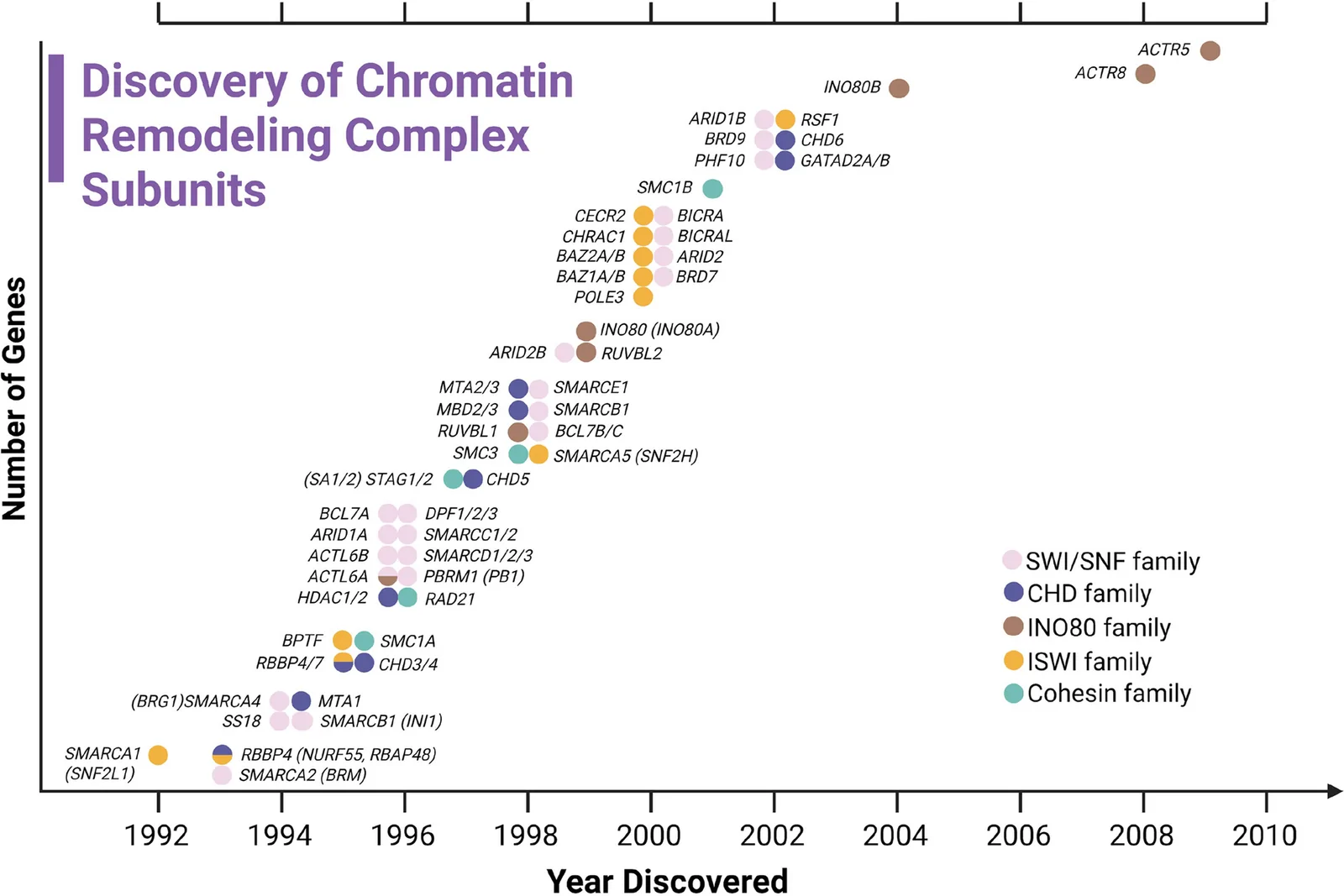
Discovery timeline of Chromatin Remodeling complex Subunits. Timeline of the discovery of key subunits of chromatin remodelers. SWI/SNF: switch/sucrose nonfermentable. ISWI: Imitation Switch. CHD: chromodomain-helicase DNA-binding. INO: Inositol-requiring mutant 80. The names of the subunits are mostly limited to mammals; several common alternative names from other model organisms were included

The chromatin remodeling field has been established and advanced through the genetic identification of key protein complexes (Fig. 1). In 1984, the first chromatin remodeling factor was identified via yeast genetics as the SWI complex, whose main subunit was later confirmed to be ATP-dependent [3, 4]. Subsequent discoveries included the Drosophila Brahma (BRM) protein, a component of the switch/sucrose nonfermentable (SWI/SNF)-like complex [5, 6], and the SNF2 family member hSNF2L (SMARCA1) in human cell lines [7]. Extensive work further delineated the components of these remodeling complexes, uncovering defined families such as the Drosophila Imitation Switch (ISWI), a homolog of hSNF2L, and the mammalian hSNF2H (SMARCA5) [8, 9]. These functionally related complexes were later categorized into four major families: SWI/SNF, ISWI, inositol-requiring mutant 80 (INO80), and chromodomain-helicase DNA-binding protein (CHD). By the mid-1990s, the term “chromatin remodeling” was formally adopted to describe this ATP-driven reconfiguration of chromatin [10].

Interestingly, chromatin structure is not simply linear. The discovery of distal regulatory elements supported a DNA looping model [11, 12] (Fig. 1). Specifically, DNA can form loops in a three-dimensional (3D) manner, facilitating the long-distance contacts of upstream and downstream genes, as proposed in early 1984 [13, 14]. Genomic technologies have since revolutionized our understanding of this 3D architecture. In 2002, the Capturing Chromosome Conformation (3C) technology was developed to decipher the spatial organization of DNA using quantitative PCR [15]. This was followed by high-throughput adaptations: circular capturing chromosome conformation (4C) was designed to probe all interactions within a single locus of interest, while chromosome conformation capture carbon copy (5C) enables parallel investigations of multiple specific loci [16, 17]. The advent of Hi-C technology subsequently allowed the identification of genome-wide chromosomal interactions and led to the development of numerous derivative methods, such as Hi-ChIP, Micro-C, and Capture C [18–20]. Notably, Hi-C studies identified cohesin, together with the CCCTC-binding factor (CTCF) protein, as a pivotal complex that mediates enhancer-promoter looping and organizes the 3D genome [18].

Modern sequencing technologies have been instrumental in confirming genomic structure at multiple scales, thereby expanding the classical concept of “chromatin remodeling”. It is now well understood that chromatin is distributed within the nucleus sparsely yet intentionally. This 3D territory is functionally linked to the transcriptional accessibility and broadly partitioned into open (euchromatin) and closed (heterochromatin) compartments. The discovery of the topologically associating domain (TAD) in 2012, enabled by Hi-C technology, led to the proposal of the loop extrusion model [21–24]. This model was directly validated in 2019 through molecular experiments that visualized the DNA loop extrusion process [25]. Recent studies have revealed a new layer of chromatin organization beyond the linear, ATP-driven nucleosomes repositioning carried out by the “first-level” remodeler. This “higher-order chromatin remodeling” is primarily mediated by cohesin-related architectural changes that also critically influence chromatin accessibility.

It is now well-established that alterations in chromatin remodeling factors are critically involved in the progression and therapeutic response of numerous cancers. Furthermore, extensive correlations exist between the first-level and higher-order chromatin remodeling. Despite these advances, the precise molecular mechanisms that govern this interplay remain unclear. Therefore, this review aims to elucidate the key factors and mechanisms underlying both first-level and higher-order chromatin remodeling, synthesize the functional relationships between them, and discuss their collective impact on cancer pathogenesis.

## Types of chromatin remodeling complexes

### First-level chromatin remodeling complexes

**Fig. 2:**
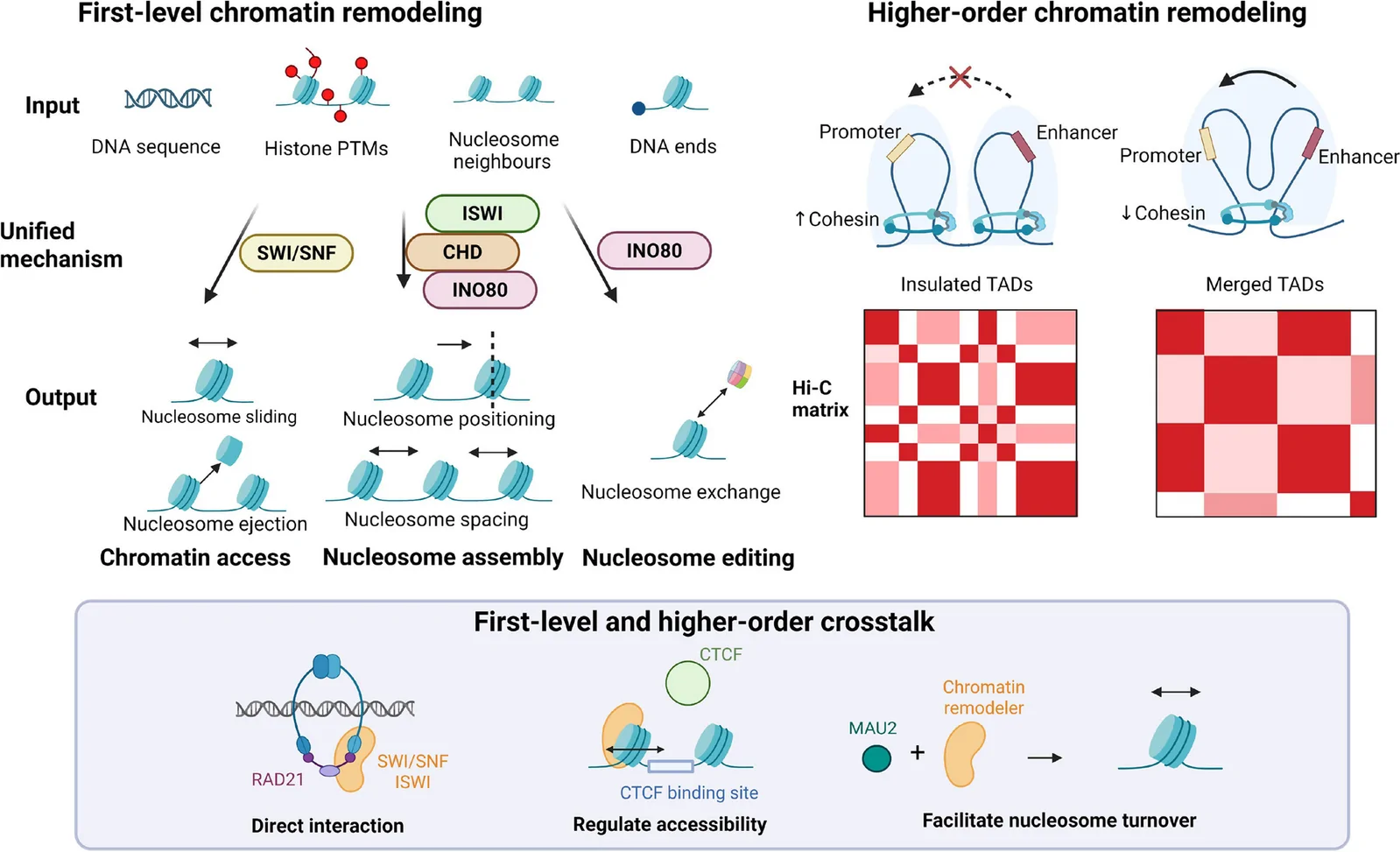
Crosstalk between the first level and higher-order chromatin remodelers. Schematic models of first-level and higher-order chromatin remodeling. In the hourglass model of first-level chromatin remodeling, chromatin remodelers recognize diverse input signals and, through a unified mechanism of action, achieve various outcomes in nucleosome turnover. SWI/SNF complexes primarily mediate nucleosome sliding and ejection. ISWI and CHD complexes are mainly involved in nucleosome assembly and spacing. INO80 additionally facilitates nucleosome exchange. Higher-order chromatin remodeling regulates enhancer–promoter interactions by modulating topologically associating domains (TADs). Increased cohesin levels lead to more TADs, segregating enhancers and promoters into distinct TADs and resulting in transcriptional repression. Conversely, reduced cohesin brings enhancers and promoters into closer proximity, leading to transcriptional activation. Crosstalk exists between first-level and higher-order chromatin remodeling. For example, the core cohesin subunit RAD21 or the regulatory subunit MAU can directly interact with specific chromatin-remodeling subunits. Additionally, chromatin remodelers can regulate three-dimensional genome architecture by modulating nucleosomes at CTCF-binding sites

DNA wraps around histone proteins to form nucleosomes, which sterically hinder transcription factors from accessing the DNA template. First-level chromatin remodelers, often working in concert with transcription factors, alter nucleosome positioning to regulate gene access (Fig. 2). These ATP-dependent complexes are defined by a core SWI2/SNF2-family subunit containing DExx and HELICc ATP-binding domains. They can be further categorized into four major families based on their distinct core subunits and biochemical activities [26].

#### SWI/SNF family

The SWI/SNF family, also known as the BRG1/BRM-associated factor (BAF), is the first identified and among the most crucial chromatin remodelers [3, 27, 28]. Mammalian SWI/SNF complexes comprise three subfamilies, which are canonical BAF (cBAF), polybromo-associated BAF (pBAF), and the recently discovered non-canonical BAF (ncBAF) [29–31]. All share a similar catalytic subunit, with its ATP-binding domains located between the Helicase-SANT-associated (HSA) and a bromodomain [26]. Common subunits of all three subfamilies include SMARCC1/2, SMARB1, SMARE1, and the conserved ATPase subunits SMARCA2/4. Subfamily identity is defined by unique components, such as mutually exclusive ARID1A or ARID1B in cBAF, ARID2 and BRD7 in PBAF [32–35], and BRD9 and GLTSCR1/1L subunits characterized in ncBAF [4, 36, 37]. Powered by ATP, the SWI/SNF complex binds to the nucleosome and translocates DNA, resulting in nucleosome sliding or ejection [38]. Interestingly, the subfamilies exhibit distinct genomic targeting. For instance, cBAF is highly enriched at enhancers, while pBAF and ncBAF primarily bind to promoters and, less frequently, to enhancers [27, 37, 39]. SWI/SNF complexes are essential in nucleosome positioning, facilitating transcription factor interactions, and mediating DNA damage repair [40–43]. Many SWI/SNF subunits, such as ARID1A/B, are frequently mutated across a wide variety of cancers, indicating a tumor-suppressive function. However, these complexes can also remodel chromatin accessibility to promote tumor progression (under normal circumstances) [44, 45]. As a result, the role of SWI/SNF in cancer is highly context-dependent.

#### ISWI family

The core subunit of the ISWI complex contains a conserved ATPase domain and a characteristic HAND-SANT-SLIDE domain that binds to the unmodified histone H4 tail and linker DNA [46, 47]. Unlike the SWI/SNF, ISWI complexes possess an autoinhibition mechanism regulated by the AutoN and NegC domains, ensuring that remodeling activities are directed to the appropriate chromatin landscape [48]. ISWI ATPase-containing NURF, CHRAC, and ACF complexes were first identified in *Drosophila* and are conserved in species including yeast and mammals [46, 49–52]. Subsequent research in mammals has identified additional family members, such as hWICH and hNORC, expanding the mammalian ISWI family to 16 distinct complexes [53, 54]. Mammalian ISWI complexes are typically formed by one catalytic subunit, either SMARCA5 (hSNF2H) or SMARCA1 (hSNF2L), and one to three regulatory subunits. In general, CHRAC and ACF regulate nucleosome spacing to optimize nucleosome assembly and repress transcription, while NURF randomizes nucleosome spacing at promoters in concert with transcription factors [55]. Beyond transcriptional regulation, ISWI complexes are critical for processes including DNA repair and the modulation of insulator region accessibility [56]. In cancers, ISWI-containing complexes often exhibit tumor-promoting activity by supporting oncogenic programs. However, aberrant expression of ISWI subunits can itself drive oncogenesis [46].

#### CHD family

The CHD family consists of nine members, termed from CHD1 to CHD9. These proteins share common structural motifs, which include an SNF2-like ATPase domain and a chromatin organizing (CHROMO) domain, facilitating interactions with methylated histone [57]. Based on different domain characteristics, the CHD family is further divided into three subfamilies. Subfamily I, comprising CHD1 and CHD2, is characterized by a SANT-SLIDE DNA-binding domain that recognizes AT-rich DNA motifs [58]. Subfamily II (CHD3, CHD4, and CHD5) is distinguished by tandem plant homeodomain (PHD), which binds to unmodified or K9-methylated histone H3 tails [59]. Subfamily III, which includes CHD6 through CHD9, shares a common set of features, including Brahma and Kismet (BRK) domain, SANT-SLIDE domain, and other conserved regions [60, 61]. Functionally, while certain CHD enzymes slide nucleosomes to promote transcription, members of the CHD subfamily II primarily promote gene silencing as core components of the nucleosome remodeling and deacetylase (NuRD) complex [62]. Notably, CHD subfamily II members are well-established tumor suppressors, with CHD5 being the first family member shown to suppress tumor progression by regulating proliferation, apoptosis, and senescence [63]. Nevertheless, the roles of CHD subfamily I and III in cancer are also context-dependent, with individual members exhibiting either oncogenic or tumor-suppressive functions.

#### INO80 family

The INO80 ATPase is characterized by a long insertion between its DExx and HELICc domains, as well as regulatory domains such as HSA. Structurally related, SWR1 is typically classified within the INO80 family and is often discussed alongside INO80 [64, 65]. The human orthologs of the yeast INO80 and SWR1 include hINO80, hSRCAP, and p400, respectively. Both the INO80 and SWR1 complexes share RuvB-like proteins and actin-related proteins (Arp), but they assemble into different topologies [66]. Their mechanisms of nucleosome engagement also differ from those of other chromatin remodelers, while the SWR1 complex clings to the nucleosome, and the INO80 complex grasps the nucleosome “like a hand” [67, 68]. Although both the INO80 and SWR1 complexes promote transcription and participate in DNA repair, they act through distinct biochemical activities. Specifically, the INO80 complex performs functions such as nucleosome sliding and eviction, while SWR1 was initially implicated in Htz1 recruitment and subsequently established as the dedicated H2A.Z deposition machinery that replaces H2A: H2B dimers with H2A.Z: H2B dimers [69–73]. Elevated levels of INO80 have been implicated in both the initiation and progression of cancer [74, 75].

### Higher-order chromatin remodeling complexes

Beyond modulating DNA accessibility via first-level chromatin remodelers, the 3D architecture of chromatin profoundly influences gene transcription by forming DNA loops that facilitate enhancer-promoter communication (Fig. 2). The key driver of this higher-order chromatin remodeling is the cohesin complex, whose activity is spatially restricted by the CTCF [76, 77].

#### Cohesin complex

The cohesin complex consists of four core subunits: SMC1A, SMC3, STAG1/2, and RAD21. SMC1A and SMC3 form a V-shaped heterodimer through interacting at their hinge domains. RAD21 bridges the ATPase domains of SMC1A and SMC3, altogether forming a ring-like structure. STAG1/2 binds to the central region of RAD21, playing an essential role in chromatin loading. Cohesin function is regulated by diverse cofactors that determine its activity under different circumstances. During the G1 phase, the Nipped-B-like protein (NIPBL) and MAU2 chromatid cohesion factor homolog (MAU2) promote the loading of the cohesin complex onto the chromatin. Conversely, the wings apart-like protein homolog (WAPL) and sister chromatid cohesion protein PDS5 homolog A/B (PDS5A/B) act as unloaders [78]. In sister chromatid cohesion, sororin interacts with the cohesin complex during S phase, while separase removes cohesin from centromeres in M phase by cleaving RAD21 [79]. Although the cohesin complex is involved in diverse biological processes, this review focuses primarily on its cohesion-independent functions, namely, genome compartmentalization, transcription regulation, and DNA replication, all mediated through DNA looping. In conjunction with CTCF, cohesin not only promotes long-range DNA contacts but also helps establish insulator boundaries, thereby reshaping enhancer-promoter interactions and regulating gene transcription [80]. Cohesin has conventionally been viewed as a tumor suppressor due to the prevalence of loss-of-function (LOF) mutations in its subunits across several cancers [81]. Exceptionally, the core subunit RAD21 is frequently amplified in other cancer types, revealing a complex and context-dependent relationship between cohesin-mediated chromatin remodeling and tumorigenesis [82].

## Molecular mechanisms of chromatin remodeling

### ATP hydrolysis-driven DNA translocation on nucleosomes

The defining feature of first-level chromatin remodelers is a core Snf2-like subunit containing two conserved RecA-like lobes: an N-lobe with an ATPase domain and a C-lobe with a helicase domain [83]. In the absence of DNA binding, this Snf2-like ATPase is allosterically inhibited in the apo state. Upon ATP consumption, its ATPase undergoes a conformational change, generating a one-base-pair bulge at the SHL2 of the nucleosome. In the subsequent ADP-bound state, Snf2-like ATPase distorts the DNA tracking strand, pulling in DNA from the proximal end. The binding of a new ATP molecule causes the DNA guide strand to follow the tracking strand, effectively rotating the DNA forward by one base pair [84, 85]. Through this stepwise translocation, the motor ATPase remodels DNA-histone contacts and progressively moves DNA along the histone surface [86]. Furthermore, the accumulation of DNA twist defects within the nucleosome facilitates nucleosome sliding [84, 85, 87].

Despite their diversity, ATP-dependent chromatin remodelers share a core principle. As articulated in Cedric R Clapier’s “hourglass” model, they employ a unified mechanism of histone-anchored DNA translocation to produce various nucleosome outcomes [86] (Fig. 2). In this model, inputs include genetic features (e.g., DNA sequence) and epigenetic features (e.g., histone post-translational modifications (PTMs)). Meanwhile, the outcome comprises distinct structural changes such as nucleosome sliding, positioning, spacing, ejection, and histone exchange [88]. The functional versatility of remodelers arises from their specific structural domains and sensitivity to regulatory signals. For example, the efficiency of DNA translocation regulated by ARPs determines whether the SWI/SNF complex facilitates nucleosome sliding or ejection [89]. Similarly, a high-throughput nucleosome remodeling assay revealed that the binding preferences of ISWI non-ATPase subunits exert both stimulatory and inhibitory effects on diverse nucleosome modifications [90].

Ultimately, chromatin accessibility is predominantly governed by nucleosome organization and occupancy. A single nucleosome occupies approximately 146 bp of DNA, physically blocking transcription factors from binding. Through nucleosome sliding or ejection, remodelers can expose previously occluded DNA, making it accessible to transcription factors. Correspondingly, promoters and enhancers exhibit higher nucleosome turnover and lower occupancy than transcriptionally inactive regions [91]. Consequently, by dynamically regulating nucleosome positioning, chromatin remodeling directly controls transcription by modulating gene accessibility.

### Role of the cohesin complex in 3D structure

**Fig. 3:**
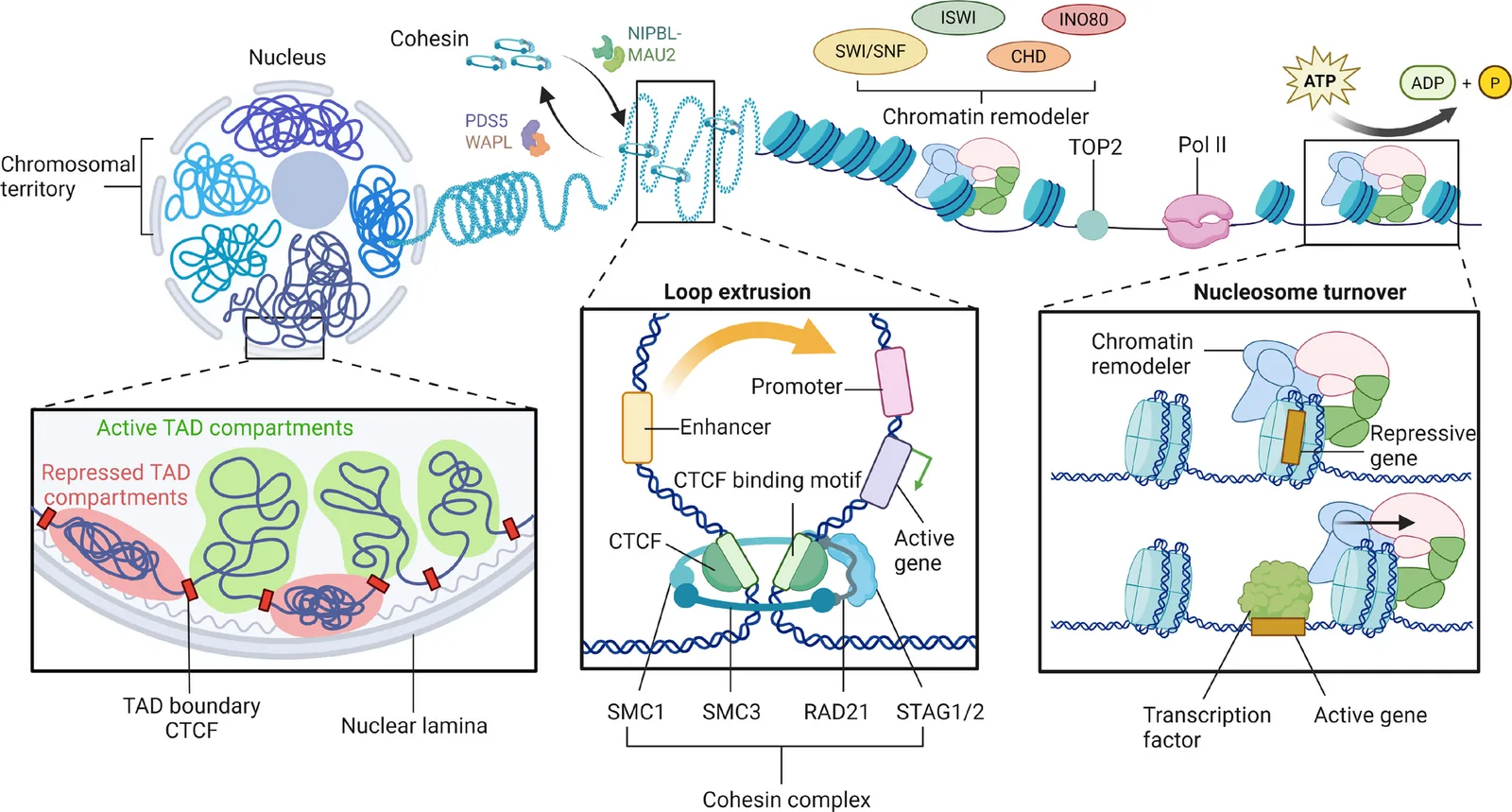
Machinery of chromatin remodeling. Schematic representation of chromatin spatial organization within the nucleus and its regulation by cohesin and chromatin remodelers. The nucleus is partitioned into multiple chromosomal territories, which include transcriptionally active and repressive topologically associating domains (TADs). This spatial chromatin architecture is potentially regulated by cohesin. Cohesin is a ring-shaped complex composed of SMC1, SMC3, RAD21, and STAG1/2. Under the control of the NIPBL–MAU2 complex, cohesin is loaded onto DNA and slides along the DNA strand, forming DNA loops that bring enhancers and promoters into proximity to regulate gene expression—a process known as loop extrusion. Upon encountering convergently oriented CTCF binding sites, cohesin slows markedly and is eventually dissociated by PDS5 and WAPL. In parallel, chromatin remodelers regulate gene expression through nucleosome remodeling. Typically, upon binding to nucleosomes, chromatin remodelers consume ATP to twist and displace the DNA wrapped around histone cores, resulting in nucleosome sliding. This process exposes previously occluded genomic sequences to the linker DNA, thereby increasing DNA accessibility. Consequently, DNA topoisomerase 2 (TOP2) and RNA polymerase II (Pol II) can access and function on the DNA, facilitating transcription

After modulating DNA accessibility by the first-level chromatin remodelers, the cohesin complexes further orchestrate higher-order chromatin architecture by forming DNA loops. As shown in Fig. 3, ATP hydrolysis-powered cohesin complexes rapidly interact with DNA to form symmetrical loops, employing a pseudo-topological or non-topological mechanism [25]. This process, termed loop extrusion, involves cohesin complexes that diffuse along DNA; in vivo, they have been observed to stop specifically at CTCF-anchored sites in convergent orientations [77, 92, 93]. Cohesin and CTCF also stall at the boundaries of TADs, which are highly conserved across species [21]. TADs are dynamic entities sustained by continuous cohesin-mediated loop extrusion rather than by static loops [94]. While cohesin and CTCF cooperate to maintain TAD structure, they play different roles. While CTCF serves as an insulator whose depletion reduces intra-TAD interactions and increases aberrant contacts between TADs, cohesin loss results in a global decline in chromosomal interactions but does not alter TAD partitioning [95]. Conversely, excessive cohesin activity or prolonged binding time can lead to hyper-compacted, vermicelli-like chromatin structures, bypassing the normal insulating barrier provided by CTCF [96–98].

Genomic folding occurs hierarchically, with DNA loops organized into TADs that further aggregate into larger compartments associated with transcription states. At a resolution of 1 megabase, Hi-C data revealed that the genome is partitioned into A (transcriptionally active, open) and B (repressive, closed) compartments based on eigenvector analysis [18]. Notably, upon depletion of the cohesin subunit RAD21, cohesin-mediated loops and TADs dissolve, giving way to compartmental domains that reduce local contacts while facilitating long-distance compartmental interactions [99]. In summary, cohesin is fundamental for establishing loops and TADs, while its activity generally antagonizes the formation of compartmental domain structures (Fig. 2).

### Collaboration between first-level and higher-order chromatin remodeling

Current studies suggest that classical remodelers may function at all three levels: upstream of cohesin loading, downstream of 3D genome topology, and in parallel pathways converging on transcription. The mechanism by which first-level chromatin remodeling affects higher-order organization is not fully resolved. However, it is hypothesized that first-level remodeling affects the formation and stability of 3D chromatin structures, while higher-order remodeling, in turn, regulates the assembly of first-level remodelers [88]. Recent findings reveal that first-level remodelers directly interact with cohesin and its cofactors. For example, ARID1A, the core subunit of the SWI/SNF complex, can affect DNA looping, TAD forming, and A/B compartment switching by influencing SMARCA4-RAD21 interaction [100]. Additionally, SNF2h (the ATPase of ISWI) binds RAD21 and facilitates cohesin loading onto chromatin, an association that can be regulated by DNA methylation [101]. Beyond their direct effect on the cohesin complex, certain chromatin remodelers can modulate the accessibility of the insulator protein CTCF by regulating nucleosome occupancy around CTCF-binding sites, thereby affecting CTCF binding and its interaction with cohesin [102–104]. Interestingly, the cohesin loader MAU2 contacts the ATPase domain of chromatin remodelers and directly stimulates nucleosome turnover at promoters [105]. Therefore, first-level and higher-order chromatin remodeling mutually influence each other and jointly regulate gene expression.

Liquid–liquid phase segregation (LLPS) is another widely accepted mechanism to elucidate spatial genome organization and function. Chromatin remodelers such as ISWI and SWI/SNF can regulate LLPS by repositioning nucleosomes, thereby potentially inhibiting heterochromatic silencing [106, 107]. A recent phenomenal model strengthens the concept of collaboration between first-level chromatin remodelers and cohesin. In this model, cohesin functions as a driver of nascent domain formation through loop extrusion, whereas first-level remodeler and other remodeling enzymes help transform these nascent domains into mature structures characterized by a condensed heterochromatic core and a lower-density surface [108]. The differential access, in which smaller heterochromatic enzymes can enter the high-density core and larger euchromatic enzymes are restricted to the periphery, results in RNA synthesis peaks at intermediate densities within the mature domain [108]. Despite the variety of proposed models, cohesin and first-level remodelers are functionally interlinked and collectively achieve the dynamic unification of chromatin structure and gene transcriptional function.

### Role in enhancer-promoter interaction and transcriptional regulation

Enhancer-promoter communication represents a key downstream of chromatin remodeling, Especially, in SWI/SNF-mutant cancers, it is increasingly clear that altered enhancer logic and DNA damage repair are intertwined. Cohesin-mediated remodeling enables long-range interactions between enhancers and promoters, while the insulator CTCF prevents abnormal enhancer-promoter pairing, thus controlling gene transcription [109]. Notably, some promoters are CTCF-dependent, and genes bound by promoter-proximal CTCF largely rely on long-distance enhancers [110]. In yeast, the remodeling structure of chromatin (RSC) complex binds promoter sequences and induces nucleosome eviction adjacent to the promoter, thereby maintaining promoter accessibility and preserving a nucleosome-free region for RNA polymerase II (RNAPII) binding [111, 112].

Super enhancers are clusters of highly active enhancers that interact with abundant transcription factors and Mediators, controlling the expression of genes that often promote cancer and define cell identity [113, 114]. SMARCB1, a core subunit of the SWI/SNF complex, is essential for enhancer binding but dispensable for super-enhancer occupancy. Loss of SMARCB1, detected in the majority of rhabdoid tumors, impairs regular enhancer function while leaving super enhancers active, and such an imbalance may drive oncogenesis by disrupting the cell differentiation programs normally mediated by typical enhancers [41]. However, some studies indicate that SMARCB1 can also influence SWI/SNF complex binding to super enhancers, although not as strongly as its role at typical enhancers [39, 115]. In contrast to first-level remodelers, cohesin has a profound impact on super enhancers. Super enhancers can be subdivided into hub and non-hub enhancers, with hub enhancers more frequently associated with cohesin and CTCF [116]. Cohesin is enriched at specific super enhancers that recruit master lineage transcriptional factors (TFs) to control distal target genes [117]. Thus, cohesin disruption causes super enhancers to co-localize and regulate gene transcription nearby [99]. Moreover, super enhancers are often strongly insulated by CTCF-anchored TADs [118]. In cancers, super-enhancers are frequently co-duplicated with strong TAD boundaries, a process that can lead to enhancer hijacking and the aberrant activation of oncogenes [118, 119].

### Outstanding question: Which remodeler mutations causally alter 3D genome architecture, rather than simply correlate with altered transcription?

To date, most studies have primarily linked mutations in first-level chromatin remodelers to altered gene expression, but direct evidence demonstrating that these mutations causally affect 3D genome architecture remains limited. Furthermore, the interplay between chromatin remodelers and the cohesin/CTCF axis in modulating 3D organization has not been systematically explained. Addressing these questions will require integrative approaches combining acute degradation systems with high-resolution 3D genomic methodologies.

## Regulation of chromatin remodeling

### Regulation by transcriptional modification

The interaction between chromatin remodelers and transcription factors is mutual. Many transcription factors require chromatin remodelers for effective genomic binding. For instance, the transcriptional repressor REST binds to SMARCA4, whereas the insulator protein CTCF binds to SNF2h [104]. Conversely, chromatin remodelers can be regulated and recruited to specific genomic loci by a pioneer transcription factor (PTF). Pioneer factors like FOXA1 bind to unwrapped nucleosomal DNA and increase chromatin accessibility by recruiting remodelers, including the NuRF complex. Indeed, BPTF, a scaffolding subunit of the NuRF complex, co-binds FOXA1 to promote androgen receptor (AR) activity [120]. Similarly, GATA3-dependent chromatin accessibility requires the catalytic activity of SWI/SNF ATPases [121]. ARID1A, the structural core of the SWI/SNF complex, binds folded Armadillo repeats on β-catenin, thereby interacting with multiple transcription factors and co-activators, including the nuclear receptor SF-1, TFs from the YAP, FOXO, and SOX families, and the histone acetyltransferase CBP/p300 [122].

In yeast, the RSC complex is directly recruited to DNA by a sequence-specific module; however, the lack of such a module in animal BAF complexes suggests they may rely more heavily on other cofactors and non-coding RNAs [123]. Non-coding RNAs regulate the ISWI complex by serving as guides to anchor it to chromatin or as scaffolds to assemble its subunits [46]. The SWI/SNF complex is recruited to both typical and super-enhancers by enhancer RNAs (eRNAs), which also recruit p300/CBP and Mediator to regulate certain gene transcription [124]. Furthermore, the BAF chromatin remodeler directly cooperates with RNA polymerase II and transcription factors to displace nucleosomes during transcription [125]. Corepressors such as REST also interact with chromatin remodelers like CHD4, which constitutes the NuRD complex, to collectively suppress tumor proliferation [126].

Cohesin likewise interacts with transcription factors and cofactors. Studies have pointed out that while cohesin and CTCF promote long-range interactions, cohesin also partners with mediators to bridge short-range interactions [127, 128]. eRNA can initiate transcription-induced supercoiling, which in turn drives cohesin-mediated loop extrusion and stimulates enhancer-promoter contacts [129]. Intriguingly, some studies identify RAD21 and CTCF themselves as transcriptional factors. RAD21 may directly interact with transcriptional repressors and recruit the NURD complex to suppress downstream signalling [130]. CTCF also drives transcription of telomeric repeat-encoding RNA (TERRA) to facilitate telomeric DNA replication [131].

### Regulation by post-translational modifications

Histone modifications are critical regulators of numerous biological processes, especially in DNA transcription. They function either by directly altering chromatin structure or by influencing regulatory effectors [132]. In the first mechanism, acetylation and phosphorylation of histone tail lysine neutralize their charge and directly affect electrostatic interactions with DNA to display general chromatin accessibility [133]. In the second, chromatin remodelers are the most important factors that interact with modified histones. Distinct domains on chromatin remodelers serve as histone mark readers to identify modified or unmodified histone tails. For example, the PHD domains of CHD family proteins recognize unmodified H3 tails and methylated H4 tails; the tandem chromodomain distinguishes tri-methylated H3K4, whereas the bromodomain of the SWI/SNF family specifically identifies acetylated residues such as H3K14ac [132, 134]. Bromodomains not only “read” the histone marks but also determine the time when remodelers bind to DNA. A prolonged binding event facilitates the recruitment of additional cofactors, triggering successive binding at the hotspot [135]. Some remodeling complexes contain multiple reader domains that synergize to recognize extensive histone marks. For instance, BAF45D, also known as DPF2, is a subunit of the BAF complex encompassing double plant homeodomains (DPF) that recognize H3K14 crotonylation and H3K56 acetylation [136, 137]. H3K56ac-BAF complex interaction indicates that histone globular domain modification participates in gene regulation. Additionally, dual recognition of acetylation and SUMO-histone conjugation contributes to SWI/SNF complex recruitment [138].

Modifications also happen on chromatin remodelers and affect their activity. In *Drosophila*, Cyclin E/CDK2, a cell cycle regulatory gene, mediates the phosphorylation of BRM/BRG1 to control the cell cycle and proliferation [139]. In mammals, neuronal stimulation induces serine phosphorylation of SWI/SNF ATPase SMARCA4. This modification alters its interactions with NuRD repressor complex and cohesin, thereby influencing transcription outcomes [140]. Apart from ATPase subunits, regulatory subunits also play a role in post-translational modification. For example, SMARCC2, a component of the BAF complex, can be phosphorylated by nuclear RIPK1, thereby mediating inflammatory responses [141]. CARM1-mediated methylation of SMARCC1 (also known as BAF155), a core component of the SWI/SNF complex, directs SMARCC1 to c-MYC pathway genes and promotes breast cancer progression and metastasis [142]. Meanwhile, DCAF5, a substrate receptor for the E3 ubiquitin ligase complex, promotes the degradation of aberrant cells, such as SMARCB1-mutated or SMARCC1/2-methylated cells, thereby maintaining chromatin assembly stability and suppressing tumor progression [143, 144]. Similarly, PTM profiling of CTCF revealed that O-GlcNAcylation regulates its role in organizing 3D chromatin architecture, highlighting an additional layer of post-translational control over genome organization [145].

### Outstanding question: How do remodeler complexes cooperate with lineage-specific transcription factors, super-enhancers, and phase-separated nuclear condensates?

Although studies have already shown that first-level remodeler complexes have been shown to interact with lineage-specific transcription factors and are recruited to enhancers to exert their regulatory functions. Moreover, certain subunits of these complexes that contain prion-like domains (PLDs) are likely to undergo intracellular phase separation [146]. There is still an outstanding question, which is how these distinct processes are coordinated to achieve precise gene regulation.

## Mechanisms of chromatin remodeling disorders in cancer

**Fig. 4:**
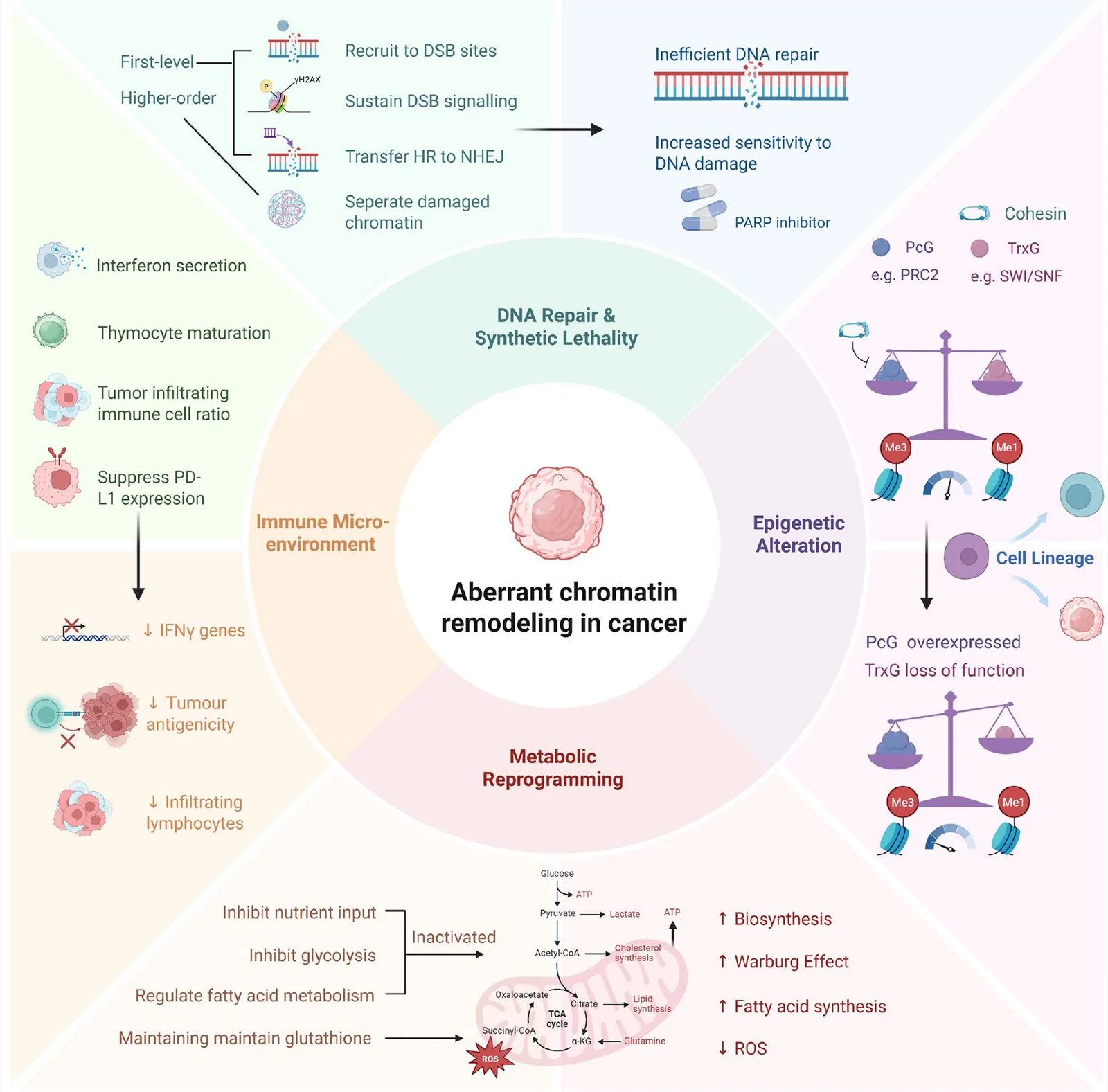
Chromatin remodeling disorder in cancer. (Figure updated). Aberrant chromatin remodeling in cancer. Chromatin remodeling plays a critical role in DNA repair. First-level chromatin remodelers are recruited to double-strand break (DSB) sites, sustain DSB signaling, and can shift repair from homologous recombination (HR) to non-homologous end joining (NHEJ), thereby facilitating DNA repair. Higher-order chromatin remodeling promotes DNA repair by segregating damaged chromatin. Aberrant chromatin remodeling leads to inefficient DNA repair and increases tumor cell sensitivity to DNA damage. Therefore, in tumors harboring inactivating mutations in chromatin remodelers, combination therapy with PARP inhibitors can achieve synthetic lethality. At the epigenetic level, chromatin remodelers, together with cohesin, maintain the balance between Polycomb group (PcG) and Trithorax group (TrxG) proteins, thereby sustaining appropriate chromatin accessibility. In cancer cells, this balance is often disrupted—for example, through overexpression of PcG proteins or inactivation of TrxG proteins—leading to a marked increase in nucleosome methylation and altered transcription of tumor suppressor genes, which further determine cell lineage. Chromatin remodelers can suppress aberrant metabolism in tumor cells. However, when inactivated, they may promote biosynthesis, enhance the Warburg effect, and increase fatty acid synthesis, thereby providing nutrients for tumor cell proliferation while reducing the impact of reactive oxygen species (ROS) on cells. In the context of immunity, chromatin remodelers are involved in cytokine secretion, thymocyte maturation, tumor-infiltrating lymphocytes, and PD-L1 expression. Chromatin remodelers may promote immune evasion by downregulating interferon (IFN) gene transcription, suppressing tumor antigen presentation, and inhibiting lymphocyte infiltration

As presented in Fig. 4, aberrant chromatin remodeling can lead to carcinogenesis in multiple ways, including dysregulation of DNA repair, epigenetic regulation, metabolic reprogramming, and the immune microenvironment.

### Cell lineage specification

Across multiple cancer types, SWI/SNF complexes are required for core regulatory circuits to sustain DNA accessibility. In hematological malignancies such as acute myeloid leukemia and lymphoma, SWI/SNF is recruited by the pioneer transcription factor PU.1 to increase accessibility for core regulatory factors, thereby maintaining the stemness of leukemic cells and deciding B cell fate during the germinal center (GC) response [147, 148]. In lymphoma, loss of ARID1A skews B-cell fate toward immature memory B cells, prone to an aggressive transformation [148]. In fusion-positive rhabdomyosarcoma, SMARCA4 colocalizes with the PAX3-FOXO1 fusion protein at enhancer regions, preventing myogenic differentiation [149]. In uveal melanoma, SWI/SNF cooperates with the lineage-defining transcription factor MITF to drive tumor survival [150]. In neuroblastoma, SWI/SNF ATPase facilitates lineage plasticity and invasiveness by increasing the chromatin accessibility of mesenchymal genes [151].

SWI/SNF is recruited to core enhancer regions through multiple mechanisms. A common mechanism in hematological malignancies is the ordered, sequential interactions among SWI/SNF, pioneer transcription factors PU.1, and downstream transcription factors, which have been shown to contribute to pathogenesis like AML and diffuse large B-cell lymphoma, as well as normal lymphoid and myeloid differentiation [147, 148]. In addition, β-catenin functions as a molecular adapter by directly binding to ARID1A and connecting to multiple transcriptional regulators in adrenal cancer, forming a broadly applicable modular recruitment network [122]. Notably, in the eRNA-mediated cis-recruitment mechanism, the SWI/SNF complex binds to eRNA through an AT-hook and is directed to stage-specific enhancers and super-enhancers to regulate lineage-specific genes [124]. Based on the interaction between SWI/SNF and core regulatory circuit enhancers, targeting this interaction is a primary outcome of SWI/SNF inhibition in multiple cancer types. Noteworthily, the regulation of core regulatory circuit enhancers by SWI/SNF exhibits temporal dependency, with peak chromatin accessibility during G1 phase, indicating a therapeutic strategy of SWI/SNF inhibition combined with retinoids to induce cell-cycle exit [152].

### DNA repair malfunction

Genome instability is the most prevalent hallmark of cancer. The repair of double-strand breaks (DSBs) is of utmost importance for maintaining genome integrity, including two major methods: homologous recombination (HR) or non-homologous end joining (NHEJ) [153]. DNA damage repair (DDR) requires regulated access to specific DNA sequences, aberrant chromatin remodelers disrupt HR or NHEJ, increasing cellular sensitivity to DNA damage. Different chromatin remodelers play distinct roles in facilitating DDR. Specific subunits of remodeling complexes, including SMARCA4 and ARID1A/B in the SWI/SNF family, and SNF2h and SNF2l in the ISWI family, are rapidly recruited to DSB sites to mediate the DDR response [27, 154, 155]. Meanwhile, CBP/p300, the homologous H3 and H4 acetyltransferases, contribute to the recruitment of SMARCA2 to DSB sites, facilitating chromatin relaxation [156]. ARID1A is essential for processing DSBs into single-strand ends and sustaining DSB signaling, thereby promoting HR pathways [157]. Mediated by SIRT6, CHD4-dependent chromatin relaxation is another critical component for accurate HR [158].

The above vulnerabilities underpin the therapeutic rationale for poly(ADP-ribose) polymerase inhibitors (PARPi). Notably, PARPi can also induce immediate histone eviction via the INO80 complex, prevent the accumulation of DNA damage, and thus contribute to PARPi resistance [159]. ALC1, also known as CHD1L, is an oncogene-encoded enzyme that can be recruited to DNA damage sites by interacting with poly(ADP-ribose) [160]. ALC1 deficiency sensitizes HR-deficient cancer cells to PARP inhibitors by reducing chromatin accessibility and impairing DNA repair [161].

The strategy of synthetic lethality also applies to cohesin mutations. Notably, cohesin plays a dual role in DDR. While it normally helps separate damaged chromatin, ATM phosphorylation can trigger cohesin to enhance repair efficiency [162]. During a DSB event, TADs are instrumental for DDR by creating a specific repair-prone chromatin compartment. At the DBS site, cohesins are blocked by repair complexes, and cohesin-mediated loop extrusion becomes unidirectional, which in turn facilitates ATM-mediated phosphorylation of H2AX and enables an efficient and rapid response to DSB [163]. Meanwhile, cohesin binds at DNA replication origins and facilitates replication fork progression dynamically [164, 165]. In cohesin-mutated tumor cells with stalled replication forks, PARP inhibition prevents the recruitment of necessary proteins, impeding fork regression, ultimately leading to fork breakage and the formation of a DSB [166].

Given that SWI/SNF inactivation causes inefficient DSB repair and increased sensitivity to DNA damage, it has been hypothesized that it drives tumor progression mainly through impaired DDR [167]. This hypothesis, however, is challenged by findings that restoring SNF5 in a cell line deficient in SNF5 can induce cell cycle arrest [168]. Besides, the oncogenic effects of SMARCB1 inactivation appear cell-type-specific, primarily driving cancer from memory CD8 + T cells but not from B cells or other T-cell subsets, highlighting the context-dependent nature of SWI/SNF-mediated tumor suppression [169]. Current models therefore support the coexistence of DDR and epigenetic mechanisms in SWI/SNF function, based on their established roles in enhancer regulation [41].

### Epigenetic dysregulation

Both first-level chromatin remodeling and cohesin-mediated higher-order remodeling are essential to cancer development. Previous studies in *Drosophila* foreshadowed the importance of the PcG–TrxG balance in cancer pathogenesis. In brief, Polycomb group (PcG) proteins promote transcriptional silencing through histone methylation and ubiquitination, while Trithorax group (TrxG) proteins act as their natural antagonists [170]. The balance between these opposing activities determines the methylation status of H3K27. For example, mutations in Polycomb repressive complex 2 (PRC2), a key component of PcG proteins, frequently co-occur with mutations in SWI/SNF subunits, which are classified as TrxG proteins. Such combined disruption consistently leads to aberrant H3K27 trimethylation and promotes tumorigenesis [171, 172]. Cohesin also regulates PRC2 activity, either by directly recruiting it to chromatin through RAD21 or by facilitating distal interactions through STAG2 [173, 174].

### Metabolic abnormal reprogramming

In order to overcome waste accumulation and nutrient depletion, cancer cells undergo tissue-specific metabolic adaptation [175]. Chromatin remodelers like SWI/SNF normally function to inhibit the influx of nutrients into metabolic pathways. When ARID1A is inactivated, GLS1 is upregulated, increasing glutamine flux into the TCA cycle, promoting biosynthesis, and thus driving tumor growth in clear cell ovarian carcinoma [176]. Deficiency of BPRM1, a subunit of the PBAF complex, promotes glycolysis through the AKT-mTOR pathway in clear cell renal carcinoma, contributing to tumor progression with the “Warburg Effect” [177, 178]. In lung cancer, SMARCA4 mutation enhances oxidative phosphorylation (OXPHOS) and increases oxygen consumption, suggesting that OXPHOS inhibitors may represent a potential therapeutic strategy [179]. In breast cancer cells, however, SMARCA4 rewires metabolism through fatty acid synthesis [180]. Meanwhile, ARID1A regulates lipid metabolism in liver cancer by controlling chromatin accessibility at genes involved in lipid β oxidation [181]. Besides, SNF2L is always elevated in cancer cells, where it helps maintain glutathione (GSH) levels. Targeting SNF2L may therefore increase reactive oxygen species (ROS) and induce greater oxidative stress [182].

### Immune microenvironment disorder

Importantly, chromatin remodeler alterations can have different immunologic consequences, either promoting immune activation and ICI sensitivity or reinforcing immune exclusion and resistance, depending on the subunit and tumor context. Avoiding immune destruction is a hallmark of cancer and promotes the development of immunotherapy [183]. By blocking the immune checkpoint with targeting molecules, such as PD-1 and PD-L1, an immunosuppressive microenvironment can be reactivated, enhancing the tumor-killing effect [184]. Both the first-level and higher-order chromatin remodeling influence the immune microenvironment through several mechanisms: silencing IFN-γ genes, activating tumor antigenicity, and suppressing PD-L1. The SWI/SNF subunit ARID1A promotes immune activation by preventing EZH2 and PRC from binding to IFN-responsive genes, thereby facilitating chemokine expression and anti-tumor immune signaling, whereas SMARCB1 and SMARCA4 modulate these same genes through interactions with MYC [185]. ISWI-family remodelers are also correlated with the tumor microenvironment, with multiple ISWI members involved in regulating immune-related genes. For example, NURF deficiency enhances CD8 + T cell activity and improves antigen processing [186]. Conversely, the BPTF-NURF complex promotes the maturation of CD4 + and CD8 + cells into T cells by recruiting NURF to specific TF binding sites [187]. More broadly, multiple ISWI members are associated with the tumor-infiltrating immune cell ratio in the tumor microenvironment (TME), and thus influence immune response outcomes [46]. CHD1 functions as an important TME modulator in prostate cancer, where it promotes the accumulation of myeloid-derived suppressor cells and suppresses infiltrating lymphocytes together with IL6 [188]. Cohesin-mediated remodeling also influences immune phenotypes in a context-dependent manner. For example, cohesin-mediated chromatin remodeling affects TME through STAG2, a regulatory subunit of cohesin. STAG2 inactivation enlarges TADs, facilitating long-range interactions between a distal enhancer and the IRF9 promoter, which in turn regulates interferon-response genes and PD-L1 expression in melanoma [189]. Conversely, RAD21 and CTCF have been shown to suppress surface expression of PD-L1 and MHC-I, although the underlying mechanism is still unclear [190]. In the following section, we will delineate cancer-type-specific roles of chromatin remodeling in each malignancy.

### Controversies of chromatin remodelers

Several outstanding controversies highlight the context-dependent roles of chromatin remodelers in cancer. This likely depends on cancer types, lineage, differential reliance on enhancer regulation versus genome maintenance, and residual complex composition.

First, the functional roles of SWI/SNF subunits have not been firmly established. ARID1A is one of the most extensively characterized context-dependent subunits. ARID1A is commonly mutated in most tumor types and functions primarily as a tumor suppressor [191]. In contrast, ARID1A acts as an oncogene in tumor initiation, but as a tumor suppressor in subsequent maintenance and metastasis in liver cancer [192]. ARID1A loss influences chromatin organization at multiple levels and promotes invasion, metabolic reprogramming, or metastasis in certain malignancies or during progression, while in other contexts it triggers DNA damage-mediated immune activation [100, 192, 193].

Secondly, there are controversial studies indicating differential roles of chromatin remodelers in transcription regulation. For example, one study proposed that SMARCB1 would only affect the binding of the SWI/SNF complex to regular enhancers but not to super-enhancers [41]. While another investigation suggested that SMARCB1 would also influence the binding between the SWI/SNF complex and super-enhancers [194]. The contradictory findings could be cell context-dependent; this is why SMARCB1 is widely recognized as a tumor suppressor gene in rhabdoid tumors [195], but its functional consequences in other malignancies are shaped by cell type and enhancer context [196].

Thirdly, cohesin mutations mainly cause abnormalities in downstream transcriptomes and epigenomes by altering the 3D chromatin structure (such as affecting DNA looping and TAD structures). The functions, mutation timing, and effects of its subunits and regulatory factors vary significantly among different cancer types [197]. Although cohesin is classically described as tumor suppressive, therapeutic exploitation of cohesin defects is complicated by its essential role in normal cells. This creates a therapeutic problem: complete cohesion inhibition is not clinically feasible, whereas partial disruption or paralog-specific vulnerabilities may be exploitable.

At last, SWI/SNF alterations are associated with either immune activation and ICI sensitivity or immune exclusion and resistance, depending on tumor context. Mutations in SWI/SWF genes confer one of the most notable vulnerabilities currently recognized, which is sensitivity to immune-checkpoint inhibition (ICI) [27]. SWI/SNF loss increases DNA damage, cGAS-STING, and IFN signaling [193]. We summarized the controversial roles of chromatin remodelers in specific types of cancers in the following sections.

### Outstanding question: What determines whether a remodeler functions as a tumor suppressor or an oncogene in a given cancer type?

A critical challenge in the field lies in the dual roles of chromatin remodelers in tumorigenesis, raising the fundamental question: What determines whether a given remodeler functions as a tumor suppressor or an oncogene in a specific cancer type? For example, in rare rhabdoid sarcoma, the BAF complex with SMARCB1 inactivation loses the ability to evict polycomb at proliferation-repressive loci, which establishes the tumor-suppressive function of the BAF complex [171, 198]. Paradoxically, in synovial sarcoma, the SS18-SSX fusion protein of BAF complexes causes the displacement of SMARCB1, targeting the aberrant complex to oncogenic loci and exerting an oncogenic effect [199, 200]. Thus, the same BAF complex can drive tumor suppression or promotion depending on the molecular context, highlighting the importance of understanding the determinants of remodeler function for precision targeting.

## Dysregulation of chromatin remodeling and potential therapies in specific cancers

**Fig. 5:**
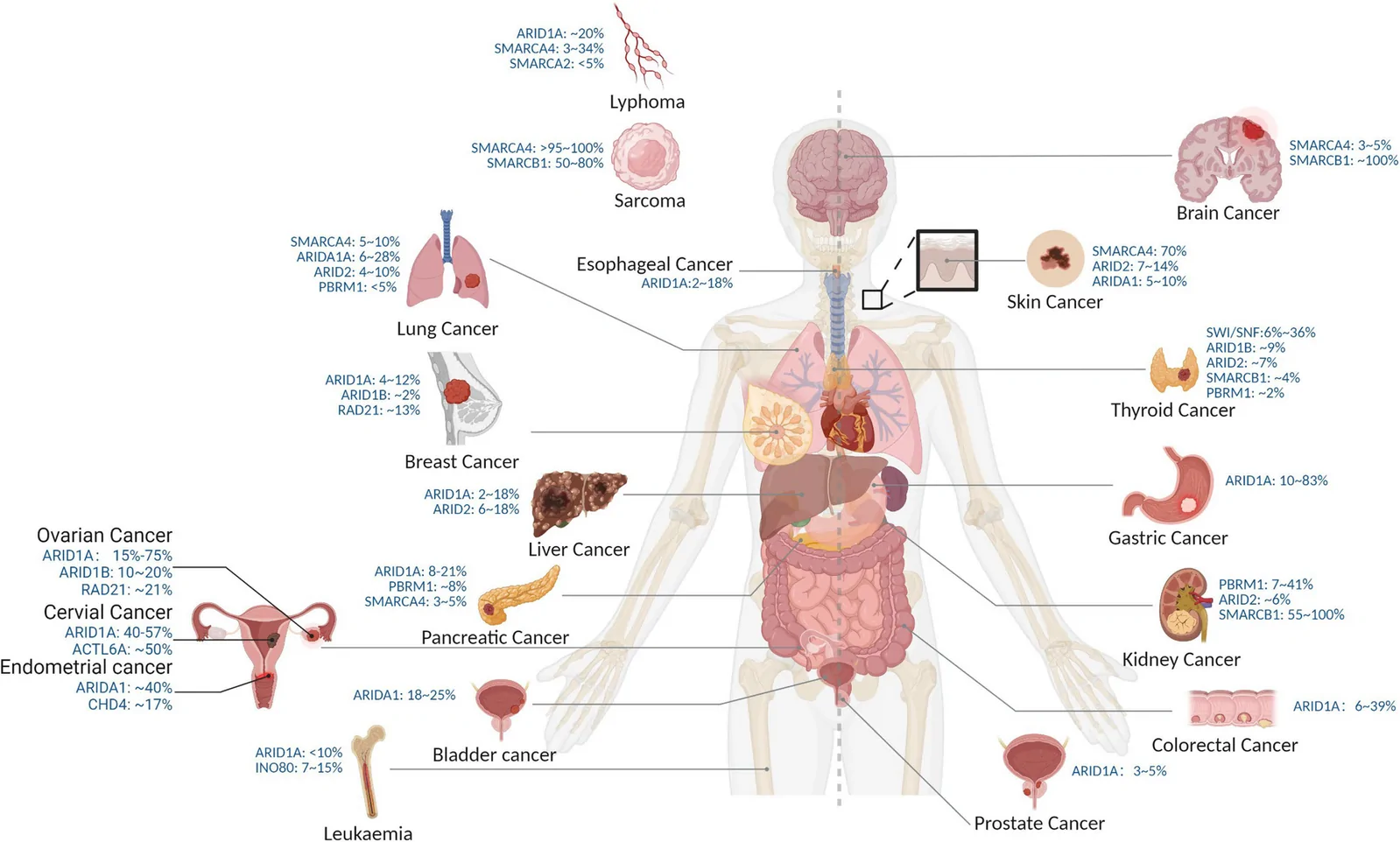
Mutation incidence of Chromatin remodelers in cancer. Mutational landscape of chromatin remodeler subunits across various human cancers. The panel summarizes the mutation frequencies of chromatin-remodeling subunits across common malignancies arising from different anatomical sites

Genes encoding chromatin remodelers are frequently mutated across a variety of cancer types; common mutations and their incidence are summarized in Fig. 5. Across different systems, the mutated chromatin-remodeling subunits vary.

### Digestive system

#### Gastric cancer

Gastric cancer is a major global health challenge, ranking among the most prevalent and lethal malignancies across the globe [201]. Through exome sequencing, Wang et al. [202] identified frequent *ARID1A* mutations in gastric cancer, particularly in microsatellite instability (MSI)-high (83%) and Epstein-Barr virus (EBV)-associated subtypes (73%), compared to only 11% in EBV-negative and MSS cases. In gastric carcinogenesis, ARID1A functions as an early, truncal driver event. Genomic analysis of paired primary and peritoneal metastatic gastric cancer lesions revealed shared *ARID1A* mutations between primary and metastatic sites. Whole-genome sequencing further detected *ARID1A* mutations in morphologically normal or metaplastic gastric glands, indicating that its inactivation contributes to initial tumorigenesis rather than representing a metastasis-specific alteration [203, 204]. *ARID1A* mutations are significantly associated with MSI and *PIK3CA*-activating mutations, and most *ARID1A*-mutated tumors harbor wild-type *TP53 *[203]*.* In Epstein-Barr virus-associated gastric cancer (EBVaGC), *ARID1A* is one of the most frequently altered genes, mutated in approximately 17% of cases and displaying a significantly higher proportion of deleterious mutations (e.g., frameshift and stop‐gained) compared to EBV-negative gastric cancer (EBVnGC) [205].

These LOF mutations, particularly EBV-associated subtypes, directly trigger the CpG island methylator phenotype by compromising SWI/SNF chromatin remodeling, leading to promoter hypermethylation and silencing of tumor suppressor gene [206].

ARID1A deficiency also activates mTOR signaling (pS6 upregulation) and induces SOX9 nuclear expression, driving tumor invasion and metastasis [207]. In CRISPR/Cas9-engineered ARID1A-deficient human gastric organoids, ARID1A loss exhibits dual oncogenic roles: non-essential Wnt-inhibited mucinous differentiation and essential FOXM1/BIRC5-driven proliferation, offering selective vulnerability to surviving inhibitors [208]. However, a recent study has elucidated the dosage-dependent roles of Arid1a in gastric cancer, in which heterozygous loss (*Arid1a*^±^) promotes tumorigenesis through enhancer dysregulation and suppression of the p53 pathway, while homozygous deletion (*Arid1a*^−/−^) paradoxically inhibits tumor growth via excessive p53 activation [209]. Beyond ARID1A, other chromatin remodeling alterations contribute to gastric cancer pathogenesis. Although SMARCA1 is abundantly expressed in normal gastric tissues, it is abnormally methylated in both non-cancerous and cancerous gastric tissues [210]. HLTF methylation is observed in 38% of gastric cancer cases [211], and BRM deficiency is also prevalent, with immunostaining of 89 primary tumors showing reduced expression in 67% and severe loss in 42% [212].

In contrast to the tumor-suppressive role of ARID1A, ACTL6A, which is a subunit of the SWI/SNF-related BAF complex, exerts pro-tumorigenic effects by enhancing antioxidant defenses in gastric cancer. Upregulated ACTL6A cooperates with NRF2 to transcriptionally upregulate *GCLC*, thereby promoting GSH synthesis and suppressing ferroptosis in gastric cancer cells [213].

Collectively, these findings implicate disruption of chromatin remodeling and epigenetic regulation, including *ARID1A* inactivation, *BRM/SMARCA1* and *HLTF* methylation, BRM deficiency, and ACTL6A-driven antioxidant programs, as key, potentially targetable contributors to gastric cancer pathogenesis and progression.

#### Liver cancer

Dysregulation of chromatin remodeling in hepatic cancer exhibits a paradoxical nature, functioning as both a tumor-suppressive mechanism and a driver of malignant progression. Mutations in *ARID1A* have been identified in hepatocellular carcinoma (HCC) through whole-exome sequencing (WES) and whole-genome sequencing (WGS) studies [214, 215]. In a cohort of 64 paired HCC and adjacent nontumorous tissue samples, ARID1A expression was significantly lower in HCC tissues and strongly associated with increased metastasis, including local lymph node and distant metastasis, as well as poor prognosis [216]. *ARID1A* mutation is particularly prevalent in hepatitis B virus (HBV)-positive Individuals with HCC [217]. Similarly, *ARID2* LOF mutations have been reported in HCC, occurring in 18.2% of HCV-associated cases in the United States and Europe, and ARID2 expression has been positively correlated with a good prognosis [218, 219]. BRG1 expression is upregulated in most human HCC samples and correlates with poor prognosis; however, SMARCA4 mutations or low BRG1 expression can also be found in a small percentage of human HCCs [220].

ARID1A deficiency promotes HCC progression through aberrant deposition of histone H3K27ac at the angiopoietin-2 (Ang2) enhancer and promoter to drive angiogenesis [221]. Additionally, it induces HCC metastasis by impairing the BRG1-RAD21 interaction, ultimately resulting in chromatin reorganization, including A/B compartment switching, TAD remodeling, and loop loss, which dysregulates pro-metastatic genes such as PMP22 and GSC [100]. E-cadherin levels strongly correlate with ARID1A expression, suggesting that they interact to regulate migration and invasion [216]. Another study demonstrated that ARID1A exhibits context-dependent dual roles in HCC: it suppresses migration, invasion, and metastasis in established tumors through SWI/SNF-mediated chromatin remodeling, yet promotes tumor initiation by driving oxidative stress via CYP450 enzymes [192]. ARID2 loss promotes hepatocarcinogenesis by compromising nucleotide excision repair (NER), leading to inefficient removal of carcinogen-induced DNA lesions and a resultant hypermutator phenotype in HCC [222]. *ARID2* mutations, particularly those disrupting the C2H2 zinc finger domain, prevent its metastasis suppressor function in HCC by impairing DNMT1 recruitment to the Snail promoter, thereby reducing promoter methylation, derepressing Snail transcription, and activating EMT, especially in sarcomatoid HCC [219, 223]. ARID2 mutations in liver cancer may also alter the chromatin structure of liver-specific enhancers and reduce chemokine expression [224]. Overexpression of SMARCA4 promotes transcriptional activation of IRAK1, inducing the oncoprotein Gankyrin and AKR1B10 to drive malignant transformation, growth, and metastasis of HCC cells [223]. Meanwhile, hepatic SMARCA4 overexpression is tissue-specific; it activates *IRAK1* enhancers to upregulate Gankyrin/AKR1B10 and enhances *SMAD6* transcription via HIC2/NR4A1, thereby attenuating TGF-β/SMAD signaling and driving HCC cell proliferation and postoperative recurrence [225]. Further studies have revealed that SMARCA4 (BRG1) exhibits dual oncogenic and tumor-suppressive roles depending on upstream oncogenic stimuli. It is essential in c-MYC-driven HCC, whereas its loss synergizes with c-MET or NRas (NRAS^V12^) activation to induce HCC in RAS/MAPK contexts [220]. On the other hand, SMARCB1 overexpression in HCC, scaffolded by NUP210 at the nuclear periphery, drives tumorigenesis via chromatin remodeling dysregulation [196].

#### Pancreatic cancer

Pancreatic ductal adenocarcinoma (PDAC) is among the most malignant tumors, characterized by an extremely poor prognosis [226]. Among 59 undifferentiated carcinomas, 30 cases (51%) showed loss of SWI/SNF subunits, primarily involving SMARCB1 (INI1), SMARCA4 (BRG1), and SMARCA2 (BRM), predominantly manifesting as rhabdoid morphology, aggressive behavior, and poor prognosis as determined by immunohistochemistry [227]. Compared with normal tissues, BRM mRNA is overexpressed in PDAC and correlates significantly with higher tumor histologic grade and worse overall survival [228]. Deep whole-exome sequencing of 150 PDAC specimens demonstrated recurrent somatic mutations in *ARID1A* and *PBRM1* [229]. A study analyzing real-world genomic profiles from 1,080 Chinese pancreatic cancer patients identified recurrent *ARID1A* mutations at a frequency of 12.8% [230]. Notably, *ARID1A* co-mutations are significantly enriched in *KRAS* G12C-mutated PDAC compared to non-G12C *KRAS* mutations (21% vs 8%, *P* = 0.014) [231].

Dynamic changes in BRG1 and BRM expression occur across different stages of pancreatic intraepithelial neoplastic lesions (PanIN) and PDAC. Specifically, BRG1 (SMARCA4) loss promotes pancreatic cancer stemness, invasion, and metastasis by activating the hypoxia signaling pathway in PDAC [232]. Interestingly, BRG1 exhibits tumor-suppressive functions during intraductal papillary mucinous neoplasm (IPMN) initiation by maintaining ductal identity, whereas it becomes oncogenic in established PDAC by promoting EMT and desmoplasia [233]. BRG1 overexpression mediates both intrinsic and acquired gemcitabine resistance via a positive feedback loop with Akt signaling, where BRG1 enhances Akt phosphorylation and p21 upregulation, while activated Akt reciprocally sustains BRG1 expression [234]. Similarly, BRM overexpression activates the JAK2/STAT3 pathway, promoting pancreatic cancer growth and chemoresistance [228]. *ARID1A* mutations in pancreatic cancer exhibit transcription start sites (TSS)-specific mutagenesis patterns [235]. ARID1A functions as a tumor suppressor barrier in pancreatic acinar cell homeostasis. Its loss promotes tumorigenesis by disrupting acinar regeneration, inducing acinar-to-ductal metaplasia (ADM), activating EMT and stemness programs that enhance migration, invasion, and low-grade tumor formation [236]. In particular, Arid1a deletion cooperates with mutant Kras/p53 to induce IPMN/PDAC from dedifferentiated pancreatic ductal cells (PDCs) via Sox9-mediated suppression of differentiation and mTOR activation, a cellular origin distinct from that of Smad4/Tff2-deficient IPMNs [237]. Furthermore, ARID1A loss induces gene networks associated with MYC activity in a compartment-specific manner [238]. *RBRM1* mutations do not simply promote proliferation; instead, they derepress vimentin through epigenetic mechanisms, driving tumor dedifferentiation, EMT, and metastasis in PDAC [239]. SA1 depletion alters *Reg* locus architecture, contributing to PAC development via Reg-mediated inflammation [240]. Low PDS5B expression in PC regulates cell proliferation and motility via downregulating Ptch2 [241].

#### Esophageal cancer

Esophageal carcinoma is ranked the 11th most common cancer worldwide [242]. It is broadly classified into esophageal adenocarcinoma (EAC) and esophageal squamous cell carcinoma (ESCC), which exhibit distinct mutational profiles.

EACs show more frequent alterations in SWI/SNF-encoding genes (AR1D1A, SMARCA4, PBRM1), whereas ESCCs are associated with mutations in histone-modifying factors (KDM6A, KMT2D, KMT2C) [243]. Somatic mutations in SWI/SNF complex components are thought to arise early in esophageal cancer development [244]. These molecular differences reflect the underlying pathophysiology divergence between subtypes and suggest distinct therapeutic approaches. In EAC, *BRM* polymorphism (double homozygotes) and loss of ARID1A protein are associated with worse overall survival [245, 246]. ARID1A is abundantly expressed in normal esophageal epithelium but is absent from the basal layer, implicating its loss in the initiation of squamous cell carcinoma. Indeed, ARID1A depletion elevates stemness properties and tumor initiation [247]. Furthermore, the *ARID1A* mutation correlates with enhanced tumor-infiltrating CD8 + T cells and enriched IFN signaling in vitro, suggesting immunomodulatory roles for the SWI/SNF complex in EAC [248]. Mechanistically, ARID1A modulates lipid metabolism in EAC, thereby regulating CD8 + T cell recruitment and cytolytic activity [248]. A recent pan-cancer analysis of next-generation sequencing data revealed that SWI/SNF mutations in esophageal cancer are strongly associated with high tumor mutational burden (TMB-H) and can predict better immunotherapy response [249]. In ESCC, the synthetic lethal interaction between SMARCA2 and SMARCA4 highlights SMARCA4 as a novel therapeutic target in SMARCA2-deficient malignancies [250]. Since radiotherapy is the primary treatment option for ESCC, the role of the SWI/SNF complex in radiosensitivity has garnered interest. In ESCC patients receiving chemoradiotherapy, low ACTL6A protein expression is significantly associated with improved clinical outcomes [251]. The group found that ACTL6A knockdown in irradiated cells elevates the γH2AX protein, an initiating signal for DNA repair, which is rapidly dephosphorylated after DNA repair completion. The accumulation of γH2AX suggests that targeting ACTL6A impairs radiation-induced DNA repair and enhances ESCC radiosensitivity [251].

Alterations in ISWI family subunits are less documented in esophageal carcinoma. Amplification of *BAZ1A* in cell lines and primary tumors revealed a possible association with the development and progression of ESCC [252]. In support of this, RSF1 is elevated significantly in ESCC and inversely correlates with disease-free survival [253].

To our knowledge, there is limited characterization of CHD and INO80 chromatin remodeling complexes in esophageal carcinoma, and hence, they are not discussed in this section.

#### Colorectal cancer

Colorectal cancer (CRC) remains one of the most common and lethal malignancies worldwide. In a cohort of 47 colorectal medullary carcinoma (MeC) cases, low ARID1A expression was observed in 39.02% of patients [254]. Concurrently, CRC tumors with ARID1A functional impairment exhibit intrinsic and acquired resistance to anti-EGFR therapies such as cetuximab [255]. In the combined MD Anderson and The Cancer Genome Atlas (TCGA) cohorts comprising 502 CRC cases, non-silent ARID1A mutations were observed in 56 cases (11.1%). Among 419 microsatellite-stable (MSS) CRC patients, 28 (6.7%) harbored such mutations, which strongly correlated with IFN-γ expression [256]. Promoter hypermethylation is also a frequent molecular alteration in CRC. Prognostically, HLTF methylation significantly associates with larger tumor size, metastatic disease, advanced tumor stage, and reduced survival probability [257, 258]. SMC1A expression increases during colorectal tumorigenesis, and analysis of 172 CRC surgical specimens revealed that high STAG3 expression correlates with poor prognosis [259, 260].

At the mechanistic level, ARID1A deficiency upregulates CHK1 via DDB1-mediated ubiquitination, leading to cytosolic single-stranded DNA accumulation [261]. In *Arid1a*^*f/f*^ mouse models**,** intestinal ARID1A deletion disrupts epithelial homeostasis through *Sox9* overexpression [262]. In colon cancer models, ARID1A loss impairs SWI/SNF-mediated enhancer regulation by dominant transcription factors, resulting in widespread gene expression dysregulation [40].

Several studies position SMARCA4 (also known as BRG1) as a tumor suppressor in CRC. A hotspot mutation in *SMARCA4*, resulting in an arginine-to-tryptophan substitution at residue 1157 (R1157W), facilitates its recruitment to PRMT1‑mediated H4R3me2a (asymmetric demethylation of Arginine 3 on histone H4) and enhances the chromatin recruitment and remodeling activity of the SWI/SNF complex, thereby accelerating colorectal cancer progression through upregulation of EGFR signaling [263, 264]. Meanwhile, SMARCA4 deficiency disrupts transcription of autophagy-related genes, impairs autophagosome formation, elevates ROS levels, and compromises intestinal barrier integrity [265]. Decreased BRG1 expression further activates the miR-550a-5p/RNF43/Wnt/β-catenin pathway to drive CRC metastasis in vitro and in vivo, while also inducing senescence via the SIRT1/p53/p21 axis [266, 267]. In CRC cell lines and patient-derived organoids, downregulation of SMARCA3 leads to concurrent loss of H3K23 ubiquitination and H3K9me3, resulting in widespread transcriptional changes and the upregulation of oncogenic pathways such as FABP6 [268]. Ectopic expression of the SWI/SNF subunit BAF53A promotes proliferation, colony formation, and tumorigenesis through the BAF53A-DUSP5-ERK1/2 and BAF53A-KLF4-p53 axes [269, 270].

Functional inactivation of SMARCA1, mediated by lncRNA MIR99AHG/PTBP1-directed aberrant splicing that generates the LOF isoform SMARCA1-L, impairs its transcriptional regulation of the NPFF promoter, thereby upregulating NPFF expression. Consequently, elevated NPFF engages autocrine JAK2/STAT5 signaling to drive epithelial-mesenchymal transition (EMT) in tumor cells and reprograms tumor-associated macrophages towards an M2-like phenotype, ultimately promoting colorectal cancer metastasis [271, 272]. SMC1A promotes CRC progression by recruiting and activating tumor-associated fibroblasts (TAFs) and upregulating inflammatory and matrix-remodeling factors, thereby reshaping the tumor microenvironment to enhance invasion and metastasis [273].

### Reproductive system

#### Ovarian cancer

Ovarian cancer is the 5th leading cause of cancer death among females [274]. Approximately 90% of ovarian cancers are of epithelial origin and can be categorized into 6 subtypes: high-grade serous, endometroid, clear cell, low-grade serous, mucinous, and carcinosarcoma [275].

SWI/SNF alterations in ovarian cancers are frequent, with the most common mutations occurring in *AR1D1A, SMARCA4*, and *AR1D1B* [276]. In ovarian clear cell carcinoma and ovarian endometroid carcinoma, *ARID1A* LOF mutations and subsequent ARID1A protein loss have been reported in 30–75% and 15–75% of cases, respectively [277]. In ARID1A-inactivated ovarian clear cell carcinoma, mevalonate pathway inhibition of the combined with immune checkpoint blockade showed synergistic anti-tumor activity by promoting pyroptosis [278]. High *SMARCA4* mRNA is associated with favorable progression-free survival (PFS) and OS of ovarian cancer patients [279]. Almost all cases of small cell carcinoma of the ovary, hypercalcemic type (SCCOHT) exhibit activating biallelic mutations in *SMARCA4* [280, 281]. SCCOHT is rare but aggressive and is categorized under ‘miscellaneous tumors’ of ovarian cancer according to the World Health Organization (WHO) Classification of Tumors of the Female Reproductive Organs (4th edition) [282].

Beyond SMARCA4, cohesin gene alterations are common in ovarian cancers, with shallow and deep deletions collectively affecting 95% of cases [283]. *RAD21* amplification is observed in 21% of ovarian cancer patients, and high RAD21 protein expression correlates with poor prognosis [130, 284]. By interacting with YAP/TEAD4-corepressor, RAD21 can remodel chromatin and exert suppressive roles in IFN signaling and T-cell activation [130]. Furthermore, it promotes ovarian cancer progression by activating the Akt/mTOR pathway [284]. *RSF1* gene amplification is similarly implicated in ovarian cancer and is correlated with poor overall survival [285]. The RSF1/SMARCA5 complex drives chemoresistance by acting as a coactivator of NFκB, thereby enhancing antiapoptotic gene expression [286, 287]. CHD family alterations have also been implicated in ovarian cancer. Missense mutations and promoter methylation of *CHD5* suggest a tumor-suppressor role for the CHD complex in ovarian cancer [288]. In accordance with this, downregulation of CHD5 or CHD4 is associated with poor clinical outcomes [289, 290]. In *BRCA2*-mutant cells, reduced CHD4 expression drives cisplatin resistance in a RAD18-dependent manner, which may contribute to unfavorable prognosis [290]. To date, alterations of INO80 complex subunits in ovarian cancers have remained insufficiently described. A recent study reveals that high INO80 expression is significantly associated with poor clinical outcomes and is less likely to benefit from immunotherapy [291].

#### Prostate cancer

Prostate cancer (PCa) is the 4th most common cancer (Globocan 2022) [242]. The primary management strategy for low-risk prostate cancers is active surveillance since they are unlikely to progress. For intermediate to very high-risk prostate cancers, treatment options include surgery, brachytherapy, and external-beam radiotherapy (EBRT) with or without androgen deprivation therapy (ADT) [292]. ADT remains the mainstay treatment for metastatic PCa; however, treatment resistance frequently develops, accompanied by progression to castration-resistant prostate cancer (CRPC) [292]. While typically dependent on androgen-receptor signaling, CRPC retains an adenocarcinoma phenotype (CRPC-Adeno). In cases where CRPCs become indifferent to androgen receptor signaling, neuroendocrine PCa (CRPC-NE) may manifest, and ADT will no longer be effective [292]. Interestingly, significant upregulation of the SMARCA4 transcript and protein, along with SMARCA2 downregulation, has been observed in CRPC-NE compared with CRPC-Adeno [293]. The interrogation of RNA-seq data from multiple cohorts further confirmed that a low *SMARCA4* knockdown signature score correlates with a more aggressive PCa phenotype, including CRPC-NE and higher metastasis frequency [293]. The differential expression of SWI/SNF subunits between CRPC-Adeno and CRPC-NE led to the discovery of lineage-specific interactions, which are functionally important for cancer progression [293]. In CRPC, SMARCC2 dependency is attributed to antiandrogen resistance, with SMARCC2 and BRG1 acquiring new binding regions enriched for proto-oncogenes such as MYC, MAPK1, and ETS transcription factors [294]. In addition, AR-FOXA1-driven prostate cancer cells exhibit high dependence on the SWI/SNF complex, which makes them vulnerable to SWI/SNF ATPase degradation [295]. Complete inhibition of SWI/SNF ATPase activity reduces chromatin accessibility at core enhancers (AR/FOXA1/ERG/MYC), thereby mitigating oncogenic transcriptional programs [295]. Collectively, these data highlight the feasibility of targeting SWI/SNF complexes as an alternative therapeutic strategy in PCa.

Dysregulation of CHD family subunits is also linked to antiandrogen resistance. *CHD1* encodes the E-cadherin protein, which, when overexpressed with β-catenin at the membrane, promotes bone metastasis in prostate cancer cells [296]. CHD1 can function as either a tumor suppressor or an oncogene in prostate cancer, depending on the existence of different genetic events. In CHD1-deficient prostate cancer cells, the altered, plastic chromatin landscape upregulates multiple transcription factors (GR, NR2F1, TBX2, and BRN2), ultimately resulting in loss of luminal lineage identity and reduced AR dependency. AR inhibitor sensitivity is restored upon individual depletion of these transcription factors [297]. In PTEN-deficient prostate cancer, CDH1 acts through IL6 to remodel the tumor microenvironment via the recruitment of immunosuppressive MDSCs and inhibition of CD8 + T cells [188]. In accordance with this finding, a later study shows that deep deletion of *CHD1* is strongly associated with dendritic cell marker expression and an immunogenic phenotype [298]. Thus, the CHD1-IL6-MDSC regulatory axis may represent a key mechanism that drives immunogenicity in CHD1-deficient prostate cancers.

Integrative genomic profiling revealed CHD6 as the only CHD family member with elevated transcript expression in prostate cancer, correlating with poor prognosis [299]. CHD6 sustains cancer cell growth and invasion in an E2F1-dependent manner, and its chromatin binding is required for nucleosome eviction from promoters and gene bodies to maintain housekeeping transcriptional programs [299, 300]. On the other hand, due to promoter-associated hypermethylation, CHD8 is commonly downregulated in > 45% of primary PCa compared with benign tissues [301]. However, when CHD8 is overexpressed, it is associated with an elevated risk of biochemical recurrence and worse outcomes [301]. CHD8 promotes androgen-stimulated proliferation, interacts directly with the androgen receptor, and binds to the enhancer region of TMPRSS2 post androgen treatment [302]. As a cofactor for CTCF, CHD8 is required for CTCF-dependent insulation and epigenetic activity. For instance, loss of CHD8 induces CpG hypermethylation and histone hypoacetylation at CTCF binding sites near the inactive chromatin of *BRCA1* and *c-MYC* [303]. The function of the CTCF-CH8 complex is antagonized by its paralogue, BORIS, which competes for similar binding sites [301]. In PCa, an increased BORIS/CTCF ratio may work independently or in concert with the loss of CHD8 to further disrupt CTCF function and promote cancer progression [301].

RAD21, on the other hand, does not show significant differential expression between tumor and adjacent healthy tissue. Nevertheless, overexpressed RAD21 is associated with worse prognosis [82]. RAD21 exerts an antiapoptotic role against T-ERG-induced DMA damage and apoptosis, independent of PTEN status [82]. Although no RAD21 inhibitor is currently available, these findings suggest promising therapeutic strategies targeting RAD21-related vulnerabilities.

#### Cervical cancer

Cervical cancer is the 4th most common cancer and the 2nd leading cause of cancer mortality in females [242]. Definitive chemoradiotherapy (dCRT) is the standard treatment for locally advanced cervical cancer. However, *ARID1A* mutation is negatively associated with dCRT treatment outcomes [304]. ARID1A loss is frequent in cervical cancers (~ 30%) [305]. In fact, ARID1A protein expression declines gradually during the transition from normal cervical epithelium to carcinoma [306]. Patients with both *ARID1A* and *B2M* mutations exhibit an immunosuppressive environment characterized by reduced immune cell infiltration and impaired antigen presentation [304]. This downregulation of immune surveillance signatures may explain the poor response to dCRT and offers a rationale for personalized treatment in this subpopulation. YAP signaling represents a potential vulnerability in *ARID1A-*mutant cervical cancers. Chang’s work displayed that ARID1A inhibits the proto-oncogenic transcriptional coactivators YAP/TAZ by mediating their association with the SWI/SNF complex rather than with the DNA-binding platform TEAD [307].

Another significantly altered SWI/SNF complex member in cervical cancer is ACTL6A (BAF53). *ACTL6A* amplification is reported in 50% of cervical squamous cell carcinomas; its protein expression markedly increases from low-grade cervical intraepithelial neoplasia (CIN) to high-grade CIN and cancer [308]. This elevation may be partially attributed to downregulation of its suppressor miRNA (MiR-2161-3p) [309]. Functionally, ACTL6A drives cervical cell proliferation and cell cycle progression in a c-MYC dependent manner [308]. Silencing ACTL6A, in turn, exerts antitumor activity by promoting YAP phosphorylation and subsequently suppressing YAP/TAZ-mediated transcriptional activity [309]. Intriguingly, ACTL6A is indispensable for viral oncoproteins E6/E7 expression, as it establishes a chromatin structure that activates the p105 (or p97) promoter of HPV integrants [310]. This suggests that elevated ACTL6A is involved in HPV-driven oncogenesis, making it a promising therapeutic target.

Speaking of the HPV viral oncoproteins, E6 and E7, they regulate the transcription of multiple genes (e.g., PHF6, SF3B1, EIF4G3, DCP2) via the MBD2/MBD3 NuRD complex, thereby promoting cancer progression [311]. The binding of the E7 zinc finger to the NuRD complex via CHD4 inhibits HDAC from binding to the E2F2 promoter. This activates E2F2 transcription, facilitating HPV replication [312]. The cohesin complex also contributes to HPV replication and episome maintenance. In HPV-infected cells, constitutively activated SMC1 is recruited to viral genomes via association with CTCF, thereby supporting differentiation-dependent genome amplification [313]. During the early stage of the HPV life cycle, the viral genome recruits CTCF and YY1 to form an epigenetically repressed chromatin loop between the viral transcriptional enhancer and the early gene promoter. This attenuates E6 and E7 expression and likely prevents immune activation [314]. As cells differentiate, YY1 expression declines, resulting in the release of the repressive chromatin loop, derepression of the early promoter, and, eventually, elevated E6/E7 expression [314]. Despite high INO80 expression levels in cervical cancer patients and cell line models [315], its functions in cervical malignancies have not been investigated extensively. INO80 silencing suppresses cervical cancer cell proliferation and induces cell cycle arrest without affecting apoptosis or invasiveness [315]. This pro-proliferative effect may be mediated by INO80 binding to the NANOG transcription start site and promoting its expression [315]. Yeast two-hybrid (Y2H) experiments have suggested that INO80B interacts with HPV16 E7 and HPV5 E7, implying that HPV viral proteins modulate INO80 complex function [316]. Similar to ACTL6A, RAD21 increases in severity with cervical lesions. This aberrantly high RAD21 level positively correlates with XPO1 expression, suggesting possible coordination in the export of tumor suppressor proteins [317]. Polymorphisms of RAD21 double the likelihood of developing cervical cancer. Meanwhile, its depletion impairs cervical cell proliferation and improves radiosensitivity [317].

Taken together, there is a complex interplay between the HPV virus and host chromatin remodeling complexes. ACTL6A, CHD4, and RAD21 represent potential therapeutic and prognostic targets in cervical cancer.

#### Endometrial cancer

By 2022, cancer of the uterine corpus (endometrial cancer) ranked as the 15th most common cancer worldwide, and the 6th most prevalent cancer among females [242]. Endometrial carcinomas are classified by histological type: endometrioid carcinoma (EEC), serous carcinoma, clear cell carcinoma, mixed carcinoma, undifferentiated carcinoma (UEC), carcinosarcoma, unusual types, and gastrointestinal mucinous type. Current treatment strategies include surgery for stage I disease, surgery with or without radiation for stage II, and surgery with chemotherapy with or without radiation for stage III [318]. Frequently mutated chromatin remodeling genes in endometrial carcinomas include *ARID1A, SMARCA4, CHD4,* and *EZH2* [319].

Common genomic inactivation of SWI/SNF complex components has been identified in endometrial carcinomas, with the highest rates observed in aggressive UEC [320–322]. ARID1A is the most commonly mutated SWI/SNF component, occurring in about 40% of cases [323]. A meta-analysis has demonstrated that ARID1A loss represents an early event in endometrial cancer and serves as a specific marker for coexistent cancer [324]. Functionally, ARID1A inactivation accelerates endometrial tumor progression by impairing cellular responses to inhibitory TGF signaling [325]. Recent work by Jessica et al*.* revealed that endometrial tumors with ARID1A/B dual loss exhibit increased residual ncBAF and PBAF complex components, a shift in complex assembly that is reversed upon ARID1A restoration and accompanied by marked reductions in endometrial cell proliferation [323]. These findings indicate altered SWI/SNF complex dependence and suggest potential vulnerabilities for targeting ncBAF and PBAF. Indeed, genetic- and small molecule-mediated disruption of ncBAF or PBAF weakens oncogenic chromatin regulation and diminishes endometrial tumor growth [323]. Loss of SMARCA4, SMARCA2, and SMARCB1 in uterine carcinomas is less frequent [326–328]. Karnezis et al*.* found that loss of SMARCA4 and SMARCB1 proteins in the undifferentiated component of dedifferentiated endometrial carcinoma is mutually exclusive [328]. Interestingly, all SMARCA4- or SMARCB1-deficient tumors showed concomitant SMARCA2 loss, suggesting that such co-inactivation may drive progression from low-grade endometrioid carcinoma to undifferentiated carcinoma [328].

Unlike the SWI/SNF complex, the TCGA UCEC data suggest low mutation rates in INO80 complex genes [329]. However, the INO80 complex has been implicated in endometrial carcinoma through its association with aryl hydrocarbon receptor nuclear translocator-like (ARNTL). Clinically, high ARNTL expression positively correlates with poor tumor differentiation, advanced tumor stage, and metastasis [330]. ARNTL directs endometrial carcinoma metastasis and immunosuppression through INO80 and its downstream target DHX15 [330].

Somatic mutation of *CHD4* occurs in 17% of endometrial carcinoma, though its functional roles remain incompletely characterized [331]. *CHD4* mutations reduce CHD4 protein stability and promote cancer progression via activation of multiple tumorigenic pathways, including TGFB1, EGFR, MYC, P53, and mTOR signaling pathways [332, 333]. EZH2 has been reported as a prognostic marker [334]. Specifically, high EZH2 levels are associated with aggressive phenotypes [335], likely by repressing the tumor-suppressive miR-361 through a YY1-dependent mechanism [336]. Recent publications have also shown that cohesin-mediated 3D genome organization is associated with focal silencing of the potent tumor suppressor, Paired box gene 2 (*PAX2*) in endometrial cancer [337]. Focused investigations are warranted to elucidate the dysregulated epigenetic mechanisms underlying endometrial carcinogenesis.

#### Breast cancer

Breast cancer is the most prevalent cancer in women globally and is classified into four subtypes based on ER, PR, HER2, and Ki-67 expression. Most studies on chromatin remodeling in breast cancer have centered on the SWI/SNF complex. ARID1A, a subunit of cBAF complex, is frequently mutated in ER-positive breast cancer, with a mutation rate of 4–6%. Interestingly, ARID1A mutations are acquired during hormone treatment and are enriched in treatment-resistant and metastatic cases, with a mutation rate of 12% [338]. Meanwhile, an ARID1A-inactivating mutation regulates ER-FOXA1 chromatin interactions by decreasing SWI/SNF binding to ER regulatory regions, thereby switching the cell from luminal to basal-like [339]. In contrast, ARID1B is highly expressed in triple-negative breast cancer (TNBC) and linked to poorer outcomes. ARID1B accumulation enhances tumor survival by negatively regulating ARID1A and epigenetically controlling ZNF382 expression [340]. SMARCA4, the catalytic subunit of the SWI/SNF complex, functions as a tumor suppressor in breast cancer. It is mutated in 1.8% of cases across all subtypes, and reduced expression is even more frequent, regardless of the presence of mutations [341]. Experiments on 3D tumor spheroids showed that SMARCA4 loss activated RHOA signaling and disrupted cell adhesion, thereby promoting metastasis in vivo. Additionally, SMARCA4 directly interacts with BRCA1, mediating its coactivation function and playing an important role in breast cancer [342]. BRD7, a PBAF-specific subunit, also influences BRCA1 function by facilitating its recruitment to the ESR1 promoter [343]. BRD7 is also essential in maintaining lung metastatic dormancy; Its loss reprograms the tumor immune microenvironment and reactivates the metastasis [344].

RAD21, a core subunit of cohesin, is elevated in 13% of breast cancer cases according to TCGA studies [345]. Enhanced RAD21 expression is associated with poor prognosis in certain breast cancer populations, such as high-grade breast cancer and those with BRCA2 and BRCAX mutations [346, 347]. Overexpression of RAD21 may induce EMT, though the underlying mechanisms by which cohesin promotes breast cancer progression are still unclear [348].

### Urinary system

#### Kidney cancer

Renal cancer (RC) exhibits distinct geographic and gender-based variations in incidence [349]. Comprehensive genomic profiling of 169 advanced papillary renal cell carcinoma (RCC) cases revealed significant enrichment of SWI/SNF complex alterations in papillary renal cell carcinoma (PRCC) type 2. This distinguishes type 2 from type 1 and is correlated with disease progression not observed in TCGA early-stage cohorts [350].

PBRM1 (also known as BAF180) is the second most common mutated tumor suppressor in clear cell renal carcinoma (ccRCC), occurring in approximately 41% of patients after VHL [351]. Whole-exome sequencing of metastatic ccRCC cohorts revealed that *PBRM1* LOF mutations significantly correlate with immune checkpoint blockade efficacy and are accompanied by enhanced JAK-STAT, hypoxia, and IFN-γ immune signaling programs [352]. However, a pan-cancer analysis reported that *PBAF* mutations are most frequent in ccRCC (*PBRM1* 7.4%, *ARID2* 6.5%) but are not associated with ICB efficacy; instead, *PBRM1* mutations correlate with elevated angiogenesis gene expression, suggesting greater suitability for anti-VEGF therapy rather than immunotherapy [353]. In *VHL*^−/−^ ccRCC, PBRM1 loss amplifies HIF-driven transcription via BRG1-dependent PBAF activity. By weakening the BRD7-SMARCA4 association, it redirects residual PBAF to aberrant loci, thereby recruiting NF-κB and activating pro-survival genes [241, 354].

Renal medullary carcinoma (RMC) is a highly lethal malignancy driven by *SMARCB1* mutations, primarily affecting young patients with sickle cell trait and individuals of African descent [355]. It typically presents with metastases and carries a dismal prognosis [356]. In a retrospective study of 20 RMC patients, FISH analysis revealed concurrent hemizygous loss and translocation of SMARCB1 in 11 cases (55%), homozygous loss in 6 cases (30%), and 3 cases (15%) with no structural or copy-number alterations despite protein loss [357]. Biallelic SMARCB1 loss drives RMC tumorigenesis by repressing renal epithelial transcription factors, including TFCP2L1, HOXB9, and MITF, thereby activating MYC- and NFE2L2-associated oncogenic programs and ferroptosis resistance, while also activating the cGAS-STING pathway to enhance tumor immunogenicity [358, 359]. DPF3, an SWI/SNF complex subunit, underlies the 14q24 susceptibility locus through risk-allele-driven overexpression, contributing to inhibition of apoptosis, activation of STAT3 oncogenic pathways, and alteration of chromatin accessibility [360]. Its short isoform, DPF3a, further promotes ccRCC metastasis through TGF-β signaling activation [361].

### Respiratory system

#### Lung cancer

Lung cancer remains the leading cause of cancer-related death worldwide. In a cohort of 2,689 non-small cell lung cancer (NSCLC) patients, SWI/SNF complex mutations in *ARID1A/B*, *ARID2*, *PBRM1*, *SMARCA4*, or *SMARCB1* were identified in 20.6% of cases (n = 555); these tumors exhibited fewer targetable driver alterations, higher tumor mutational burden, and inferior survival, with *SMARCA4* mutations conferring the worst prognosis [362]. SMARCA4 expression is markedly elevated in Small Cell Lung Cancer (SCLC) lines compared to any other solid tumor represented in the Cancer Cell Line Encyclopedia (CCLE) [363]. Loss of both BRG1 and BRM expression occurs in 10% of lung cancers and is significantly associated with poorer patient survival [364]. Moreover, among 1199 smokers in a case–control study, homozygous BRM promoter insertion variants (*BRM − 741* and *BRM − 1321*) were associated with increased lung cancer risk [365] and predicted worse overall and progression-free survival in advanced NSCLC [366]. Loss of BRG1 deregulates multiple oncogenic signaling pathways, including MYC target gene sets, neuroendocrine transcriptional programs, energy stress response, cell cycle progression, and genomic stability pathways [179, 367–371]. BRG1 exhibits dual roles in cancer; its loss sensitizes cells to malignant transformation and tumor progression in a cell-type-dependent manner [367]. In NSCLC, BRG1 depletion reduces cyclin D1 expression and upregulates OXPHOS pathway genes. By impairing the transcriptional response to energy stress, it promotes lung cancer development [179] [370]. BRG1 may also drive tumorigenesis by increasing genomic instability through replicative stress [371]. In SCLC, *SMARCA4* mutations promote progression by inhibiting neuroendocrine (NE) transcription factors (ASCL1, NEUROD1) and inducing REST alternative splicing via SRRM4, thereby driving a subtype transition from high-NE to low-NE states and conferring chemotherapy resistance [363]. Conversely, BRG1 loss in MAX-deficient SCLC creates synthetic lethality by disrupting BRG1-mediated activation of MAX-dependent neuroendocrine programs and MYC target genes (e.g., glycolysis regulators), thereby impairing cancer cell proliferation and survival [369]. SMARCA4 inactivation also grants resistance to retinoic acid and glucocorticoids by antagonizing MYC activity via direct binding to MYC and MYC-target promoters, a functional opposition similarly observed in primary tumors [368]. Mutations in *BRG1* or *BRM* impair NRSF/REST-mediated repression of neuronal genes in NSCLC cell lines and enhance tumorigenicity [372]. This functional redundancy is underscored by reciprocal interactions between BRG1 and BRM. The two paralogs exhibit mutual dependence: expression of either SMARCA2 or BRG1 completely rescues the effects of SMARCA2 knockdown in BRG1-deficient cells [373, 374]. This phenomenon, termed "cancer-selective paralog dependency," occurs when the loss of one family member reveals critical dependence on the remaining paralogous subunits [375]. Indeed, BRM depletion in BRG1-deficient cancer cells induces cell cycle arrest, senescence, and increased global H3K9me3 levels [375]. Previous studies have shown that SMARCA4 loss impairs the function of all three classes of SWI/SNF complexes [367].

The functional complementation of SMARCA2 and SMARC4 is also evident at the transcriptional level, where their ATPase activity appears to regulate gene programs related to proliferation, the cell cycle, and chromatin remodeling [374]. Consequently, the simultaneous loss of both paralogs is synthetically lethal during tumorigenesis [376]. SMARCA4/2-deficient cancer cells exhibit reduced expression of the ER-Ca2 + channel IP3R3, diminishing calcium transfer to mitochondria and promoting chemoresistance through apoptosis suppression [377]. Epigenetic reprogramming in *SMARCA4*-mutant cancer cells reduces immune infiltration and immunotherapy efficacy by downregulating immunostimulatory genes, a vulnerability that may be targeted to overcome immunotherapy resistance [378]. SMARCA4 chromatin remodeling activity also controls Osimertinib-induced oxidative stress levels in resistant cells via NRF2 activation. Some Osimertinib-resistant tumors evolve during treatment such that their survival becomes dependent on SMARCA4, demonstrating that SMARCA4 can exert pro-growth functions in addition to its well-established tumor-suppressor role [379]. SMARCE1 loss induces EGFR expression through regulating the levels of a key element of the Polycomb Repressive Complex, Chromobox Homolog 2 (*CBX2*) [380]. ARID1A loss increases chromatin accessibility, enhancing HIF1α/BRD4-mediated transcriptional activation of glycolysis genes (*PGAM1*, *PKM*, *PGK1*) [381]. Upregulated INO80 promotes an open chromatin state and enhanced genome accessibility, activating oncogenic transcription to drive tumorigenesis [75]. RAD21 amplification drives LUAD proliferation and metastasis by epigenetically regulating H3K4me3 and MAPK/ERK pathways, inducing widespread transcriptional dysregulation of cancer-associated genes [382].

### Hematologic system

#### Lymphoma

Lymphoma can be broadly categorized into two categories, namely Hodgkin lymphoma (HL) and non-Hodgkin lymphoma (NHL). NHL is further divided into more than 90 distinct subtypes based on their cell type origin (B, T, or NK cells) and clinical behavior (indolent or aggressive) [383]. NHL is one of the most prevalent cancers, ranking 10th by global incidence, whereas HL is rare, placing 26th [242].

Among germinal center (GC) B cell lymphomas, frequent mutations in SWI/SNF components, particularly *ARID1A* and *SMARCA4*, have been observed in Burkitt’s lymphoma [384]. *ARID1A* mutations have been documented in approximately 20% of lymphomas [148, 385–387]. *ARID1A* haploinsufficiency skews GC cell fate toward an immature IgM^+^CD80^−^PDL2^−^ memory B cell program, which is capable of reinitiating GCs and promoting subsequent mutagenesis [148]. When combined with BCL2 deregulation, *ARID1A* haploinsufficiency accelerates the transformation of follicular lymphoma (FL) into aggressive diffuse large B-cell lymphoma (DLBCL) [148]. H1 allele mutation, occurring in ~ 30% of cases, also drives aggressive DLBCL-like lymphomas via chromatin decompaction [388].

*SMARCA4* inactivating mutations occur in 3–34% of lymphomas, with the highest frequency observed in pediatric Burkitt lymphoma [389]. The presence of *SMARCA4* mutations is associated with poor progression-free survival [390]. In GC B cells, biallelic loss of *SMARCA4* is lethal, whereas partial loss favors centrocyte recycling back to the dark zone over plasma cell differentiation. Within the dark zone, centroblasts proliferate rapidly, leading to GC hyperplasia [389]. Combined *MYC* conditional knock-in and *SMARCA4* conditional knockout significantly expands GC B cells and shortens survival in mice, implying that *SMARCA4* serves as a haploinsufficient tumor suppressor in GC B cells [389].

*SMARCA2* alterations in lymphoma are less frequent (< 5%) [391]. In mantle cell lymphoma (MCL), *SMARCA2* deletion or low expression is associated with disease progression and poorer survival outcomes [390]. A preliminary study by Yan et al*.* demonstrated that SMARCA2 downregulation promotes cell proliferation, increases resistance to Ibrutinib (a Bruton tyrosine kinase inhibitor), upregulates ROS levels, and reduces accessibility at intronic regions of *TENT5C* [392]. TENT5C has been suggested to be a B-cell lineage-specific growth suppressor, particularly in multiple myeloma [393]. Importantly, overexpression of TENT5C in SMARCA2-deficient MCL cell lines restores Ibrutinib sensitivity [393]. Although the roles of SMARCA2 in lymphoma development are yet to be studied extensively, SMARCA2 status appears critical in determining drug response trajectory.

Mutations in cohesin complex genes, including *SMC3, RAD21, and STAG2*, have been reported in approximately 5% of DLBCL. In murine models, *SMC3* haploinsufficiency (*Smc3*^wt/−^) results in GC hyperplasia, centrocyte expansion, enhanced proliferation in GC B cells, and impaired plasma cell differentiation [394]. Mechanistically, *SMC3* haploinsufficiency expedites lymphomagenesis by reducing enhancer connectivity at tumor suppressor genes through constitutive Bcl6 expression [394]. Consistent with these preclinical data, *SMC3* haploinsufficiency is associated with worse clinical outcomes and reduced expression of cohesin core subunits (*SMC3, SMC1A, RAD21, STAG1, STAG2)* in GC-derived-DLBCL patients [395]. Pan-cancer analysis has further revealed that DLBCL exhibits upregulated mRNA expression of *CHD4, CHD8,* and *CHD9* [396]. The CHD4-NuRD complex is essential for lymphoid cell fate, as it regulates B-cell development and lineage progression [397, 398].

#### Leukemia

Leukemia is the 13th most common cancer and the 10th leading cause of cancer-associated mortality [242]. Advances in understanding the complex 3D chromatin landscapes in leukemia have uncovered potential therapeutic vulnerabilities [399, 400]. Yet, alterations in core SWI/SNF ATPases, such as SMARCA4, are uncommon in leukemia compared to solid tumors [401]. SMARCA4 (BRG1) overexpression is associated with worse clinical outcomes in leukemias [402]. Mechanistically, SMARCA4 is essential for leukemia cell proliferation but dispensable for long-term repopulating hematopoietic stem cells (LT-HSCs) [403]. By occupying the transcriptional activation site of PPP2R1A, SMARCA4 inhibits PPP2R1A expression in B-cell acute lymphoblastic leukemia (B-ALL). This subsequently activates the PI3K/AKT signaling, driving B-ALL cell proliferation [402]. Notably, despite its antiapoptotic roles in B-ALL, high SMARCA4 expression has been identified as a sensitivity marker for topoisomerase II inhibitors in acute myeloid leukemia, correlating with substantially improved patient survival [404]. In contrast to its expression pattern in solid tumors, SMARCA2 is significantly overexpressed in acute myeloid leukemia (AML) compared to normal hematopoietic cells [405]. In the presence of FLT3-gain-of-function internal tandem duplication (FLT3-ITD) mutation, both SMARCA4 and SMARCA2 expression are elevated [405].

*ARID1A* mutations are rare in leukemia (< 10%) [406, 407]. Intriguingly, recurrent *ARID1A* mutations are acquired upon AML relapse and clones harboring these mutations expand during relapse, driving the disease progression via aberrant chromatin modification [408]. In concordance with this finding, ARID1A expression is lowest in relapsed AML, followed by newly diagnosed AML, and highest in individuals achieving complete remission [409]. Functionally, shRNA-mediated knockdown of *ARID1A* suppresses apoptosis via the TGF-β1/SMAD3 pathway [409]. In mouse models, ATAC-seq revealed that ARID1A loss induces a global reduction in open chromatin at regions critical for hematopoiesis and impairs myeloid and erythroid differentiation [410].

Clinically, low expression of CHD3, CHD4, CHD5, or CHD7 correlates with improved survival in leukemia [396]. Consistent with the clinical findings, using CRISPR-Cas9 approaches, CHD4 has been shown to be crucial for leukemic cell growth, and its disruption results in cell cycle arrest and MYC downregulation [411]. Moreover, CHD4 depletion sensitizes AML cells to DNA-damaging agents and impairs their ability to form tumors, highlighting CHD4 as a potential therapeutic target in AML [412]. In contrast, CHD7 acts as a differentiation-promoting brake by genetically interacting with RUNX1 to regulate hematopoietic stem and progenitor cell expansion [413, 414]. Recent CRISPR-Cas9 knockout screens and multi-omics analyses further identified that CHD7 confers multidrug resistance by activating P13K/AKT and MAPK/ERK signaling in AML [415].

Within the ETV6:RUNX1 B‑ALL subtype, *INO80* genetic alterations are associated with increased relapse risk, with higher frequencies of deletions and sequence mutations reported in relapsed cases (~ 15%) compared to those in complete continuous remission (~ 7%) [416]. Although the expression of ACTR5 and its protein product, ARP5, in leukemia is not well documented, detectable ARP5 has been reported in AML cell lines [417]. ACTR5 is functionally important: its depletion in AML cells compromises cell viability, derepresses the tumor suppressor CDKN2A, and inactivates the CDK6/p-Rb/E2F1 axis [417]. These findings imply that targeting ACTR5 in combination with CDK4/6 inhibitors could be further exploited in AML.

Cohesin gene mutations in AML are recurrent genetic events, with frequency ranging between 6–13% [418–420]. In a retrospective multicenter cohort study, mutations in cohesin genes (*STAG2*, *RAD21, SMC1A*, *SMC3*) were nearly mutually exclusive [418]. Notably, none of these alterations showed a significant association with clinical outcomes [418]. STAG2*-*deficient AMLs exhibit increased STAG1 chromatin occupancy, suggesting functional compensation between STAG homologs [421]. However, STAG2 stabilizes cohesin at CTCF-independent regulatory elements, whereas STAG1 retains cohesin at CTCF-dependent sites [421]. Therefore, STAG1 compensation in STAG2-deficient AML cells leads to altered 3D chromatin structure and weakened enhancer-promoter contacts that are critical for leukemic transformation [421].

### Nervous system

#### Brain cancer

Gliomas are among the most common and aggressive primary brain malignancies, propagated by stem-like cancer cells refractory to existing therapies [422, 423]. *H3K27M* mutations reprogram SWI/SNF complexes (BAF/PBAF) to promote glioma progression by upregulating SMARCA4, SMARCA2, and PBRM1 expression, thereby sustaining the oncogenic phenotype [424]. The BRG1-BAF complex maintains a progenitor state in H3K27M gliomas by binding to promoters and enhancers of genes involved in cell growth and ECM, and thus preventing glial differentiation [425, 426]. In atypical teratoid rhabdoid tumors (ATRT), biallelic SMARCB1 loss reshapes chromatin to suppress differentiation programs and enhance Polycomb-mediated repression, driving tumorigenesis [427]. *SMARCC2* LOF mutations promote glioblastoma proliferation by derepressing EGR1-mediated DKK1 transcription through impaired chromatin remodeling, leading to PI3K-AKT pathway activation [428].

Ultimately, these findings position SWI/SNF chromatin remodelers as central epigenetic dependencies in glioma and highlight their subunits as potentially attractive therapeutic targets in glioblastoma.

### Integumentary system

#### Skin cancer

Malignant melanoma exhibits notorious resistance to conventional therapies and is characterized by both genetic and epigenetic alterations [429]. Many efforts have been employed to develop novel therapies for this deadly disease [430–432]. Unlike genetic variations, epigenetic alterations are more readily reversible, offering significant opportunities for cancer therapy [433]. BRG1 expression is lost early in melanoma, with nearly 70% of primary tumors lacking detectable BRG1 expression, while BRM expression is usually maintained (< 20% loss) [434]. However, in Nonmelanoma skin cancer patients, BRM protein loss may result from reduced mRNA levels, whereas BRG1 protein loss appears to occur post-translationally [435]. Conversely, other studies have reported that BRG1 is significantly upregulated during melanoma progression and is involved in melanoma initiation [436, 437]. Reduced SMARCB1 (SNF5) expression is also significantly associated with melanoma progression and poorer patient survival [438]. BRG1 modulates the expression of ECM-related genes, particularly upregulating E-cadherin while downregulating MMP1 and integrins α4 and β3 [437].

PHF10 interacts with MYC and recruits the PBAF complex to target gene promoters, thereby enhancing MYC-driven transcriptional activation of cell-cycle progression genes [439]. ARID2 loss inhibits REST binding and inactivates its targets, leading to upregulation of synaptic transcripts [440]. Loss of STAG2 inhibits CTCF-mediated expression of dual specificity phosphatase 6 (DUSP6), thereby reactivating mitogen-activated protein kinase (MAPK) signaling and exerting resistance to BRAF inhibitors [441]. Additionally, its loss activates IRF9 by reconfiguring 3D genome organization, which enhances type I interferon signaling and upregulates PD-L1 expression, ultimately promoting tumor formation and progression [189].

Loss of PBAF function, including the PBRM1 and ARID2 subunits, increases tumor cell sensitivity to interferon-γ, enhancing chemokine secretion to recruit effector T cells, and ultimately results in treatment-resistant tumors responsive to immunotherapy [442]. ARID1A loss disrupts chromatin remodeling, driving genomic instability and mutational burden that generate neoantigens, thereby boosting sensitivity to immunotherapy (e.g., PD-1 inhibitors) in previously resistant tumors [443].

### Endocrine system

#### Thyroid cancer

Thyroid cancer is the most common endocrine malignancy and generally carries a favorable prognosis for most patients. However, differentiation status critically determines therapeutic response and clinical outcome [444]. Next-generation sequencing of 341 cancer genes in 117 patient-derived poorly differentiated thyroid cancers (PDTCs) and anaplastic thyroid cancers (ATCs) revealed that mutations in SWI/SNF chromatin remodeling complex components occurred in 36% of ATCs and 6% of PDTCs [445].

Genes encoding SWI/SNF complex subunits were mutated in 9 out of 57 patients (16%), with mutations identified in *ARID1B* (9%), *ARID2* (7%), *SMARCB1* (4%), and *PBRM1* (1.8%) [446]. These mutations exhibited mutual exclusivity, implying that alteration of a single subunit is sufficient to perturb SWI/SNF function [446].

In thyroid cancer cell lines, genetic alterations in SWI/SNF members, such as *ARID1A*, *ARID1B*, *ARID2*, *SMARCA4*, *SMARCD1*, *PBRM1*, and *ATRX,* were identified in 18 of 58 lines (31%) [447]. Functionally, SWI/SNF loss downregulates thyroid terminal differentiation genes and reduces chromatin accessibility at key thyroid lineage transcription factor loci, ultimately promoting stem cell-like properties and tissue regeneration [448].

### Connective tissue system

#### Sarcoma

Epithelioid sarcoma is a rare soft tissue neoplasm of uncertain lineage that typically arises in the distal extremities of adults, presents with frequent recurrences and metastases, and often poses diagnostic dilemmas [449]. Nearly 50% of epithelioid sarcoma cases exhibit *SMARCB1* (*INI1*) deletion at both the genomic and protein levels [449]. Deficiency of Srg3, a mouse homolog of BAF155, promotes G1 cell-cycle arrest and upregulates p53 and p21 expression [450].

Synovial sarcoma is characterized by the fusion of SS18 to one of the *SSX* genes. SS18 is a stable and integral component of SWI/SNF complexes [451]. The SS18-SSX2 fusion protein induces transcriptional reprogramming through epigenetic dysregulation, disrupting canonical SWI/SNF architecture and function, and leading to dysregulation of downstream targets involved in cholesterol synthesis and IGF signaling [452, 453]. Furthermore, SS18-SSX redirects BAF47 away from enhancers to activate broad polycomb-repressed domains, driving oncogenic activation of bivalent genes [454]. In Rhabdomyosarcoma (RMS), sustained BAF53a expression interferes with cellular differentiation [455].

In Ewing sarcomas driven by the EWS-FLI1 fusion oncogene, *RAD21* copy gain mitigates replication stress and promotes tumorigenesis [456]. *STAG2* (*SA2*) LOF mutations enhance migration and invasion by altering CTCF-anchored loop extrusion and reducing cis-mediated EWSR1-FLI1 activity [457]. Moreover, its loss disrupts PRC2-mediated gene regulation, reprogramming migratory and neurodevelopmental transcriptional programs [174]. In SA2-deficient Ewing sarcoma, SA1 serves as a putative synthetic-essential target; its inhibition leads to mitotic defects and impaired DNA damage repair [458].

### Summarized functional roles of remodeler subunits in cancers

Across diverse cancers, SWI/SNF alterations represent the most frequent chromatin-remodeling defects. Importantly, chromatin remodelers often exhibit context-dependent roles across cancer types, sometimes further affected by lineage, mutational context, and disease stage.

Specifically, ARID1A functions as a tumor suppressor in most cancers but shows dual behavior in gastric and liver cancer [192, 207–209, 221]. Similarly, ARID2 is generally a tumor suppressor, but its loss in skin cancer drives oncogenesis [218, 442]. PBRM1/BAF180 is broadly tumor suppressive except in brain tumors [239, 351, 424]. SMARCB1/INII is a well-established tumor suppressor across most cancers except liver cancer [196, 427]. For the SWI/SNF core ATPases, SMARCA4/BRG1 was initially proposed as a tumor suppressor, but later studies have indicated its oncogenic role in leukemia, prostate, and brain cancer [293, 402, 424]. In addition, SMARCA4 could play a dual role, both tumor-suppressing and oncogenic, in cancer formation [233]. SMARCA3/HLTF remains a tumor suppressor [268]. SMARCC2/BAF170, BRD7, and SMARCA1 are found to be tumor suppressive in brain tumors, breast cancer, and colorectal cancer, respectively [271, 344, 428].

On the other hand, several chromatin remodelers function primarily as oncogenes. ACTL6A/BAF53 is an oncogene in cervical, esophageal, and gastric cancer [213, 251, 308]. ARID1B acts as a suppressor in thyroid cancer. Conversely, it serves as an oncogene and negatively regulates ARID1A in TNBC [340]. SMARCA2/BRM is oncogenic in leukemia, pancreatic, and brain cancer [228, 405, 424], but suppressive in others [246, 293, 364]. Loss of ACTR5, a subunit of INO80, can be found in leukemia [417]. Beyond leukemia, the broader INO80 complex is often oncogenic [291]. SMARCA5/RSF1 and RAD21 are both oncogenic [82, 130, 285]. Another cohesion complex subunit, SMC1A, also acts as an oncogene in colorectal cancer [259]. Within the CHD family, CHD1 and CHD8 show dual roles in prostate cancer [301, 459]; CHD4 acts as a tumor suppressor in ovarian cancer [290]. But at the same time, CHD4 functions as an oncogene in leukemia, and DLBCL [396, 412]. CHD6 and CHD8 are oncogenic in prostate cancer and DLBCL, respectively [299, 396].

Overall, disruption of chromatin remodeling contributes to cancer via transcriptional dysregulation, DNA repair defects, altered 3D genome topology, immune evasion, and therapy resistance. However, the ambiguous functional roles of remodeler mutations under different contexts and LOF make them difficult to target directly, thus posing a major translational challenge. Current therapeutic development is shifting from direct inhibition towards synthetic lethality, epigenetic synergy, direct targeting of remodeler proteins, and cohesin deficiency, which will be discussed in detail in the following sections.

## Therapeutic strategies targeting chromatin remodeling

**Fig. 6:**
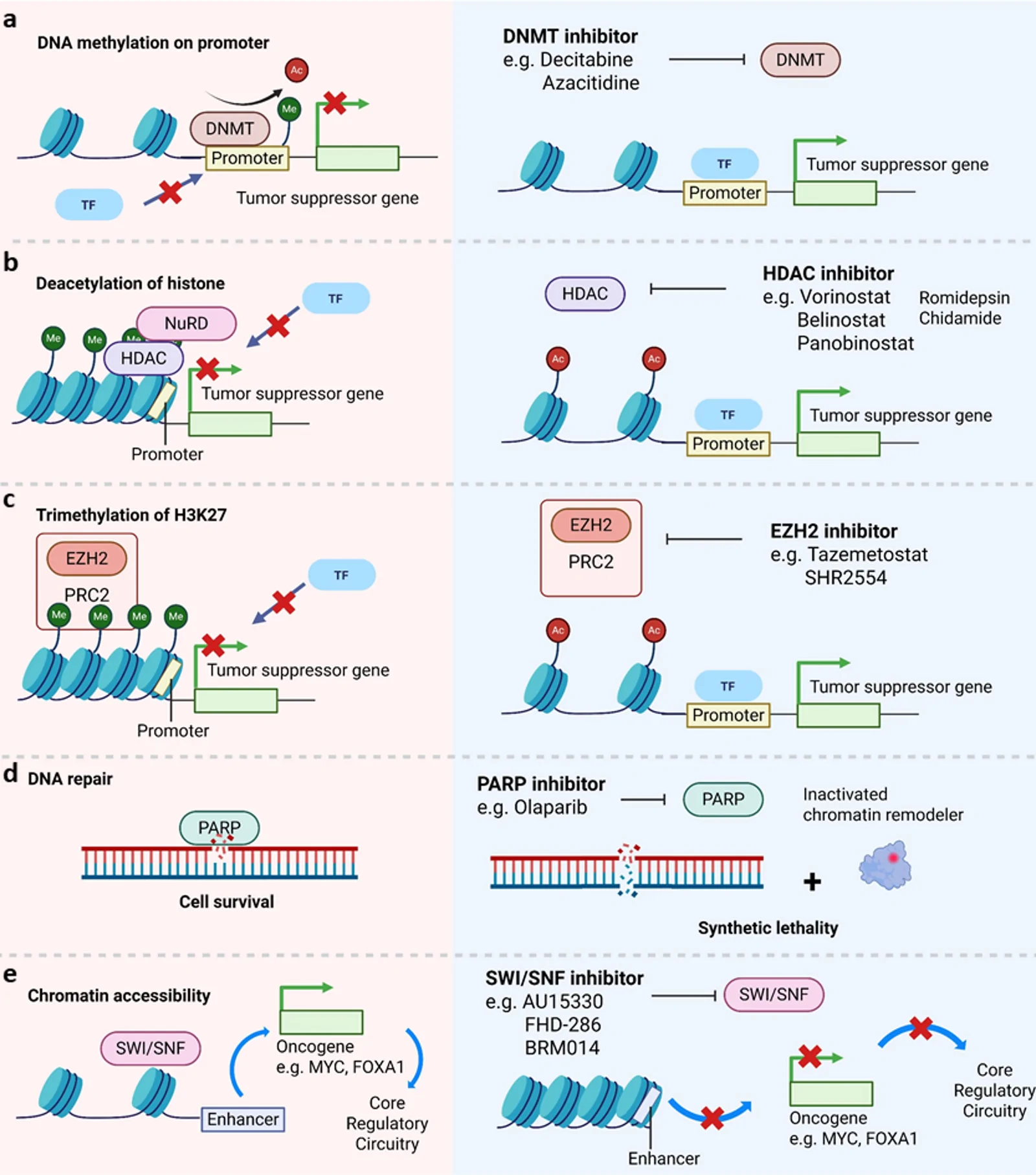
Current therapeutic strategies targeting chromatin remodeling. (Figure updated) Therapeutic strategies targeting aberrant chromatin remodeling in cancer. Aberrant chromatin remodeling primarily involves the misregulation of tumor suppressor genes (TSGs) and oncogenes. **a** When chromatin remodeling abnormalities lead to oncogene upregulation while DNMT-mediated promoter hypermethylation silences TSGs, the resulting imbalance promotes tumor proliferation. In this context, DNMT inhibitors can block promoter methylation of TSGs, allowing transcription factors to bind normally. **b** The chromatin remodeler CHD can form the NuRD complex with HDACs, which suppresses TSG expression through histone modification and nucleosome repositioning. HDAC inhibitors may reverse this repression. **c** EZH2, a core component of PRC2, interacts with both chromatin remodelers and cohesin, and mediates H3K27 trimethylation to promote chromatin compaction, thereby hindering the function of remodelers such as SWI/SNF. EZH2 inhibitors suppress EZH2 activity and help restore the balance between PRC2 and chromatin remodelers. **d** Furthermore, aberrant chromatin remodeling affects DNA repair. Since PARP is a key factor in homologous recombination, PARP inhibitors represent a promising strategy; in tumors harboring inactivating mutations in chromatin remodelers, PARP inhibitors can induce synthetic lethality. **e **Direct inhibitors targeting chromatin remodelers are not yet clinically approved, but SWI/SNF inhibitors currently under clinical trial can directly block SWI/SNF complexes to suppress oncogene expression further

Chromatin remodelers and cohesin subunits are frequently mutated or altered across diverse cancer types, thereby influencing tumor progression and therapeutic response. Consequently, treatment strategies targeting chromatin remodeling in cancers are rapidly developing. Although cancer-type specificities exist, common pan-cancer principles can still be summarized (Fig. 6).

### Therapeutic strategies targeting first-level chromatin remodeler deficiency

Therapeutic strategies targeting first-level chromatin remodeler deficiency mainly focus on the SWI/SNF complex based on its significant contribution in diverse cancers and can be generally categorized into four classes: synthetic lethality, epigenetic synergy, direct targeting of remodeler proteins, and modulation of other pathways.

#### Synthetic lethality

Within the large variety of remodeler complex subunits, ARID1A is one of the most extensively characterized synthetic lethal targets. ARID1A deficiency disrupts HR repair by downregulating DDR genes, thereby shifting reliance towards non-homologous end joining NHEJ [460]. Furthermore, ARID1A is involved in regulating the Akt/mTOR signaling axis. ARID1A-mutant tumors are therefore overwhelmingly sensitive to agents that target DNA repair and cell cycle checkpoints.

The G2/M checkpoint kinase WEE1, which is activated by the ATR-CHK1 cascade after DNA damage, displays a promising approach [461]. In a phase 1b/2 trial evaluating the WEE1 inhibitor SC0191 in metastatic colorectal cancer (NCT06363552), ARID1A/TP53 co-mutant tumors exhibited increased sensitivity, characterized by R-loop accumulation, exacerbated DNA damage, and cell death due to repair deficiencies [462]. PARP inhibitors (e.g., Olaparib) have shown efficacy in ARID1A-mutant pancreatic and breast cancers, and inhibition of ARID1B nuclear import further enhances this sensitivity in TNBC models [463]. This is because of the critical role of PARP in the NHEJ pathway and increased dependency of ARID1B in ARID1A-deficient contexts [464]. Two parallel nonrandomized phase II clinical trials further reported that Olaparib was safe and therapeutically efficacious in pre-treated PC patients with ARID1A mutations. In addition, the ATR inhibitor Ceralasertib is currently under investigation in ARID1A-deficient ovarian clear cell (NCT0405269), with preliminary results demonstrating clinical activity as a monotherapy and in combination with the PARP inhibitor Olaparib [277, 465].

Beyond DNA repair pathways, ARID1A loss disrupts iron and lipid homeostasis, enabling ferroptosis induction by c-MET inhibitors (e.g., PHA-665752, crizotinib, cabozantinib) in CRC [466]. Similarly, ARID1A-deficient-induced PKM suppression, which impairs glycolytic activity, drives TCA/OXPHOS dependence and sensitizes ARID1A-deficient HCC to cuproptosis [467]. Other SWI/SNF-mutant lung tumors, especially those with SMARCA4 mutations, also exhibit a therapeutic vulnerability to OXPHOS inhibitors, of which IACS-010759 demonstrated potent and selective antitumor efficacy [179].

Dysregulation of cell cycle checkpoints represents another synthetic lethal opportunity in SWI/SNF-mutant cancers. The p53 activator, RITA, promotes p21 downregulation while upregulating the pro-apoptotic genes PUMA and NOXA in ARID1A-deficient cells [468]. Concurrently, ARID1A depletion worsens RITA-induced DNA damage by downregulating CHK2 phosphorylation [468]. ARID1A loss shifts the balance towards EZH2-driven repression, and inhibition of EZH2 has presented a synthetic lethality in ovarian and colorectal cancers [469, 470]. Besides, overexpression of ABL, SRC, and c-KIT has been observed in ARID1A-deficient cancers, suggesting multi-kinase inhibitors (i.e. Dasatinib) may represent a viable therapeutic strategy following G1-S cell-cycle arrest [471]. Finally, Aurora kinase A (AURKA) inhibition selectively impairs ARID1A-deficient CRC growth by selectively inducing multinucleation, G2-M cell cycle arrest, and apoptosis through the AURKA-CDC25C axis [472].

Apart from ARID1A, synthetic lethal vulnerabilities have been identified across other components of chromatin remodelers. For instance, EZH2 overexpression is a hallmark of SMARCB1-loss tumors such as epithelioid sarcoma, extracranial, extrarenal malignant rhabdoid tumors, and synovial sarcoma. EZH2 inhibitors (i.e., FDA-approved Tazemetostat), and EZH1/2 dual inhibitors (i.e., valemetostat, and GSK126) demonstrated promising efficacy [473]. Besides, inhibition of histone deacetylase (HDAC) also displayed significant antitumor potential in SMARCB1-deficiency [474]. Increased dependencies on MDM2/4 have also been observed in SMARCB1-loss rhabdoid tumors, leading to the emergence of inhibitors such as ATSP-7401 or idasanutlin [475]. Cyclin B, which promotes the G2/M transition, is often accumulated in SMARCB1-deficient cells and disrupts APC/UBE2C to escape from mitotic stress [476]. These observations support the synthetic lethality between UBE2C and SMARCB1 [477]. Notably, SMARCA2 and SMARCA4 can co-occur in cancers [376], but loss of SMARCA4/BRG1 can also create a profound dependency on the ATPase activity of SMARCA2 [250]. Inhibiting miR-302a-3p is synthetically lethal with SMARCA2/BRM loss in PC cells because it increases SOCS5 levels and inhibits JAK2/STAT3 signaling [478]. On the other hand, SMARCA4-deficient cancers exhibit sensitivity to a broad range of inhibitors, including cell cycle-targeting agents (e.g., CDK4/6, AuroraA inhibitors), epigenetic modulators (BET and EZH2 inhibitors), metabolic disruptors (e.g., OXPHOS inhibitors), and DNA repair-targeting compounds (PARPi) [479].

Furthermore, ACTR5 has emerged as a synthetic lethal target in HCC [417]. Its loss activates CDKN2A expression and aborts CDK/E2F-driven cell cycle signaling that is essential for tumor growth. INO80C, a homolog of INO80, exhibits selective lethality in KRAS-mutant tumors (HCT116), presenting a synthetic lethal therapeutic opportunity for KRAS-driven solid tumors [480].

#### Direct targeting of remodeler proteins

A more direct approach involves inhibiting the ATPase activity of SWI/SNF complexes or selectively degrading specific subunits. As discussed in the previous sections, SWI/SNF inhibitors have demonstrated broad therapeutic potential across multiple tumor types. This is largely attributable to the dependence of core regulatory circuits on the open chromatin states established by SWI/SNF complexes. This mechanistic basis also explains why SWI/SNF inhibitors retain efficacy even in some rare tumors that lack intrinsic SWI/SNF subunit mutations [150]. Furthermore, cancers with loss-of-function mutations in SWI/SNF subunits such as SMARCA4 (BRG1) often exhibit increased dependency on the paralogous ATPase SMARCA2 (BRM) [375]. Even though SWI/SNF is usually inactivated in solid tumors, SMARCA2 is overexpressed in AML [405]. Consequently, SWI/SNF ATPase inhibitors represent a particularly attractive therapeutic strategy for these malignancies. BRM014, a fast-acting dual ATPase inhibitor targeting both SMARCA2 and SMARCA4, was initially characterized in BRG1-deficient lung cancer models and has now been used to probe the role of SWI/SNF functions across various cancer types [481]. FHD-286, a first-in-class BRG1/BRM ATPase inhibitor, is currently being evaluated in leukemia and melanoma [482]. Fiskus et al. demonstrated synergistic lethality with co-treatment of FHD-286 and Quizartinib or gilteritinib (FLT3 inhibitors) in AML cells harboring FLT3 mutations, regardless of TP53 status [405]. The group speculated that the FHD-286 sensitizes the FLT3 inhibitor-resistant cells through STAT5, pSTAT5, and AKT downregulation. Their work expands the potential clinical applications of SWI/SNF inhibitors across different combinations and AML subtypes [405].

A phase I clinical study of FHD-286 monotherapy in myeloid malignancies showed limited clinical activity and significant differentiation syndrome [483]. Nevertheless, significant reductions in peripheral and bone marrow leukemic burden were noted, especially in patients with enhancer-driven genotypes [483]. Further investigation of FHD-286 in combination with the existing regimen, Decitabine and Venetoclax in AML patients (NCT07283094) is currently underway. Additionally, EP400/TIP60 were identified to compensate for SWI/SNF inhibitor treatment through H2A-H2A.Z exchange and histone acetylation, which may contribute to therapeutic resistance or selectivity [484].

Due to the toxicity to normal tissues associated with the on-target effects of dual SMARCA2/4 inhibition, selective SMARCA2 inhibitors have emerged as an alternative strategy. Structure optimization from FHD-286 yielded FHD-909, which exhibits remarkable selectivity for SMARCA2 [485]. This compound is currently being evaluated in a Phase I trial (NCT06561685) for SMARCA4-mutant non-small cell lung cancer [485]. To further improve selectivity, various approaches have been employed, including computer-aided drug design (CADD) and Proteolysis-Targeting Chimera (PROTAC) technology [486]. Among these, PROTAC degraders have demonstrated particular advantages in achieving SMARCA2 selectivity. A947 is a potent and selective SMARCA2-targeting PROTAC degrader that demonstrates potent in vitro and in vivo efficacy in SMARCA4-mutant tumors with a favorable off-target profile [487].

PRT3789, a first-in-class clinical-stage SMARCA2-selective degrader (VHL-based PROTAC), forms a stable ternary complex with SMARCA2's unique lysine pocket to induce its ubiquitination and proteasomal degradation without affecting SMARCA4, and demonstrates preliminary clinical activity in SMARCA4-mutated NSCLC or esophageal cancer [488].

AU-15330, a PROTAC degrader of SMARCA2 and SMARCA4, inhibits tumor growth without an anti-proliferative effect on benign cells. As AU-15330 reduces chromatin accessibility at core enhancers, including AR, it synergizes with the AR antagonist enzalutamide in prostate cancer [295]. These data highlight the feasibility of targeting SWI/SNF complexes as a promising therapeutic strategy in cancer.

#### Modulation of other pathways

Finally, targeting pathways downstream of SWI/SNF dysfunction—such as angiogenesis, immune signaling, and epithelial-mesenchymal transition—offers additional therapeutic strategies. SWI/SNF alterations enhance ICI responses in pancreatic and renal cancers by increasing neoantigen burden or modulating interferon signaling [489, 490]. CHK1 inhibition enhances radiotherapy response via antitumor immunity in ARID1A-deficient CRC [261]. Anti-VEGF therapies target SWI/SNF-mutant cancers. For example, ARID1A-deficient HCC is sensitized to Ang2 blockade when combined with sorafenib [221]. ARID1A loss also suppresses PTGS1/PTGS2, sensitizing tumors to aspirin, an AA pathway inhibitor that enhances immunotherapy via CD8⁺ T-cell activation and inhibition of vasculogenic mimicry in colorectal cancer [491]. ROCK inhibitors show promise in SMARCA4-deficient breast cancer, and YAP inhibitors (e.g., VT3989) are in trials for ARID1A-mutant cervical cancer. SMARCA4 plays a critical antagonistic role in pancreatic cancer and promotes tumorigenesis by harboring a mesenchymal-like transcriptional landscape [233]. JQ1 inhibits HMGA2-mediated EMT and suppresses progenitor marker expression by inhibiting part of SMARCA4-mediated functions in pancreatic cancer [233]. Notably, agents such as eribulin can remodel chromatin to reverse EMT, underscoring the broad therapeutic potential of modulating chromatin accessibility and DNA damage responses in breast cancer treatment [492].

Targeting genetic vulnerabilities in remodeling-deficient tumors, such as DCAF5 in SMARCB1-mutant cancers or ROCK inhibitors in SMARCA4-deficient settings, has shown preclinical promise in breast cancer [143, 341]. In cervical cancers, amplifications of YAP and ARID1A are mutually exclusive [493]. Preclinically, YAP knockdown selectively inhibits the growth of ARID1A-mutant but not ARID1A-wildtype cells [493]. The ARID1A overexpression further augments resistance to the YAP inhibitor Verteporfin in ARID1A-mutant cells [318]. Several YAP inhibitors targeting the YAP/TAZ/TEAD complex, including VT3989, IK930, and IAG933, have advanced to clinical trials [494–496]. In synovial sarcoma, the targeted degradation of BRD9, a component of SS18-SSX fusion protein containing BAF complexes, selectively suppresses oncogenic transcriptional programs, highlighting BRD9 degradation as a promising therapeutic strategy for this fusion-driven malignancy [497].

### Therapeutic strategies targeting cohesin deficiency

Although numerous studies have demonstrated that cohesin mutations affect tumor progression and therapeutic response, no clinically approved drugs directly targeting cohesin are currently available, largely because cohesin is essential for normal chromatin organization and cell viability. However, it has been reported that cohesin mutations confer synthetic lethality to PARP inhibition [166]. Preclinical studies have delivered promising results in targeting leukemia with the PARP inhibitor Talazoparib [498]. A clinical trial evaluating Talazoparib in AML and MDS with cohesin complex mutations is currently underway (NCT03974217).

Taken together, these advances highlight alterations in chromatin remodeling factors, particularly in ARID1A and multiple SWI/SNF subunits, as central drivers of cancer initiation, progression, immune microenvironment, and treatment response, while also revealing a spectrum of targetable synthetic lethal vulnerabilities that may guide precision therapy in a molecularly defined patient population. Biomarker-driven patient selection remains critical for translating these synthetic lethal and epigenetic approaches into clinical practice.

### Outstanding questions: How can targeted degradation or synthetic lethality be applied without unacceptable toxicity in normal tissues? Which chromatin-remodeling defects generate actionable dependencies suitable for patient stratification?

Despite the promise of chromatin remodeler-targeted therapy, two major challenges remain. First, since the chromatin remodelers exist in both the tumor and the normal tissues, applying targeted degradation or synthetic lethality without unacceptable toxicity in normal tissues is a major concern. To reduce toxicity in normal tissues, multiple approaches are currently under investigation. For example, a GSH-inducible SMARCA2/4 PROTAC precursor is designed to release the active degrader only when it reaches the GSH-rich lung cancer cells, thereby improving tumor selectivity [499]. In synovial sarcoma, the compound ZZ7 covalently binds to DCAF16, an E3 ligase enriched in synovial sarcoma cells, enabling selective drug activity in tumor cells while sparing cardiomyocytes [500]. In addition, inspired by antibody–drug conjugates, the degrader-antibody conjugate for dual targeting represents another promising strategy [501]. Second, which specific alterations can reliably function as predictive biomarkers to guide targeted therapies needs further investigation. Multiple cancer types are frequently subject to inactivated mutations of SWI/SNF complexes. Under these conditions, tumor cells often become dependent on the paralogous SWI/SNF subunits for survival, so targeting these paralogs is an important therapeutic intervention. For instance, ARID1A-deficient tumors exhibit a synthetic lethal dependency on ARID1B or other epigenetic regulators such as HDAC and EZH2 [470, 502, 503]. In tumors that lack SWI/SNF mutations, the complexes may be recruited by other aberrant co-factors to sustain oncogenic transcription [147]. In such contexts, broad SWI/SNF inhibitors have demonstrated therapeutic potential. However, for tumors harboring cohesin complex mutations, there are not many options for now, except PARP inhibition [166]. Addressing these issues will require mechanistic insights into tissue-specific dependencies and biomarker-driven clinical trials to translate these approaches into clinical benefit.

## Challenges for developing therapeutic strategies targeting chromatin remodeling

Although several therapies targeting chromatin remodeling have already been approved for cancer patients (Table 1), clinical outcomes vary widely. Translating chromatin remodeler-based treatments still faces a few challenges.

**Table 1:** Approved therapies targeting chromatin remodeling for cancer treatment

Epigenetic drug	Target	First Date of approval (FDA/NMPA)	Approved cancer indication
Decitabine (5-aza-2′- deoxycytidine)	DNMT	2006	Acute myeloid leukemia (AML), myelodysplastic syndromes (MDS) and chronic myelomonocytic leukemia (CMML)
Azacitidine (CC-486)	DNMT	2004(i.v.); 2020(i.g.)	Acute myeloid leukemia (AML), juvenile myelomonocytic leukemia, myelodysplastic syndromes (MDS), chronic myelomonocytic leukemia (CMML), and juvenile myelomonocytic leukemia (JMML)
Vorinostat (SAHA)	HDAC	2006	Cutaneous T-cell lymphoma (CTCL)
Romidepsin (FK288)	HDAC	2009	Cutaneous T-cell lymphoma (CTCL), Peripheral T-cell lymphoma (PTCL)
Romidepsin	HDAC	2011	Durable clinical responses in patients with relapsed/refractory Peripheral T-cell lymphoma (PTCL)
Belinostat (PXD101)	HDAC	2014	Peripheral T-cell lymphoma (PTCL)
Panobinostat (LBH589)	HDAC	2015	Multiple myeloma and Cutaneous T-cell lymphoma (CTCL)
Chidamide	HDAC	2014	Peripheral T-cell lymphoma (PTCL)
Tazemetostat	HMT/EZH2	2020/2025	Epithelioid sarcoma and follicular lymphoma
SHR2554	HMT/EZH2	2025	Peripheral T-cell lymphoma (PTCL)

*Abbreviations: DNMT* DNA methyltransferase, *HDAC* Histone deacetylase, *HMT* Histone methyltransferase, *FDA* U.S. Food and Drug Administration, *NMPA* National Medical Products Administration

### Functions of chromatin remodelers are highly context-dependent in cancer progression

The role of chromatin remodeling in carcinogenesis is profoundly context-related. The same remodeler, such as SMARCA4/ARID1A, can function as either an oncoprotein or a tumor suppressor [192, 504, 505]. This dual role of chromatin remodelers in cancer promotion makes it difficult to develop a therapeutic agent, as it could cause a ‘same target, complete opposite outcome’ scenario in treating cancer patients.

This functional versatility arises from several interconnected mechanisms. First, the compositional and conformational flexibility of remodeler complexes generates extensive heterogeneity [506]. The combinatory assembly of mutually exclusive paralogues at multiple positions can theoretically yield thousands of distinct complex variants, each with unique genomic targets and activities [507]. Second, the remodeler function is shaped by cell-type-specific gene expression and interactions with TFs. Many paralogues and isoforms are expressed in a tissue-specific manner, enabling context-specific activity [508]. Their genomic localization is further influenced by interactions with TFs, histone modifications, histone variants, extranucleosomal DNA, and higher-order chromatin remodelers [508]. Therefore, the specific promoters, enhancers, and super enhancers they regulate vary across cellular contexts.

### The majority of chromatin remodeling mutations are loss-of-function, making it difficult to target

Therapeutically targeting chromatin remodelers is fundamentally constrained by their mutational landscape. Unlike oncogenes that acquire gain-of-function alterations, many chromatin remodeler-related mutations are loss-of-function, such as those affecting ARID1A, STAG2, and PDS5B [509]. The loss of these tumor suppressors makes it difficult to develop a direct targeted therapy since the major druggable logic is still inhibiting/blocking or degrading hyperactive proteins. Hence, therapeutic intervention must pivot towards exploiting vulnerabilities that arise as indirect consequences of remodeler loss, such as DNA damage response and repair, DNA replication stress, senescence bypass, metastasis, angiogenesis, and tumor immunity [506].

### Therapies aiming at chromatin irregularities usually develop resistance in a short period of time

Despite the initial efficacy of epigenetic modulators, including DNA methyltransferase inhibitors (DNMTis), histone deacetylase inhibitors (HDACis), and chromatin remodeler inhibitors, in reversing aberrant gene silencing and overcoming acquired chemoresistance, these responses are typically transient [510–513]. Unlike other small-molecule inhibitors targeting hyperactive proteins, in which resistance arises through new genetic mutations, these epigenetic modulators rapidly develop resistance due to epigenetic plasticity [514]. The inherent ability of cancer cells to reversibly modify their chromatin landscape and activate redundant survival pathways to bypass pharmacologic blockade. This adaptive capacity is compounded by the pharmacological limitations of small-molecule inhibitors themselves, including short half-lives, off-target effects, and efflux transporter dysregulation [515, 516].

### Current chromatin remodeler degraders present non-specific side effects

Unlike inhibitors that temporarily block protein activity, chromatin remodeler degraders induce permanent protein elimination. This complete LOF phenotype raises significant safety concerns given the dual functional roles of remodelers and the compositional diversity of these complexes, as discussed. While targeting a single subunit may inadvertently disrupt multiple complexes, systemic degradation carries the risk of oncogenic effects in tissues where the target normally functions as a tumor suppressor. These on-target, off-tumor toxicities create a narrow therapeutic window and require careful dose optimization. Consequently, addressing these limitations demands delivery strategies that enable tumor-selective targeting, such as nanoparticle encapsulation, antibody-degrader conjugates, or tumor-specific promoters, to confine degradation to malignant cells while sparing healthy tissues [517–519].

### The mechanism of cohesin complex regulation remains largely unknown

Despite cohesin's essential roles in loop extrusion, TAD formation, and DNA damage response, the molecular logic governing its recruitment, loading, movement, and release remains undefined. Thus, it hinders the treatment of cohesin-mutant malignancies. This regulation is inherently difficult to decipher for several reasons. First, cohesin often engages chromatin remodelers (e.g., SMARCA4, SNF2h), mediator complexes, and TFs for locus-specific recruitment, while emerging evidence implicates eRNAs also interacting with cohesin as direct regulators of loop extrusion [520]. Second, context-dependence and functional diversity between paralogue subunits obscure the mechanism (i.e., STAG1 disrupts Polycomb interactions, whereas STAG2 recruits PRC1 to promote local compaction) [521]. Consequently, despite the prevalence of cohesin mutations in cancer, we cannot predict how specific alterations disrupt loop extrusion or enhancer–promoter communication.

### Direct targeting of the cohesin complex is mostly lethal to normal cells

The therapeutic challenge is partly contributed to by cohesin’s essential role in cellular homeostasis. Complete loss of cohesin disrupts sister chromatin cohesion and DNA loop domains and largely dysregulates programmed transcriptomes [522]. However, cancer-associated cohesin mutations are rarely complete LOF; the majority are missense mutations that subtly rewire cohesin function while preserving sufficient core cohesion for cell survival [523]. Therefore, a future direction focused on identifying context-specific vulnerabilities in cohesin-mutant cells and developing strategies to rewire cohesin-mediated loops, rather than inhibit cohesin itself, would be promising.

## Future directions

### Targeting chromatin remodelers by a specific degrading strategy

The limitations of conventional inhibition are the lack of specificity and the generation of off-target effects. As crucial modulators of genome stability and transcription, chromatin remodelers that are targeted regardless of context could be toxic to normal tissues. Therefore, a more specific blocking strategy for future inhibitors is necessary.

PROTACs enable simultaneous binding to form a ternary complex, leading to target ubiquitination and proteasomal degradation. This catalytic mechanism confers distinct advantages: 1) a single PROTAC can eliminate multiple target copies, overcoming resistance caused by protein overexpression; 2) PROTACs require only target binding rather than functional inhibition, increasing proteome coverage and preventing off-target effects on proteins with binding site mutations [524]. Specifically, PROTACs like ACBI1 and VZ185 have shown powerful degradative capabilities towards SMARCA2/4 and ncBAF subunits, respectively [525, 526]. Meanwhile, molecular glues represent an orthogonal strategy. They induce or stabilize neo-protein–protein interactions between an E3 ligase and a target protein, leading to degradation [527, 528]. Their lower molecular weight confers enhanced membrane permeability and oral bioavailability, aligning more closely with conventional drug-likeness [529]. Ongoing efforts focus on resolving challenges, such as long-term safety and poor solubility, to facilitate translational use.

Certainly, how we really address their off-tumor effects remains a continual subject for discussion. One promising strategy will be engineering the protein degraders such that it is only delivered or activated in the tumor microenvironment [530]. A dual-targeted approach that requires disease-specific antigen engagement and disease-activatable pro-PROTAC has shown a promising pre-clinical safety profile and synergism with radiotherapy [531].

### Rewire abnormal topologies by Transcriptional chemical inducers of proximity (TCIP)

While PROTACs and molecular glues hold promise for gain-of-function alterations, they are rendered useless in chromatin remodeling loss-of-function mutation events. This therefore highlights the need to identify mutation-specific vulnerabilities and synthetic-lethal interactions. In these cases, it seems reasonable to employ a gain-of-function strategy.

One example is transcriptional chemical inducers of proximity (TCIPs). These offer an emerging alternative to protein degradation. These bifunctional small molecules forcibly recruit TFs to target genes and activate their expression, including apoptotic genes and tumor suppressors silenced by remodeller LOF mutations [532, 533]. Unlike degraders, TCIPs preserve normal protein functions in healthy tissues, avoiding on-target, off-tumor toxicity [534].

### To create synthetic lethality in cancer cells with chromatin remodeler mutations

Other than the gain-of-function approach, synthetic-lethality interactions can be exploited. In the sections above, we have introduced quite a few of the synthetic lethality therapeutic strategies for treating chromatin remodeler LOFs. Another direction could be targeting the HR mechanism. This is because among the chromatin remodeler LOF mutants, HR repair is frequently impaired [535]. This HR deficiency (HRD) creates a synthetic lethal vulnerability, where inhibition of the complementary DDR pathway, e.g., with PARP inhibitors, can selectively kill tumor cells while sparing normal tissues. PARP inhibitors trap PARP1 at single-strand breaks, leading to replication fork collapse and accumulation of double-strand breaks that HRD cancer cells cannot repair [536]. With intact HR, normal cells can tolerate this insult, thus creating a therapeutic window. However, this therapeutic strategy is not foolproof either. Not only does PARPi cause toxicities, but chromatin remodeler mutations are also often accompanied by upregulated compensatory pathways such as PI3K/Akt that confer PARPi resistance [537]. To overcome and delay PARPi resistance, exploring combinatorial treatment regimens and developing novel DDR inhibitors for specific remodeller-mutant contexts appear to be a continual effort [538, 539].

### Employing novel technologies and platforms for future biomarker or therapeutic target identification

Since the function of chromatin remodelers is highly context-dependent, they could function as either tumor suppressors or oncogenic factors, as mentioned above. In this regard, future studies should employ lineage-specific screens, single-cell multi-omics, and disease-relevant models. For investigating cohesin mutation-caused genome topology changes, technologies like Micro-C [19], HiChIP [540], and live-cell imaging are necessary. Furthermore, since many cohesin factors are responsible for the cell cycle, studying their functions needs acute degron systems, such as the AID-Auxin-induced degron system for RAD21, SMC3, or CTCF [541, 542]. These technologies would also facilitate the evaluation of the patient’s chromatin state and architecture, thereby further supporting patient stratification.

### Restoring Antitumor Immunity Suppressed by Chromatin Remodeling Defects through Epigenetic Targeting and Immunotherapy

Tumors employ multiple immune evasion strategies, including secreting immunosuppressive cytokines, overexpressing PD-L1 to inhibit T cells, recruiting immunosuppressive cells, and metabolic reprogramming that impairs immune function [513, 543]. Chromatin remodeling mutations can exacerbate this suppression. For instance, SMARCA4-mutant lung cancers lose chromatin accessibility at immune regulatory genes, disrupting immune cell infiltration, interferon signaling, and antigen presentation, leading to poor anti-PD-1 therapy response [513, 544]. However, these epigenetic defects create targetable vulnerabilities. Epigenetic drugs such as DNMT and HDAC inhibitors can re-express MHC-I and tumor-associated antigen [545]. EZH2 inhibitors reprogram the tumor microenvironment, and combining these agents with immune checkpoint inhibitors has shown efficacy across several cancers [543]. Thus, understanding how specific remodeling mutations suppress immunity enables the rational combination of therapies that counteract anti-tumor immune suppression and enhance immunotherapy efficacy.

### Personalized therapeutic strategies

The context-dependent functions of chromatin remodelers dictate a personalized approach to therapeutic selection. Treatment decisions must be guided by specific mutation context, tissue of origin, and co-occurring alterations. Single-cell sequencing technologies now offer the resolution to map remodeler complex heterogeneity and identify predictive biomarkers for patient stratification [546, 547]. Furthermore, apart from single-gene mutations, integrating the patient’s chromatin state, 3D genome architecture alterations, transcription dependency, and immune contexture via all sorts of novel sequencing/imaging technologies would contribute to multimodal biomarker selection.

Additionally, expanding screening beyond common driver genes to include systematic drug sensitivity profiling will accelerate the discovery of clinically actionable synthetic lethal interactions. Combination therapies offer enhanced efficacy but carry risks of drug interactions and adverse events, demanding strict patient selection [516]. Ultimately, moving from empirical combinations to rationally designed, personalized plans requires a mechanistic understanding sufficient to match the right drug to the right patient.

### To further investigate the linkage between 2D chromatin remodelers and the cohesin complex

To date, the relationship between the 2D chromatin remodelers and the cohesin complex remains largely unknown. Past studies have linked several classic remodelers to the cohesin complex subunits, such as the linkage between ARID1A and STAG1 [548]. Interestingly, SMARCA2/4 degrader-treated cells exhibited open chromatin accessibility for CTCF binding sites [295], indicating a potential correlation between the SWI/SNF complex and the cohesin complex. Perhaps, they have a competitive relationship regarding chromatin binding. Future studies could focus on relationships like this by employing ChIPseq, ATACseq, and HiC analysis on chromatin remodelers and cohesin complex subunits. Eventually, this could also lead to repurposing therapies targeting chromatin remodelers for patients carrying cohesin-related mutations.

## Conclusion

Advances in Hi-C technologies and the discovery of novel chromatin remodeling complexes have expanded our view of chromatin remodeling from local, ATP-dependent nucleosome sliding to a higher-order, multiscale process that shapes the entire nuclear landscape. As reviewed here, first-level chromatin remodelers (SWI/SNF, ISWI, CHD, IN80) and higher-order complexes (cohesin, CTCF) cooperate to shape the 3D chromatin architecture. Beyond controlling chromatin accessibility for TF and RNA polymerase binding, first-level remodelers facilitate TAD formation by regulating nucleosome occupancy at CTCF and cohesion binding sites. Conversely, the 3D genome structure established by cohesion creates microenvironments that facilitate or restrict first-level remodeler activity. Linear genetic accessibility and spatial proximity are thus functionally inseparable.

Alterations in chromatin remodelers are prevalent across diverse cancers. However, therapeutic development is hindered by the profound context-dependency of these complexes. Their opposing roles, influenced by cell of origin, signaling environment, and specific mutational landscape, complicate single-agent strategies and increase the risk of systemic toxicity. However, prioritizing the identification of lineage-specific vulnerabilities and paralog dependencies to create an unnatural, targetable dependency that is absent in normal tissues would be a robust next step.

The prevalence of LOF mutations in tumor suppressors presents a classic undruggable challenge. The field is therefore pivoting towards exploiting the resulting cellular state through mechanisms such as synthetic lethality, epigenetic modification, and transcriptional reprogramming. Despite significant progress, critical gaps remain. The mechanisms of cohesin regulation and the cooperation between diverse remodelers and other regulatory elements remain incompletely defined. Hence, novel high-resolution imaging platforms and in vivo reconstitution models are urgently needed. Molecular profiling of individual patients will further advance the development of personalized treatment by optimizing combination therapies, discovering novel synthetic lethality-based treatment plans, and ultimately maximizing therapeutic response.

## Data Availability

Data sharing is not applicable to this article as no datasets were generated or analysed during the current study.

## Abbreviations

BRM: Drosophila Brahma
SWI/SNF: Switch/sucrose nonfermentable
ISWI: Imitation Switch
INO80: Inositol-requiring mutant 80
CHD: Chromodomain-helicase DNA-binding protein
3D: Three-dimensional
3C: Capturing chromosome conformation
4C: Circular capturing chromosome conformation
5C: Chromosome conformation capture carbon copy
CTCF: CCCTC-binding factor
TAD: Topologically associating domain
BAF: BRG1/BRM-associated factor
cBAF: Canonical BAF
pBAF: Polybromo-associated BAF
ncBAF: Non-canonical BAF
HAS: Helicase-SANT-associated
CHROMO: Chromatin organizing domain
PHD: Plant homeodomain
BRK: Brahma and Kismet domain
NuRD: Nucleosome remodeling and deacetylase
Arp: Actin-related proteins
NIPBL: Nipped-B-like protein
MAU2: MAU2 chromatid cohesion factor homolog
WAPL: Wings apart-like protein homolog
PDS5A/B: Sister chromatid cohesion protein PDS5 homolog A/B
LOF: Loss-of-function
PTM: Post-translational modification
LLPS: Liquid-liquid phase segregation
RSC: Remodeling structure of chromatin
RNAPII: RNA polymerase II
PTF: Pioneer transcription factor
AR: Androgen receptor
eRNA: Enhancer RNA
TERRA: Telomeric repeat-encoding RNA
PLD: Prion-like domain
DSB: Double-strand break
HR: Homologous recombination
NHEJ: Non-homologous end joining
DDR: DNA damage repair
PARPi: Poly(ADP-ribose) polymerase inhibitor
PcG: Polycomb group
TrxG: Trithorax group
PRC2: Polycomb repressive complex 2
OXPHOS: Oxidative phosphorylation
GSH: Glutathione
ROS: Reactive oxygen species
TME: Tumor microenvironment
ICI: Immune-checkpoint inhibition
CRC: Colorectal cancer
MeC: Colorectal medullary carcinoma
TCGA: The Cancer Genome Atlas
MSS: Microsatellite-stable
EMT: Epithelial-mesenchymal transition
TAF: Tumor-associated fibroblast
GC: Gastric cancer
MSI: Microsatellite instability
EBV: Epstein-Barr virus
EBVaGC: Epstein-Barr virus-associated gastric cancer
EBVnGC: EBV-negative gastric cancer
HCC: Hepatocellular carcinoma
WES: Whole-exome sequencing
WGS: Whole-genome sequencing
HBV: Hepatitis B virus
Ang2: Angiopoietin-2
NER: Nucleotide excision repair
PDAC: Pancreatic ductal adenocarcinoma
PanIN: Pancreatic intraepithelial neoplastic lesion
IPMN: Intraductal papillary mucinous neoplasm
TSS: Transcription start site
ADM: Acinar-to-ductal metaplasia
PDC: Pancreatic ductal cell
EAC: Esophageal adenocarcinoma
ESCC: Esophageal squamous cell carcinoma
TMB-H: High tumor mutational burden
TNBC: Triple-negative breast cancer
PFS: Progression-free survival
SCCOHT: Small cell carcinoma of the ovary, hypercalcemic type
WHO: World Health Organization
PCa: Prostate cancer
EBRT: External-beam radiotherapy
ADT: Androgen deprivation therapy
CRPC: Castration-resistant prostate cancer
CRPC-NE: Neuroendocrine Pca
dCRT: Definitive chemoradiotherapy
CIN: Cervical intraepithelial neoplasia
EEC: Endometrioid endometrial carcinoma
UEC: Undifferentiated endometrial carcinoma
ARNTL: Aryl hydrocarbon receptor nuclear translocator-like
RC: Renal cancer
RCC: Renal cell carcinoma
PRCC: Papillary renal cell carcinoma
ccRCC: Clear cell renal carcinoma
RMC: Renal medullary carcinoma
NSCLC: Non-small cell lung cancer
SCLC: Small cell lung cancer
CCLE: Cancer Cell Line Encyclopedia
CBX2: Chromobox Homolog 2
LT-HSC: Long-term repopulating hematopoietic stem cell
B-ALL: B-cell acute lymphoblastic leukemia
AML: Acute myeloid leukemia
FLT3-ITD: FLT3-gain-of-function internal tandem duplication
HL: Hodgkin lymphoma
NHL: Non-Hodgkin lymphoma
FL: Follicular lymphoma
DLBCL: Diffuse large B-cell lymphoma
ATRT: Atypical teratoid rhabdoid tumor
PDTC: Poorly differentiated thyroid cancer
ATC: Anaplastic thyroid cancer
RMS: Rhabdomyo sarcoma
AURKA: Aurora kinase A
HDAC: Histone deacetylase
CADD: Computer-aided drug design
PROTAC: Proteolysis-Targeting Chimera
DNMTi: DNA methyltransferase inhibitor
HDACi: Histone deacetylase inhibitor
TCIP: Transcriptional chemical inducers of proximity
HRD: HR deficiency

## References

[1] Paul J. General theory of chromosome structure and gene activation in eukaryotes. Nature. 1972;238(5365):444–6. 4561852 10.1038/238444a0

[2] Paranjape SM, Kamakaka RT, Kadonaga JT. Role of chromatin structure in the regulation of transcription by RNA polymerase II. Annu Rev Biochem. 1994;63:265–97. 7979240 10.1146/annurev.bi.63.070194.001405

[3] Stern M, Jensen R, Herskowitz I. Five SWI genes are required for expression of the HO gene in yeast. J Mol Biol. 1984;178(4):853–68. 6436497 10.1016/0022-2836(84)90315-2

[4] Mashtalir N, D’Avino AR, Michel BC, Luo J, Pan J, Otto JE, et al. Modular Organization and Assembly of SWI/SNF Family Chromatin Remodeling Complexes. Cell. 2018;175(5):1272-88.e20. 30343899 10.1016/j.cell.2018.09.032 PMC6791824

[5] Dingwall AK, Beek SJ, McCallum CM, Tamkun JW, Kalpana GV, Goff SP, et al. The Drosophila snr1 and brm proteins are related to yeast SWI/SNF proteins and are components of a large protein complex. Mol Biol Cell. 1995;6(7):777–91. 7579694 10.1091/mbc.6.7.777 PMC301240

[6] Tamkun JW, Deuring R, Scott MP, Kissinger M, Pattatucci AM, Kaufman TC, et al. brahma: a regulator of Drosophila homeotic genes structurally related to the yeast transcriptional activator SNF2/SWI2. Cell. 1992;68(3):561–72. 1346755 10.1016/0092-8674(92)90191-e

[7] Okabe I, Bailey LC, Attree O, Srinivasan S, Perkel JM, Laurent BC, et al. Cloning of human and bovine homologs of SNF2/SWI2: a global activator of transcription in yeast *S. cerevisiae*. Nucleic Acids Res. 1992;20(17):4649–55. 1408766 10.1093/nar/20.17.4649 PMC334196

[8] Elfring LK, Deuring R, McCallum CM, Peterson CL, Tamkun JW. Identification and characterization of Drosophila relatives of the yeast transcriptional activator SNF2/SWI2. Mol Cell Biol. 1994;14(4):2225–34. 7908117 10.1128/mcb.14.4.2225-2234.1994 PMC358589

[9] Aihara T, Miyoshi Y, Koyama K, Suzuki M, Takahashi E, Monden M, et al. Cloning and mapping of SMARCA5 encoding hSNF2H, a novel human homologue of Drosophila ISWI. Cytogenet Cell Genet. 1998;81(3–4):191–3. 9730600 10.1159/000015027

[10] Peterson CL, Tamkun JW. The SWI-SNF complex: a chromatin remodeling machine? Trends Biochem Sci. 1995;20(4):143–6. 7770913 10.1016/s0968-0004(00)88990-2

[11] DiLauro R, Taniguchi T, Musso R, de Crombrugghe B. Unusual location and function of the operator in the Escherichia coli galactose operon. Nature. 1979;279(5713):494–500. 221831 10.1038/279494a0

[12] Grosschedl R, Birnstiel ML. Identification of regulatory sequences in the prelude sequences of an H2A histone gene by the study of specific deletion mutants in vivo. Proc Natl Acad Sci U S A. 1980;77(3):1432–6. 6929494 10.1073/pnas.77.3.1432 PMC348509

[13] Dunn TM, Hahn S, Ogden S, Schleif RF. An operator at -280 base pairs that is required for repression of araBAD operon promoter: addition of DNA helical turns between the operator and promoter cyclically hinders repression. Proc Natl Acad Sci U S A. 1984;81(16):5017–20. 6089170 10.1073/pnas.81.16.5017 PMC391628

[14] Wasylyk B, Wasylyk C, Chambon P. Short and long range activation by the SV40 enhancer. Nucleic Acids Res. 1984;12(14):5589–608. 6087293 10.1093/nar/12.14.5589 PMC320017

[15] Dekker J, Rippe K, Dekker M, Kleckner N. Capturing chromosome conformation. Science. 2002;295(5558):1306–11. 11847345 10.1126/science.1067799

[16] Zhao Z, Tavoosidana G, Sjölinder M, Göndör A, Mariano P, Wang S, et al. Circular chromosome conformation capture (4C) uncovers extensive networks of epigenetically regulated intra- and interchromosomal interactions. Nat Genet. 2006;38(11):1341–7. 17033624 10.1038/ng1891

[17] Dostie J, Richmond TA, Arnaout RA, Selzer RR, Lee WL, Honan TA, et al. Chromosome Conformation Capture Carbon Copy (5C): a massively parallel solution for mapping interactions between genomic elements. Genome Res. 2006;16(10):1299–309. 16954542 10.1101/gr.5571506 PMC1581439

[18] Lieberman-Aiden E, van Berkum NL, Williams L, Imakaev M, Ragoczy T, Telling A, et al. Comprehensive mapping of long-range interactions reveals folding principles of the human genome. Science. 2009;326(5950):289–93. 19815776 10.1126/science.1181369 PMC2858594

[19] Hsieh TH, Weiner A, Lajoie B, Dekker J, Friedman N, Rando OJ. Mapping Nucleosome Resolution Chromosome Folding in Yeast by Micro-C. Cell. 2015;162(1):108–19. 26119342 10.1016/j.cell.2015.05.048 PMC4509605

[20] Hughes JR, Roberts N, McGowan S, Hay D, Giannoulatou E, Lynch M, et al. Analysis of hundreds of cis-regulatory landscapes at high resolution in a single, high-throughput experiment. Nat Genet. 2014;46(2):205–12. 24413732 10.1038/ng.2871

[21] Dixon JR, Selvaraj S, Yue F, Kim A, Li Y, Shen Y, et al. Topological domains in mammalian genomes identified by analysis of chromatin interactions. Nature. 2012;485(7398):376–80. 22495300 10.1038/nature11082 PMC3356448

[22] Nora EP, Lajoie BR, Schulz EG, Giorgetti L, Okamoto I, Servant N, et al. Spatial partitioning of the regulatory landscape of the X-inactivation centre. Nature. 2012;485(7398):381–5. 22495304 10.1038/nature11049 PMC3555144

[23] Alipour E, Marko JF. Self-organization of domain structures by DNA-loop-extruding enzymes. Nucleic Acids Res. 2012;40(22):11202–12. 23074191 10.1093/nar/gks925 PMC3526278

[24] Sexton T, Yaffe E, Kenigsberg E, Bantignies F, Leblanc B, Hoichman M, et al. Three-dimensional folding and functional organization principles of the Drosophila genome. Cell. 2012;148(3):458–72. 22265598 10.1016/j.cell.2012.01.010

[25] Davidson IF, Bauer B, Goetz D, Tang W, Wutz G, Peters JM. DNA loop extrusion by human cohesin. Science. 2019;366(6471):1338–45. 31753851 10.1126/science.aaz3418

[26] Clapier CR, Cairns BR. The biology of chromatin remodeling complexes. Annu Rev Biochem. 2009;78:273–304. 19355820 10.1146/annurev.biochem.77.062706.153223

[27] Mittal P, Roberts CWM. The SWI/SNF complex in cancer - biology, biomarkers and therapy. Nat Rev Clin Oncol. 2020;17(7):435–48. 32303701 10.1038/s41571-020-0357-3 PMC8723792

[28] Wilson BG, Roberts CW. SWI/SNF nucleosome remodellers and cancer. Nat Rev Cancer. 2011;11(7):481–92. 21654818 10.1038/nrc3068

[29] Wang W, Côté J, Xue Y, Zhou S, Khavari PA, Biggar SR, et al. Purification and biochemical heterogeneity of the mammalian SWI-SNF complex. Embo j. 1996;15(19):5370–82. 8895581 PMC452280

[30] Lemon B, Inouye C, King DS, Tjian R. Selectivity of chromatin-remodelling cofactors for ligand-activated transcription. Nature. 2001;414(6866):924–8. 11780067 10.1038/414924a

[31] Alpsoy A, Dykhuizen EC. Glioma tumor suppressor candidate region gene 1 (GLTSCR1) and its paralog GLTSCR1-like form SWI/SNF chromatin remodeling subcomplexes. J Biol Chem. 2018;293(11):3892–903. 29374058 10.1074/jbc.RA117.001065 PMC5858003

[32] Kaeser MD, Aslanian A, Dong MQ, Yates JR 3rd, Emerson BM. BRD7, a novel PBAF-specific SWI/SNF subunit, is required for target gene activation and repression in embryonic stem cells. J Biol Chem. 2008;283(47):32254–63. 18809673 10.1074/jbc.M806061200 PMC2583284

[33] Yuan J, Chen K, Zhang W, Chen Z. Structure of human chromatin-remodelling PBAF complex bound to a nucleosome. Nature. 2022;605(7908):166–71. 35477757 10.1038/s41586-022-04658-5

[34] Moro N, Fujisawa-Tanaka Y, Watanabe S. ARID1A regulates histone octamer transfer activity of human canonical BAF complex. Nucleic Acids Res. 2025;53(18). 41017120 10.1093/nar/gkaf958 PMC12476982

[35] Wang X, Nagl NG, Wilsker D, Van Scoy M, Pacchione S, Yaciuk P, et al. Two related ARID family proteins are alternative subunits of human SWI/SNF complexes. Biochem J. 2004;383(Pt 2):319–25. 15170388 10.1042/BJ20040524 PMC1134073

[36] Michel BC, D’Avino AR, Cassel SH, Mashtalir N, McKenzie ZM, McBride MJ, et al. A non-canonical SWI/SNF complex is a synthetic lethal target in cancers driven by BAF complex perturbation. Nat Cell Biol. 2018;20(12):1410–20. 30397315 10.1038/s41556-018-0221-1 PMC6698386

[37] Wang X, Wang S, Troisi EC, Howard TP, Haswell JR, Wolf BK, et al. BRD9 defines a SWI/SNF sub-complex and constitutes a specific vulnerability in malignant rhabdoid tumors. Nat Commun. 2019;10(1):1881. 31015438 10.1038/s41467-019-09891-7 PMC6479050

[38] Saha A, Wittmeyer J, Cairns BR. Chromatin remodelling: the industrial revolution of DNA around histones. Nat Rev Mol Cell Biol. 2006;7(6):437–47. 16723979 10.1038/nrm1945

[39] Nakayama RT, Pulice JL, Valencia AM, McBride MJ, McKenzie ZM, Gillespie MA, et al. SMARCB1 is required for widespread BAF complex-mediated activation of enhancers and bivalent promoters. Nat Genet. 2017;49(11):1613–23. 28945250 10.1038/ng.3958 PMC5803080

[40] Mathur R, Alver BH, San Roman AK, Wilson BG, Wang X, Agoston AT, et al. ARID1A loss impairs enhancer-mediated gene regulation and drives colon cancer in mice. Nat Genet. 2017;49(2):296–302. 27941798 10.1038/ng.3744 PMC5285448

[41] Wang X, Lee RS, Alver BH, Haswell JR, Wang S, Mieczkowski J, et al. SMARCB1-mediated SWI/SNF complex function is essential for enhancer regulation. Nat Genet. 2017;49(2):289–95. 27941797 10.1038/ng.3746 PMC5285474

[42] Gatchalian J, Malik S, Ho J, Lee DS, Kelso TWR, Shokhirev MN, et al. A non-canonical BRD9-containing BAF chromatin remodeling complex regulates naive pluripotency in mouse embryonic stem cells. Nat Commun. 2018;9(1):5139. 30510198 10.1038/s41467-018-07528-9 PMC6277444

[43] Wiest NE, Houghtaling S, Sanchez JC, Tomkinson AE, Osley MA. The SWI/SNF ATP-dependent nucleosome remodeler promotes resection initiation at a DNA double-strand break in yeast. Nucleic Acids Res. 2017;45(10):5887–900. 28398510 10.1093/nar/gkx221 PMC5449591

[44] Kadoch C, Crabtree GR. Mammalian SWI/SNF chromatin remodeling complexes and cancer: Mechanistic insights gained from human genomics. Sci Adv. 2015;1(5):e1500447. 26601204 10.1126/sciadv.1500447 PMC4640607

[45] Kadoch C, Hargreaves DC, Hodges C, Elias L, Ho L, Ranish J, et al. Proteomic and bioinformatic analysis of mammalian SWI/SNF complexes identifies extensive roles in human malignancy. Nat Genet. 2013;45(6):592–601. 23644491 10.1038/ng.2628 PMC3667980

[46] Li Y, Gong H, Wang P, Zhu Y, Peng H, Cui Y, et al. The emerging role of ISWI chromatin remodeling complexes in cancer. J Exp Clin Cancer Res. 2021;40(1):346. 34736517 10.1186/s13046-021-02151-x PMC8567610

[47] Grüne T, Brzeski J, Eberharter A, Clapier CR, Corona DF, Becker PB, et al. Crystal structure and functional analysis of a nucleosome recognition module of the remodeling factor ISWI. Mol Cell. 2003;12(2):449–60. 14536084 10.1016/s1097-2765(03)00273-9

[48] Clapier CR, Cairns BR. Regulation of ISWI involves inhibitory modules antagonized by nucleosomal epitopes. Nature. 2012;492(7428):280–4. 23143334 10.1038/nature11625 PMC3631562

[49] Tsukiyama T, Daniel C, Tamkun J, Wu C. ISWI, a member of the SWI2/SNF2 ATPase family, encodes the 140 kDa subunit of the nucleosome remodeling factor. Cell. 1995;83(6):1021–6. 8521502 10.1016/0092-8674(95)90217-1

[50] Tsukiyama T, Wu C. Purification and properties of an ATP-dependent nucleosome remodeling factor. Cell. 1995;83(6):1011–20. 8521501 10.1016/0092-8674(95)90216-3

[51] Varga-Weisz PD, Wilm M, Bonte E, Dumas K, Mann M, Becker PB. Chromatin-remodelling factor CHRAC contains the ATPases ISWI and topoisomerase II. Nature. 1997;388(6642):598–602. 9252192 10.1038/41587

[52] Ito T, Bulger M, Pazin MJ, Kobayashi R, Kadonaga JT. ACF, an ISWI-containing and ATP-utilizing chromatin assembly and remodeling factor. Cell. 1997;90(1):145–55. 9230310 10.1016/s0092-8674(00)80321-9

[53] Bozhenok L, Wade PA, Varga-Weisz P. WSTF-ISWI chromatin remodeling complex targets heterochromatic replication foci. Embo j. 2002;21(9):2231–41. 11980720 10.1093/emboj/21.9.2231 PMC125993

[54] Strohner R, Nemeth A, Jansa P, Hofmann-Rohrer U, Santoro R, Längst G, et al. NoRC–a novel member of mammalian ISWI-containing chromatin remodeling machines. Embo j. 2001;20(17):4892–900. 11532953 10.1093/emboj/20.17.4892 PMC125270

[55] Badenhorst P, Voas M, Rebay I, Wu C. Biological functions of the ISWI chromatin remodeling complex NURF. Genes Dev. 2002;16(24):3186–98. 12502740 10.1101/gad.1032202 PMC187504

[56] Radzisheuskaya A, Peña-Rømer I, Lorenzini E, Koche R, Zhan Y, Shliaha PV, et al. An alternative NURF complex sustains acute myeloid leukemia by regulating the accessibility of insulator regions. Embo j. 2023;42(24):e114221. 37987160 10.15252/embj.2023114221 PMC10711654

[57] Delmas V, Stokes DG, Perry RP. A mammalian DNA-binding protein that contains a chromodomain and an SNF2/SWI2-like helicase domain. Proc Natl Acad Sci U S A. 1993;90(6):2414–8. 8460153 10.1073/pnas.90.6.2414 PMC46097

[58] Woodage T, Basrai MA, Baxevanis AD, Hieter P, Collins FS. Characterization of the CHD family of proteins. Proc Natl Acad Sci U S A. 1997;94(21):11472–7. 9326634 10.1073/pnas.94.21.11472 PMC23509

[59] Mansfield RE, Musselman CA, Kwan AH, Oliver SS, Garske AL, Davrazou F, et al. Plant homeodomain (PHD) fingers of CHD4 are histone H3-binding modules with preference for unmodified H3K4 and methylated H3K9. J Biol Chem. 2011;286(13):11779–91. 21278251 10.1074/jbc.M110.208207 PMC3064229

[60] Mills AA. The Chromodomain Helicase DNA-Binding Chromatin Remodelers: Family Traits that Protect from and Promote Cancer. Cold Spring Harb Perspect Med. 2017;7(4):a026450. 28096241 10.1101/cshperspect.a026450 PMC5378010

[61] Alendar A, Berns A. Sentinels of chromatin: chromodomain helicase DNA-binding proteins in development and disease. Genes Dev. 2021;35(21–22):1403–30. 34725129 10.1101/gad.348897.121 PMC8559672

[62] Thompson PM, Gotoh T, Kok M, White PS, Brodeur GM. CHD5, a new member of the chromodomain gene family, is preferentially expressed in the nervous system. Oncogene. 2003;22(7):1002–11. 12592387 10.1038/sj.onc.1206211

[63] Bagchi A, Papazoglu C, Wu Y, Capurso D, Brodt M, Francis D, et al. CHD5 is a tumor suppressor at human 1p36. Cell. 2007;128(3):459–75. 17289567 10.1016/j.cell.2006.11.052

[64] Watanabe S, Tan D, Lakshminarasimhan M, Washburn MP, Hong EJ, Walz T, et al. Structural analyses of the chromatin remodelling enzymes INO80-C and SWR-C. Nat Commun. 2015;6:7108. 25964121 10.1038/ncomms8108 PMC4431590

[65] Willhoft O, Wigley DB. INO80 and SWR1 complexes: the non-identical twins of chromatin remodelling. Curr Opin Struct Biol. 2020;61:50–8. 31838293 10.1016/j.sbi.2019.09.002 PMC7171469

[66] Gerhold CB, Gasser SM. INO80 and SWR complexes: relating structure to function in chromatin remodeling. Trends Cell Biol. 2014;24(11):619–31. 25088669 10.1016/j.tcb.2014.06.004

[67] Nguyen VQ, Ranjan A, Stengel F, Wei D, Aebersold R, Wu C, et al. Molecular architecture of the ATP-dependent chromatin-remodeling complex SWR1. Cell. 2013;154(6):1220–31. 24034246 10.1016/j.cell.2013.08.018 PMC3776929

[68] Tosi A, Haas C, Herzog F, Gilmozzi A, Berninghausen O, Ungewickell C, et al. Structure and subunit topology of the INO80 chromatin remodeler and its nucleosome complex. Cell. 2013;154(6):1207–19. 24034245 10.1016/j.cell.2013.08.016

[69] Liu M, Xu Q, Wang H, Ji X. INO80/SWR remodelers regulate Pol II transcription through BRD2 and chromatin landscape. Nucleic Acids Res. 2025;53(17):gkaf892. 40966518 10.1093/nar/gkaf892 PMC12448918

[70] Mizuguchi G, Shen X, Landry J, Wu WH, Sen S, Wu C. ATP-driven exchange of histone H2AZ variant catalyzed by SWR1 chromatin remodeling complex. Science. 2004;303(5656):343–8. 14645854 10.1126/science.1090701

[71] Luk E, Ranjan A, Fitzgerald PC, Mizuguchi G, Huang Y, Wei D, et al. Stepwise histone replacement by SWR1 requires dual activation with histone H2A.Z and canonical nucleosome. Cell. 2010;143(5):725–36. 21111233 10.1016/j.cell.2010.10.019 PMC7251641

[72] Li S, Wei T, Panchenko AR. Histone variant H2A.Z modulates nucleosome dynamics to promote DNA accessibility. Nat Commun. 2023;14(1):769. 36765119 10.1038/s41467-023-36465-5 PMC9918499

[73] Krogan NJ, Keogh MC, Datta N, Sawa C, Ryan OW, Ding H, et al. A Snf2 family ATPase complex required for recruitment of the histone H2A variant Htz1. Mol Cell. 2003;12(6):1565–76. 14690608 10.1016/s1097-2765(03)00497-0

[74] Prendergast L, McClurg UL, Hristova R, Berlinguer-Palmini R, Greener S, Veitch K, et al. Resolution of R-loops by INO80 promotes DNA replication and maintains cancer cell proliferation and viability. Nat Commun. 2020;11(1):4534. 32913330 10.1038/s41467-020-18306-x PMC7484789

[75] Zhang S, Zhou B, Wang L, Li P, Bennett BD, Snyder R, et al. INO80 is required for oncogenic transcription and tumor growth in non-small cell lung cancer. Oncogene. 2017;36(10):1430–9. 27641337 10.1038/onc.2016.311 PMC6534818

[76] Mach P, Kos PI, Zhan Y, Cramard J, Gaudin S, Tünnermann J, et al. Cohesin and CTCF control the dynamics of chromosome folding. Nat Genet. 2022;54(12):1907–18. 36471076 10.1038/s41588-022-01232-7 PMC9729113

[77] Davidson IF, Barth R, Zaczek M, van der Torre J, Tang W, Nagasaka K, et al. CTCF is a DNA-tension-dependent barrier to cohesin-mediated loop extrusion. Nature. 2023;616(7958):822–7. 37076620 10.1038/s41586-023-05961-5 PMC10132984

[78] Yuan X, Yan L, Chen Q, Zhu S, Zhou X, Zeng LH, et al. Molecular mechanism and functional significance of Wapl interaction with the Cohesin complex. Proc Natl Acad Sci U S A. 2024;121(33):e2405177121. 39110738 10.1073/pnas.2405177121 PMC11331136

[79] Ochs F, Green C, Szczurek AT, Pytowski L, Kolesnikova S, Brown J, et al. Sister chromatid cohesion is mediated by individual cohesin complexes. Science. 2024;383(6687):1122–30. 38452070 10.1126/science.adl4606

[80] Losada A. Cohesin in cancer: chromosome segregation and beyond. Nat Rev Cancer. 2014;14(6):389–93. 24854081 10.1038/nrc3743

[81] Waldman T. Emerging themes in cohesin cancer biology. Nat Rev Cancer. 2020;20(9):504–15. 32514055 10.1038/s41568-020-0270-1

[82] Su XA, Stopsack KH, Schmidt DR, Ma D, Li Z, Scheet PA, et al. RAD21 promotes oncogenesis and lethal progression of prostate cancer. Proc Natl Acad Sci U S A. 2024;121(36):e2405543121. 39190349 10.1073/pnas.2405543121 PMC11388324

[83] Xia X, Liu X, Li T, Fang X, Chen Z. Structure of chromatin remodeler Swi2/Snf2 in the resting state. Nat Struct Mol Biol. 2016;23(8):722–9. 27399259 10.1038/nsmb.3259

[84] Li M, Xia X, Tian Y, Jia Q, Liu X, Lu Y, et al. Mechanism of DNA translocation underlying chromatin remodelling by Snf2. Nature. 2019;567(7748):409–13. 30867599 10.1038/s41586-019-1029-2

[85] Nodelman IM, Das S, Faustino AM, Fried SD, Bowman GD, Armache JP. Nucleosome recognition and DNA distortion by the Chd1 remodeler in a nucleotide-free state. Nat Struct Mol Biol. 2022;29(2):121–9. 35173352 10.1038/s41594-021-00719-x PMC9107065

[86] Clapier CR, Iwasa J, Cairns BR, Peterson CL. Mechanisms of action and regulation of ATP-dependent chromatin-remodelling complexes. Nat Rev Mol Cell Biol. 2017;18(7):407–22. 28512350 10.1038/nrm.2017.26 PMC8127953

[87] Brandani GB, Niina T, Tan C, Takada S. DNA sliding in nucleosomes via twist defect propagation revealed by molecular simulations. Nucleic Acids Res. 2018;46(6):2788–801. 29506273 10.1093/nar/gky158 PMC5887990

[88] Eustermann S, Patel AB, Hopfner KP, He Y, Korber P. Energy-driven genome regulation by ATP-dependent chromatin remodellers. Nat Rev Mol Cell Biol. 2024;25(4):309–32. 38081975 10.1038/s41580-023-00683-y PMC12303036

[89] Clapier CR, Kasten MM, Parnell TJ, Viswanathan R, Szerlong H, Sirinakis G, et al. Regulation of DNA Translocation Efficiency within the Chromatin Remodeler RSC/Sth1 Potentiates Nucleosome Sliding and Ejection. Mol Cell. 2016;62(3):453–61. 27153540 10.1016/j.molcel.2016.03.032 PMC5291166

[90] Dann GP, Liszczak GP, Bagert JD, Müller MM, Nguyen UTT, Wojcik F, et al. ISWI chromatin remodellers sense nucleosome modifications to determine substrate preference. Nature. 2017;548(7669):607–11. 28767641 10.1038/nature23671 PMC5777669

[91] Klemm SL, Shipony Z, Greenleaf WJ. Chromatin accessibility and the regulatory epigenome. Nat Rev Genet. 2019;20(4):207–20. 30675018 10.1038/s41576-018-0089-8

[92] Rao SS, Huntley MH, Durand NC, Stamenova EK, Bochkov ID, Robinson JT, et al. A 3D map of the human genome at kilobase resolution reveals principles of chromatin looping. Cell. 2014;159(7):1665–80. 25497547 10.1016/j.cell.2014.11.021 PMC5635824

[93] Li Y, Haarhuis JHI, Sedeño Cacciatore Á, Oldenkamp R, van Ruiten MS, Willems L, et al. The structural basis for cohesin-CTCF-anchored loops. Nature. 2020;578(7795):472–6. 31905366 10.1038/s41586-019-1910-z PMC7035113

[94] Sabaté T, Lelandais B, Robert MC, Szalay M, Tinevez JY, Bertrand E, et al. Uniform dynamics of cohesin-mediated loop extrusion in living human cells. Nat Genet. 2025;57(12):3152–64. 41238959 10.1038/s41588-025-02406-9 PMC12695666

[95] Zuin J, Dixon JR, van der Reijden MI, Ye Z, Kolovos P, Brouwer RW, et al. Cohesin and CTCF differentially affect chromatin architecture and gene expression in human cells. Proc Natl Acad Sci U S A. 2014;111(3):996–1001. 24335803 10.1073/pnas.1317788111 PMC3903193

[96] Fudenberg G, Imakaev M, Lu C, Goloborodko A, Abdennur N, Mirny LA. Formation of Chromosomal Domains by Loop Extrusion. Cell Rep. 2016;15(9):2038–49. 27210764 10.1016/j.celrep.2016.04.085 PMC4889513

[97] Rowley MJ, Corces VG. Organizational principles of 3D genome architecture. Nat Rev Genet. 2018;19(12):789–800. 30367165 10.1038/s41576-018-0060-8 PMC6312108

[98] Wutz G, Varnai C, Nagasaka K, Cisneros DA, Stocsits RR, Tang W, et al. Topologically associating domains and chromatin loops depend on cohesin and are regulated by CTCF, WAPL, and PDS5 proteins. EMBO J. 2017;36(24):3573–99. 29217591 10.15252/embj.201798004 PMC5730888

[99] Rao SSP, Huang SC, Glenn St Hilaire B, Engreitz JM, Perez EM, Kieffer-Kwon KR, et al. Cohesin Loss Eliminates All Loop Domains. Cell. 2017;171(2):305–20.e24 10.1016/j.cell.2017.09.026 PMC5846482 28985562

[100] Shang XY, Shi Y, He DD, Wang L, Luo Q, Deng CH, et al. ARID1A deficiency weakens BRG1-RAD21 interaction that jeopardizes chromatin compactness and drives liver cancer cell metastasis. Cell Death Dis. 2021;12(11):990. 34689165 10.1038/s41419-021-04291-6 PMC8542038

[101] Hakimi MA, Bochar DA, Schmiesing JA, Dong Y, Barak OG, Speicher DW, et al. A chromatin remodelling complex that loads cohesin onto human chromosomes. Nature. 2002;418(6901):994–8. 12198550 10.1038/nature01024

[102] Iurlaro M, Masoni F, Flyamer IM, Wirbelauer C, Iskar M, Burger L, et al. Systematic assessment of ISWI subunits shows that NURF creates local accessibility for CTCF. Nat Genet. 2024;56(6):1203–12. 38816647 10.1038/s41588-024-01767-x PMC11176080

[103] Niu L, Shen W, Shi Z, Tan Y, He N, Wan J, et al. Three-dimensional folding dynamics of the Xenopus tropicalis genome. Nat Genet. 2021;53(7):1075–87. 34099928 10.1038/s41588-021-00878-z PMC8270788

[104] Barisic D, Stadler MB, Iurlaro M, Schübeler D. Mammalian ISWI and SWI/SNF selectively mediate binding of distinct transcription factors. Nature. 2019;569(7754):136–40. 30996347 10.1038/s41586-019-1115-5 PMC6522387

[105] Muñoz S, Jones A, Bouchoux C, Gilmore T, Patel H, Uhlmann F. Functional crosstalk between the cohesin loader and chromatin remodelers. Nat Commun. 2022;13(1):7698. 36509793 10.1038/s41467-022-35444-6 PMC9744909

[106] Sahu RK, Dhakshnamoorthy J, Jain S, Folco HD, Wheeler D, Grewal SIS. Nucleosome remodeler exclusion by histone deacetylation enforces heterochromatic silencing and epigenetic inheritance. Mol Cell. 2024;84(17):3175-91.e8. 39096900 10.1016/j.molcel.2024.07.006 PMC11649001

[107] Chen L, Maristany MJ, Farr SE, Luo J, Gibson BA, Doolittle LK, et al. Nucleosome spacing can fine-tune higher-order chromatin assembly. Nat Commun. 2025;16(1):6315. 40628730 10.1038/s41467-025-61482-x PMC12238351

[108] Almassalha LM, Carignano M, Liwag EP, Li WS, Gong R, Acosta N, et al. Chromatin conformation, gene transcription and nucleosome remodeling as an emergent system. Sci Adv. 2025;11(2):eadq6652. 10.1126/sciadv.adq6652 PMC11721585 39792661

[109] Hoencamp C, Rowland BD. Genome control by SMC complexes. Nat Rev Mol Cell Biol. 2023;24(9):633–50. 37231112 10.1038/s41580-023-00609-8

[110] Kubo N, Ishii H, Xiong X, Bianco S, Meitinger F, Hu R, et al. Promoter-proximal CTCF binding promotes distal enhancer-dependent gene activation. Nat Struct Mol Biol. 2021;28(2):152–61. 33398174 10.1038/s41594-020-00539-5 PMC7913465

[111] Badis G, Chan ET, van Bakel H, Pena-Castillo L, Tillo D, Tsui K, et al. A library of yeast transcription factor motifs reveals a widespread function for Rsc3 in targeting nucleosome exclusion at promoters. Mol Cell. 2008;32(6):878–87. 19111667 10.1016/j.molcel.2008.11.020 PMC2743730

[112] Nguyen VQ, Ranjan A, Liu S, Tang X, Ling YH, Wisniewski J, et al. Spatiotemporal coordination of transcription preinitiation complex assembly in live cells. Mol Cell. 2021;81(17):3560-75.e6. 34375585 10.1016/j.molcel.2021.07.022 PMC8420877

[113] Whyte WA, Orlando DA, Hnisz D, Abraham BJ, Lin CY, Kagey MH, et al. Master transcription factors and mediator establish super-enhancers at key cell identity genes. Cell. 2013;153(2):307–19. 23582322 10.1016/j.cell.2013.03.035 PMC3653129

[114] Lovén J, Hoke HA, Lin CY, Lau A, Orlando DA, Vakoc CR, et al. Selective inhibition of tumor oncogenes by disruption of super-enhancers. Cell. 2013;153(2):320–34. 23582323 10.1016/j.cell.2013.03.036 PMC3760967

[115] Alver BH, Kim KH, Lu P, Wang X, Manchester HE, Wang W, et al. The SWI/SNF chromatin remodelling complex is required for maintenance of lineage specific enhancers. Nat Commun. 2017;8:14648. 28262751 10.1038/ncomms14648 PMC5343482

[116] Huang J, Li K, Cai W, Liu X, Zhang Y, Orkin SH, et al. Dissecting super-enhancer hierarchy based on chromatin interactions. Nat Commun. 2018;9(1):943. 29507293 10.1038/s41467-018-03279-9 PMC5838163

[117] White SM, Snyder MP, Yi C. Master lineage transcription factors anchor trans mega transcriptional complexes at highly accessible enhancer sites to promote long-range chromatin clustering and transcription of distal target genes. Nucleic Acids Res. 2021;49(21):12196–210. 34850122 10.1093/nar/gkab1105 PMC8643643

[118] Gong Y, Lazaris C, Sakellaropoulos T, Lozano A, Kambadur P, Ntziachristos P, et al. Stratification of TAD boundaries reveals preferential insulation of super-enhancers by strong boundaries. Nat Commun. 2018;9(1):542. 29416042 10.1038/s41467-018-03017-1 PMC5803259

[119] Weischenfeldt J, Dubash T, Drainas AP, Mardin BR, Chen Y, Stütz AM, et al. Pan-cancer analysis of somatic copy-number alterations implicates IRS4 and IGF2 in enhancer hijacking. Nat Genet. 2017;49(1):65–74. 27869826 10.1038/ng.3722 PMC5791882

[120] Jeon HY, Khadka S, Pornour M, Ryu H, Chen H, Hussain A, et al. BPTF regulates androgen receptor activity by enhancing chromatin accessibility and stabilizing the AR-FOXA1 interaction. Nat Commun. 2025;17(1):670. 41381516 10.1038/s41467-025-67329-9 PMC12820202

[121] Orlando KA, Grimm SA, Wade PA. Pioneering new enhancers by GATA3: role of facilitating transcription factors and chromatin remodeling. Nucleic Acids Res. 2025;53(11):gkaf473. 40479714 10.1093/nar/gkaf473 PMC12143596

[122] Chan YS, Gao Q, Robinson SA, Wang W, Filandrova R, Weinhold LM, et al. β-catenin functions as a molecular adapter for disordered cBAF interactions. Mol Cell. 2025;85(16):3041-56.e9. 40695292 10.1016/j.molcel.2025.06.026 PMC12323811

[123] Ahmad K, Brahma S, Henikoff S. Epigenetic pioneering by SWI/SNF family remodelers. Mol Cell. 2024;84(2):194–201. 38016477 10.1016/j.molcel.2023.10.045 PMC10842064

[124] Saha D, Animireddy S, Lee J, Thommen A, Murvin MM, Lu Y, et al. Enhancer switching in cell lineage priming is linked to eRNA, Brg1’s AT-hook, and SWI/SNF recruitment. Mol Cell. 2024;84(10):1855-69.e5. 38593804 10.1016/j.molcel.2024.03.013 PMC11104297

[125] Brahma S, Henikoff S. The BAF chromatin remodeler synergizes with RNA polymerase II and transcription factors to evict nucleosomes. Nat Genet. 2024;56(1):100–11. 38049663 10.1038/s41588-023-01603-8 PMC10786724

[126] Shan L, Zhou X, Liu X, Wang Y, Su D, Hou Y, et al. FOXK2 Elicits Massive Transcription Repression and Suppresses the Hypoxic Response and Breast Cancer Carcinogenesis. Cancer Cell. 2016;30(5):708–22. 27773593 10.1016/j.ccell.2016.09.010

[127] Kagey MH, Newman JJ, Bilodeau S, Zhan Y, Orlando DA, van Berkum NL, et al. Mediator and cohesin connect gene expression and chromatin architecture. Nature. 2010;467(7314):430–5. 20720539 10.1038/nature09380 PMC2953795

[128] Phillips-Cremins JE, Sauria ME, Sanyal A, Gerasimova TI, Lajoie BR, Bell JS, et al. Architectural protein subclasses shape 3D organization of genomes during lineage commitment. Cell. 2013;153(6):1281–95. 23706625 10.1016/j.cell.2013.04.053 PMC3712340

[129] Racko D, Benedetti F, Dorier J, Stasiak A. Transcription-induced supercoiling as the driving force of chromatin loop extrusion during formation of TADs in interphase chromosomes. Nucleic Acids Res. 2018;46(4):1648–60. 29140466 10.1093/nar/gkx1123 PMC5829651

[130] Deng P, Wang Z, Chen J, Liu S, Yao X, Liu S, et al. RAD21 amplification epigenetically suppresses interferon signaling to promote immune evasion in ovarian cancer. J Clin Invest. 2022;132(22):e159628. 36201246 10.1172/JCI159628 PMC9663158

[131] Beishline K, Vladimirova O, Tutton S, Wang Z, Deng Z, Lieberman PM. CTCF driven TERRA transcription facilitates completion of telomere DNA replication. Nat Commun. 2017;8(1):2114. 29235471 10.1038/s41467-017-02212-w PMC5727389

[132] Bannister AJ, Kouzarides T. Regulation of chromatin by histone modifications. Cell Res. 2011;21(3):381–95. 21321607 10.1038/cr.2011.22 PMC3193420

[133] Kouzarides T. Chromatin modifications and their function. Cell. 2007;128(4):693–705. 17320507 10.1016/j.cell.2007.02.005

[134] Fuchs HA, Peng Y, Ayyapan S, Rosas R, Zhang H, Panchenko AR, et al. G34R cancer mutation alters the conformational ensemble and dynamics of the histone H3.3 tails. Nucleic Acids Res. 2026;54(2):gkaf1381. 41533587 10.1093/nar/gkaf1381 PMC12802913

[135] Engl W, Kunstar-Thomas A, Chen S, Ng WS, Sielaff H, Zhao ZW. Single-molecule imaging of SWI/SNF chromatin remodelers reveals bromodomain-mediated and cancer-mutants-specific landscape of multi-modal DNA-binding dynamics. Nat Commun. 2024;15(1):7646. 39223123 10.1038/s41467-024-52040-y PMC11369179

[136] Xiong X, Panchenko T, Yang S, Zhao S, Yan P, Zhang W, et al. Selective recognition of histone crotonylation by double PHD fingers of MOZ and DPF2. Nat Chem Biol. 2016;12(12):1111–8. 27775714 10.1038/nchembio.2218 PMC5253430

[137] Hyun K, Ahn J, Kim H, Kim J, Kim YI, Park HS, et al. The BAF complex enhances transcription through interaction with H3K56ac in the histone globular domain. Nat Commun. 2024;15(1):9614. 39511190 10.1038/s41467-024-53981-0 PMC11544104

[138] Ryu HY, Hochstrasser M. Histone sumoylation and chromatin dynamics. Nucleic Acids Res. 2021;49(11):6043–52. 33885816 10.1093/nar/gkab280 PMC8216275

[139] Roesley SNA, La Marca JE, Deans AJ, McKenzie L, Suryadinata R, Burke P, et al. Phosphorylation of Drosophila Brahma on CDK-phosphorylation sites is important for cell cycle regulation and differentiation. Cell Cycle. 2018;17(13):1559–78. 29963966 10.1080/15384101.2018.1493414 PMC6133315

[140] Kim B, Luo Y, Zhan X, Zhang Z, Shi X, Yi J, et al. Neuronal activity-induced BRG1 phosphorylation regulates enhancer activation. Cell Rep. 2021;36(2):109357. 34260936 10.1016/j.celrep.2021.109357 PMC8315893

[141] Li W, Shan B, Zou C, Wang H, Zhang MM, Zhu H, et al. Nuclear RIPK1 promotes chromatin remodeling to mediate inflammatory response. Cell Res. 2022;32(7):621–37. 35661830 10.1038/s41422-022-00673-3 PMC9253060

[142] Wang L, Zhao Z, Meyer MB, Saha S, Yu M, Guo A, et al. CARM1 methylates chromatin remodeling factor BAF155 to enhance tumor progression and metastasis. Cancer Cell. 2014;25(1):21–36. 24434208 10.1016/j.ccr.2013.12.007 PMC4004525

[143] Radko-Juettner S, Yue H, Myers JA, Carter RD, Robertson AN, Mittal P, et al. Targeting DCAF5 suppresses SMARCB1-mutant cancer by stabilizing SWI/SNF. Nature. 2024;628(8007):442–9. 38538798 10.1038/s41586-024-07250-1 PMC11184678

[144] Guo P, Hoang N, Sanchez J, Zhang EH, Rajawasam K, Trinidad K, et al. The assembly of mammalian SWI/SNF chromatin remodeling complexes is regulated by lysine-methylation dependent proteolysis. Nat Commun. 2022;13(1):6696. 36335117 10.1038/s41467-022-34348-9 PMC9637158

[145] Tang X, Zeng P, Liu K, Qing L, Sun Y, Liu X, et al. The PTM profiling of CTCF reveals the regulation of 3D chromatin structure by O-GlcNAcylation. Nat Commun. 2024;15(1):2813. 38561336 10.1038/s41467-024-47048-3 PMC10985093

[146] Davis RB, Supakar A, Ranganath AK, Moosa MM, Banerjee PR. Heterotypic interactions can drive selective co-condensation of prion-like low-complexity domains of FET proteins and mammalian SWI/SNF complex. Nat Commun. 2024;15(1):1168. 38326345 10.1038/s41467-024-44945-5 PMC10850361

[147] Chambers C, Cermakova K, Chan YS, Kurtz K, Wohlan K, Lewis AH, et al. SWI/SNF blockade disrupts PU.1-directed enhancer programs in normal hematopoietic cells and acute myeloid leukemia. Cancer Res. 2023;83(7):983–96. 36662812 10.1158/0008-5472.CAN-22-2129 PMC10071820

[148] Barisic D, Chin CR, Meydan C, Teater M, Tsialta I, Mlynarczyk C, et al. ARID1A orchestrates SWI/SNF-mediated sequential binding of transcription factors with ARID1A loss driving pre-memory B cell fate and lymphomagenesis. Cancer Cell. 2024;42(4):583-604.e11. 38458187 10.1016/j.ccell.2024.02.010 PMC11407687

[149] Laubscher D, Gryder BE, Sunkel BD, Andresson T, Wachtel M, Das S, et al. BAF complexes drive proliferation and block myogenic differentiation in fusion-positive rhabdomyosarcoma. Nat Commun. 2021;12(1):6924. 34836971 10.1038/s41467-021-27176-w PMC8626462

[150] Rago F, Elliott G, Li A, Sprouffske K, Kerr G, Desplat A, et al. The Discovery of SWI/SNF Chromatin Remodeling Activity as a Novel and Targetable Dependency in Uveal Melanoma. Mol Cancer Ther. 2020;19(10):2186–95. 32747420 10.1158/1535-7163.MCT-19-1013

[151] Xu M, Hong JJ, Zhang X, Sun M, Liu X, Kang J, et al. Targeting SWI/SNF ATPases reduces neuroblastoma cell plasticity. Embo j. 2024;43(20):4522–41. 39174852 10.1038/s44318-024-00206-1 PMC11480351

[152] Cermakova K, Tao L, Dejmek M, Sala M, Montierth MD, Chan YS, et al. Reactivation of the G1 enhancer landscape underlies core circuitry addiction to SWI/SNF. Nucleic Acids Res. 2024;52(1):4–21. 37993417 10.1093/nar/gkad1081 PMC10783513

[153] Scully R, Panday A, Elango R, Willis NA. DNA double-strand break repair-pathway choice in somatic mammalian cells. Nat Rev Mol Cell Biol. 2019;20(11):698–714. 31263220 10.1038/s41580-019-0152-0 PMC7315405

[154] Watanabe R, Ui A, Kanno S, Ogiwara H, Nagase T, Kohno T, et al. SWI/SNF factors required for cellular resistance to DNA damage include ARID1A and ARID1B and show interdependent protein stability. Cancer Res. 2014;74(9):2465–75. 24788099 10.1158/0008-5472.CAN-13-3608

[155] Erdel F, Schubert T, Marth C, Längst G, Rippe K. Human ISWI chromatin-remodeling complexes sample nucleosomes via transient binding reactions and become immobilized at active sites. Proc Natl Acad Sci U S A. 2010;107(46):19873–8. 20974961 10.1073/pnas.1003438107 PMC2993390

[156] Ogiwara H, Ui A, Otsuka A, Satoh H, Yokomi I, Nakajima S, et al. Histone acetylation by CBP and p300 at double-strand break sites facilitates SWI/SNF chromatin remodeling and the recruitment of non-homologous end joining factors. Oncogene. 2011;30(18):2135–46. 21217779 10.1038/onc.2010.592

[157] Shen J, Peng Y, Wei L, Zhang W, Yang L, Lan L, et al. ARID1A Deficiency Impairs the DNA Damage Checkpoint and Sensitizes Cells to PARP Inhibitors. Cancer Discov. 2015;5(7):752–67. 26069190 10.1158/2159-8290.CD-14-0849 PMC4497871

[158] Hou T, Cao Z, Zhang J, Tang M, Tian Y, Li Y, et al. SIRT6 coordinates with CHD4 to promote chromatin relaxation and DNA repair. Nucleic Acids Res. 2020;48(6):2982–3000. 31970415 10.1093/nar/gkaa006 PMC7102973

[159] Moser SC, Khalizieva A, Roehsner J, Pottendorfer E, Kaptein ML, Ricci G, et al. NASP modulates histone turnover to drive PARP inhibitor resistance. Nature. 2025;645(8082):1071–80. 40804522 10.1038/s41586-025-09414-z

[160] Ahel D, Horejsí Z, Wiechens N, Polo SE, Garcia-Wilson E, Ahel I, et al. Poly(ADP-ribose)-dependent regulation of DNA repair by the chromatin remodeling enzyme ALC1. Science. 2009;325(5945):1240–3. 19661379 10.1126/science.1177321 PMC3443743

[161] Verma P, Zhou Y, Cao Z, Deraska PV, Deb M, Arai E, et al. ALC1 links chromatin accessibility to PARP inhibitor response in homologous recombination-deficient cells. Nat Cell Biol. 2021;23(2):160–71. 33462394 10.1038/s41556-020-00624-3 PMC7880902

[162] Fedkenheuer M, Shang Y, Jung S, Fedkenheuer K, Park S, Mazza D, et al. A dual role of Cohesin in DNA DSB repair. Nat Commun. 2025;16(1):843. 39833168 10.1038/s41467-025-56086-4 PMC11747280

[163] Arnould C, Rocher V, Finoux AL, Clouaire T, Li K, Zhou F, et al. Loop extrusion as a mechanism for formation of DNA damage repair foci. Nature. 2021;590(7847):660–5. 33597753 10.1038/s41586-021-03193-z PMC7116834

[164] Tittel-Elmer M, Lengronne A, Davidson MB, Bacal J, François P, Hohl M, et al. Cohesin association to replication sites depends on rad50 and promotes fork restart. Mol Cell. 2012;48(1):98–108. 22885006 10.1016/j.molcel.2012.07.004 PMC3904740

[165] Carvajal-Maldonado D, Byrum AK, Jackson J, Wessel S, Lemaçon D, Guitton-Sert L, et al. Perturbing cohesin dynamics drives MRE11 nuclease-dependent replication fork slowing. Nucleic Acids Res. 2019;47(3):1294–310. 29917110 10.1093/nar/gky519 PMC6379725

[166] O’Neil NJ, van Pel DM, Hieter P. Synthetic lethality and cancer: cohesin and PARP at the replication fork. Trends Genet. 2013;29(5):290–7. 23333522 10.1016/j.tig.2012.12.004 PMC3868440

[167] Park JH, Park EJ, Lee HS, Kim SJ, Hur SK, Imbalzano AN, et al. Mammalian SWI/SNF complexes facilitate DNA double-strand break repair by promoting gamma-H2AX induction. Embo j. 2006;25(17):3986–97. 16932743 10.1038/sj.emboj.7601291 PMC1560357

[168] Versteege I, Medjkane S, Rouillard D, Delattre O. A key role of the hSNF5/INI1 tumour suppressor in the control of the G1-S transition of the cell cycle. Oncogene. 2002;21(42):6403–12. 12226744 10.1038/sj.onc.1205841

[169] Wang X, Werneck MB, Wilson BG, Kim HJ, Kluk MJ, Thom CS, et al. TCR-dependent transformation of mature memory phenotype T cells in mice. J Clin Invest. 2011;121(10):3834–45. 21926465 10.1172/JCI37210 PMC3195451

[170] Piunti A, Shilatifard A. The roles of Polycomb repressive complexes in mammalian development and cancer. Nat Rev Mol Cell Biol. 2021;22(5):326–45. 33723438 10.1038/s41580-021-00341-1

[171] Wilson BG, Wang X, Shen X, McKenna ES, Lemieux ME, Cho YJ, et al. Epigenetic antagonism between polycomb and SWI/SNF complexes during oncogenic transformation. Cancer Cell. 2010;18(4):316–28. 20951942 10.1016/j.ccr.2010.09.006 PMC2957473

[172] Kim KH, Kim W, Howard TP, Vazquez F, Tsherniak A, Wu JN, et al. SWI/SNF-mutant cancers depend on catalytic and non-catalytic activity of EZH2. Nat Med. 2015;21(12):1491–6. 26552009 10.1038/nm.3968 PMC4886303

[173] Fisher JB, Peterson J, Reimer M, Stelloh C, Pulakanti K, Gerbec ZJ, et al. The cohesin subunit Rad21 is a negative regulator of hematopoietic self-renewal through epigenetic repression of Hoxa7 and Hoxa9. Leukemia. 2017;31(3):712–9. 27554164 10.1038/leu.2016.240 PMC5332284

[174] Adane B, Alexe G, Seong BKA, Lu D, Hwang EE, Hnisz D, et al. STAG2 loss rewires oncogenic and developmental programs to promote metastasis in Ewing sarcoma. Cancer Cell. 2021;39(6):827-44.e10. 34129824 10.1016/j.ccell.2021.05.007 PMC8378827

[175] Pavlova NN, Zhu J, Thompson CB. The hallmarks of cancer metabolism: Still emerging. Cell Metab. 2022;34(3):355–77. 35123658 10.1016/j.cmet.2022.01.007 PMC8891094

[176] Wu S, Fukumoto T, Lin J, Nacarelli T, Wang Y, Ong D, et al. Targeting glutamine dependence through GLS1 inhibition suppresses ARID1A-inactivated clear cell ovarian carcinoma. Nat Cancer. 2021;2(2):189–200. 34085048 10.1038/s43018-020-00160-x PMC8168620

[177] Tang Y, Jin YH, Li HL, Xin H, Chen JD, Li XY, et al. PBRM1 deficiency oncogenic addiction is associated with activated AKT-mTOR signalling and aerobic glycolysis in clear cell renal cell carcinoma cells. J Cell Mol Med. 2022;26(14):3837–49. 35672925 10.1111/jcmm.17418 PMC9279584

[178] Jing H, Zhang X, Meng L. Swinging the SWI/SNF complexes for cancer therapy. Trends Pharmacol Sci. 2025;46(9):907–21. 40813203 10.1016/j.tips.2025.07.008

[179] Lissanu Deribe Y, Sun Y, Terranova C, Khan F, Martinez-Ledesma J, Gay J, et al. Mutations in the SWI/SNF complex induce a targetable dependence on oxidative phosphorylation in lung cancer. Nat Med. 2018;24(7):1047–57. 29892061 10.1038/s41591-018-0019-5 PMC6650267

[180] Hu Y, Liu W, Fang W, Dong Y, Zhang H, Luo Q. Tumor energy metabolism: implications for therapeutic targets. Mol Biomed. 2024;5(1):63. 39609317 10.1186/s43556-024-00229-4 PMC11604893

[181] Qu YL, Deng CH, Luo Q, Shang XY, Wu JX, Shi Y, et al. Arid1a regulates insulin sensitivity and lipid metabolism. EBioMedicine. 2019;42:481–93. 30879920 10.1016/j.ebiom.2019.03.021 PMC6491943

[182] Zhang J, Gao Z, Yang Y, Li Z, Wu B, Fan C, et al. SNF2L maintains glutathione homeostasis by initiating SLC7A11 transcription through chromatin remodeling. Cell Death Dis. 2024;15(11):820. 39532848 10.1038/s41419-024-07221-4 PMC11557580

[183] Hanahan D. Hallmarks of cancer-Then and now, and beyond. Cell. 2026;189(8):2254-77. 41616779 10.1016/j.cell.2025.12.049

[184] Pardoll DM. The blockade of immune checkpoints in cancer immunotherapy. Nat Rev Cancer. 2012;12(4):252–64. 22437870 10.1038/nrc3239 PMC4856023

[185] Li J, Wang W, Zhang Y, Cieślik M, Guo J, Tan M, et al. Epigenetic driver mutations in ARID1A shape cancer immune phenotype and immunotherapy. J Clin Invest. 2020;130(5):2712–26. 32027624 10.1172/JCI134402 PMC7190935

[186] Mayes K, Alkhatib SG, Peterson K, Alhazmi A, Song C, Chan V, et al. BPTF Depletion Enhances T-cell-Mediated Antitumor Immunity. Cancer Res. 2016;76(21):6183–92. 27651309 10.1158/0008-5472.CAN-15-3125 PMC5093041

[187] Landry JW, Banerjee S, Taylor B, Aplan PD, Singer A, Wu C. Chromatin remodeling complex NURF regulates thymocyte maturation. Genes Dev. 2011;25(3):275–86. 21289071 10.1101/gad.2007311 PMC3034902

[188] Zhao D, Cai L, Lu X, Liang X, Li J, Chen P, et al. Chromatin Regulator CHD1 Remodels the Immunosuppressive Tumor Microenvironment in PTEN-Deficient Prostate Cancer. Cancer Discov. 2020;10(9):1374–87. 32385075 10.1158/2159-8290.CD-19-1352 PMC7483306

[189] Chu Z, Gu L, Hu Y, Zhang X, Li M, Chen J, et al. STAG2 regulates interferon signaling in melanoma via enhancer loop reprogramming. Nat Commun. 2022;13(1):1859. 35388001 10.1038/s41467-022-29541-9 PMC8986786

[190] Oreskovic E, Wheeler EC, Mengwasser KE, Fujimura E, Martin TD, Tothova Z, et al. Genetic analysis of cancer drivers reveals cohesin and CTCF as suppressors of PD-L1. Proc Natl Acad Sci U S A. 2022;119(7):e2120540119. 35149558 10.1073/pnas.2120540119 PMC8851563

[191] Maxwell MB, Hom-Tedla MS, Yi J, Li S, Rivera SA, Yu J, et al. ARID1A suppresses R-loop-mediated STING-type I interferon pathway activation of anti-tumor immunity. Cell. 2024;187(13):3390-408.e19. 38754421 10.1016/j.cell.2024.04.025 PMC11193641

[192] Sun X, Wang SC, Wei Y, Luo X, Jia Y, Li L, et al. Arid1a has context-dependent oncogenic and tumor suppressor functions in liver cancer. Cancer Cell. 2017;32(5):574-89 e6. 29136504 10.1016/j.ccell.2017.10.007 PMC5728182

[193] Bakr A, Della Corte G, Veselinov O, Kelekçi S, Chen MM, Lin YY, et al. ARID1A regulates DNA repair through chromatin organization and its deficiency triggers DNA damage-mediated anti-tumor immune response. Nucleic Acids Res. 2024;52(10):5698–719. 38587186 10.1093/nar/gkae233 PMC11162808

[194] Langer LF, Ward JM, Archer TK. Tumor suppressor SMARCB1 suppresses super-enhancers to govern hESC lineage determination. Elife. 2019;8:e45672. 31033435 10.7554/eLife.45672 PMC6538374

[195] Liu NQ, Paassen I, Custers L, Zeller P, Teunissen H, Ayyildiz D, et al. SMARCB1 loss activates patient-specific distal oncogenic enhancers in malignant rhabdoid tumors. Nat Commun. 2023;14(1):7762. 38040699 10.1038/s41467-023-43498-3 PMC10692191

[196] Hong SH, Son KH, Ha SY, Wee TI, Choi SK, Won JE, et al. Nucleoporin 210 serves a key scaffold for SMARCB1 in liver cancer. Cancer Res. 2021;81(2):356–70. 33239431 10.1158/0008-5472.CAN-20-0568

[197] Di Nardo M, Pallotta MM, Musio A. The multifaceted roles of cohesin in cancer. J Exp Clin Cancer Res. 2022;41(1):96. 35287703 10.1186/s13046-022-02321-5 PMC8919599

[198] Versteege I, Sévenet N, Lange J, Rousseau-Merck MF, Ambros P, Handgretinger R, et al. Truncating mutations of hSNF5/INI1 in aggressive paediatric cancer. Nature. 1998;394(6689):203–6. 9671307 10.1038/28212

[199] Clark J, Rocques PJ, Crew AJ, Gill S, Shipley J, Chan AM, et al. Identification of novel genes, SYT and SSX, involved in the t(X;18)(p11.2;q11.2) translocation found in human synovial sarcoma. Nat Genet. 1994;7(4):502–8. 7951320 10.1038/ng0894-502

[200] Kadoch C, Crabtree GR. Reversible disruption of mSWI/SNF (BAF) complexes by the SS18-SSX oncogenic fusion in synovial sarcoma. Cell. 2013;153(1):71–85. 23540691 10.1016/j.cell.2013.02.036 PMC3655887

[201] Puliga E, Corso S, Pietrantonio F, Giordano S. Microsatellite instability in Gastric Cancer: Between lights and shadows. Cancer Treat Rev. 2021;95:102175. 33721595 10.1016/j.ctrv.2021.102175

[202] Wang K, Kan J, Yuen ST, Shi ST, Chu KM, Law S, et al. Exome sequencing identifies frequent mutation of ARID1A in molecular subtypes of gastric cancer. Nat Genet. 2011;43(12):1219–23. 22037554 10.1038/ng.982

[203] Li S, Zhang W, Yang Q, Li S, Zhou X, Wang H, et al. Genomic landscape of paired primary and peritoneal metastatic lesions in gastric cancer highlights evolutionary dynamics and mutational drivers. J Adv Res. 2026;81:507–19. 40436141 10.1016/j.jare.2025.05.043 PMC12957787

[204] Coorens THH, Collord G, Jung H, Wang Y, Moore L, Hooks Y, et al. The somatic mutation landscape of normal gastric epithelium. Nature. 2025;640(8058):418–26. 40108450 10.1038/s41586-025-08708-6 PMC11981919

[205] He CY, Qiu MZ, Yang XH, Zhou DL, Ma JJ, Long YK, et al. Classification of gastric cancer by EBV status combined with molecular profiling predicts patient prognosis. Clin Transl Med. 2020;10(1):353–62. 32508039 10.1002/ctm2.32 PMC7240851

[206] Yamada H, Takeshima H, Fujiki R, Yamashita S, Sekine S, Ando T, et al. ARID1A loss-of-function induces CpG island methylator phenotype. Cancer Lett. 2022;532:215587. 35131383 10.1016/j.canlet.2022.215587

[207] Dong X, Song S, Li Y, Fan Y, Wang L, Wang R, et al. Loss of ARID1A activates mTOR signaling and SOX9 in gastric adenocarcinoma-rationale for targeting ARID1A deficiency. Gut. 2022;71(3):467–78. 33785559 10.1136/gutjnl-2020-322660 PMC9724309

[208] Lo YH, Kolahi KS, Du Y, Chang CY, Krokhotin A, Nair A, et al. A CRISPR/Cas9-Engineered ARID1A-Deficient Human Gastric Cancer Organoid Model Reveals Essential and Nonessential Modes of Oncogenic Transformation. Cancer Discov. 2021;11(6):1562–81. 33451982 10.1158/2159-8290.CD-20-1109 PMC8346515

[209] Loe AKH, Francis R, Seo J, Du L, Wang Y, Kim JE, et al. Uncovering the dosage-dependent roles of Arid1a in gastric tumorigenesis for combinatorial drug therapy. J Exp Med. 2021;218(6):e20200219. 33822841 10.1084/jem.20200219 PMC8034383

[210] Takeshima H, Niwa T, Takahashi T, Wakabayashi M, Yamashita S, Ando T, et al. Frequent involvement of chromatin remodeler alterations in gastric field cancerization. Cancer Lett. 2015;357(1):328–38. 25462860 10.1016/j.canlet.2014.11.038

[211] Kim JJ, Chung SW, Kim JH, Kim JW, Oh JS, Kim S, et al. Promoter methylation of helicase-like transcription factor is associated with the early stages of gastric cancer with family history. Ann Oncol. 2006;17(4):657–62. 16497821 10.1093/annonc/mdl018

[212] Yamamichi N, Inada K, Ichinose M, Yamamichi-Nishina M, Mizutani T, Watanabe H, et al. Frequent loss of Brm expression in gastric cancer correlates with histologic features and differentiation state. Cancer Res. 2007;67(22):10727–35. 18006815 10.1158/0008-5472.CAN-07-2601

[213] Yang Z, Zou S, Zhang Y, Zhang J, Zhang P, Xiao L, et al. ACTL6A protects gastric cancer cells against ferroptosis through induction of glutathione synthesis. Nat Commun. 2023;14(1):4193. 37443154 10.1038/s41467-023-39901-8 PMC10345109

[214] Fujimoto A, Totoki Y, Abe T, Boroevich KA, Hosoda F, Nguyen HH, et al. Whole-genome sequencing of liver cancers identifies etiological influences on mutation patterns and recurrent mutations in chromatin regulators. Nat Genet. 2012;44(7):760–4. 22634756 10.1038/ng.2291

[215] Guichard C, Amaddeo G, Imbeaud S, Ladeiro Y, Pelletier L, Maad IB, et al. Integrated analysis of somatic mutations and focal copy-number changes identifies key genes and pathways in hepatocellular carcinoma. Nat Genet. 2012;44(6):694–8. 22561517 10.1038/ng.2256 PMC3819251

[216] He F, Li J, Xu J, Zhang S, Xu Y, Zhao W, et al. Decreased expression of ARID1A associates with poor prognosis and promotes metastases of hepatocellular carcinoma. J Exp Clin Cancer Res. 2015;34(1):47. 25975202 10.1186/s13046-015-0164-3 PMC4440314

[217] Huang J, Deng Q, Wang Q, Li KY, Dai JH, Li N, et al. Exome sequencing of hepatitis B virus-associated hepatocellular carcinoma. Nat Genet. 2012;44(10):1117–21. 22922871 10.1038/ng.2391

[218] Li M, Zhao H, Zhang X, Wood LD, Anders RA, Choti MA, et al. Inactivating mutations of the chromatin remodeling gene ARID2 in hepatocellular carcinoma. Nat Genet. 2011;43(9):828–9. 21822264 10.1038/ng.903 PMC3163746

[219] Jiang H, Cao HJ, Ma N, Bao WD, Wang JJ, Chen TW, et al. Chromatin remodeling factor ARID2 suppresses hepatocellular carcinoma metastasis via DNMT1-Snail axis. Proc Natl Acad Sci U S A. 2020;117(9):4770–80. 32071245 10.1073/pnas.1914937117 PMC7060681

[220] Wang P, Song X, Cao D, Cui K, Wang J, Utpatel K, et al. Oncogene-dependent function of BRG1 in hepatocarcinogenesis. Cell Death Dis. 2020;11(2):91. 32019910 10.1038/s41419-020-2289-3 PMC7000409

[221] Hu C, Li W, Tian F, Jiang K, Liu X, Cen J, et al. Arid1a regulates response to anti-angiogenic therapy in advanced hepatocellular carcinoma. J Hepatol. 2018;68(3):465–75. 29113912 10.1016/j.jhep.2017.10.028

[222] Oba A, Shimada S, Akiyama Y, Nishikawaji T, Mogushi K, Ito H, et al. ARID2 modulates DNA damage response in human hepatocellular carcinoma cells. J Hepatol. 2017;66(5):942–51. 28238438 10.1016/j.jhep.2016.12.026

[223] Sun RQ, Ye YH, Xu Y, Wang B, Pan SY, Li N, et al. Integrated molecular characterization of sarcomatoid hepatocellular carcinoma. Clin Mol Hepatol. 2025;31(2):426–44. 39657751 10.3350/cmh.2024.0686 PMC12016616

[224] Fujita M, Yamaguchi R, Hasegawa T, Shimada S, Arihiro K, Hayashi S, et al. Classification of primary liver cancer with immunosuppression mechanisms and correlation with genomic alterations. EBioMedicine. 2020;53:102659. 32113157 10.1016/j.ebiom.2020.102659 PMC7048625

[225] Chen Z, Lu X, Jia D, Jing Y, Chen D, Wang Q, et al. Hepatic SMARCA4 predicts HCC recurrence and promotes tumour cell proliferation by regulating SMAD6 expression. Cell Death Dis. 2018;9(2):59. 29352111 10.1038/s41419-017-0090-8 PMC5833410

[226] Mizrahi JD, Surana R, Valle JW, Shroff RT. Pancreatic cancer. Lancet. 2020;395(10242):2008–20. 32593337 10.1016/S0140-6736(20)30974-0

[227] Yavas A, Ozcan K, Adsay NV, Balci S, Tarcan ZC, Hechtman JF, et al. SWI/SNF Complex-Deficient Undifferentiated Carcinoma of the Pancreas: Clinicopathologic and Genomic Analysis. Mod Pathol. 2024;37(11):100585. 39094734 10.1016/j.modpat.2024.100585 PMC11585460

[228] Zhang Z, Wang F, Du C, Guo H, Ma L, Liu X, et al. BRM/SMARCA2 promotes the proliferation and chemoresistance of pancreatic cancer cells by targeting JAK2/STAT3 signaling. Cancer Lett. 2017;402:213–24. 28602977 10.1016/j.canlet.2017.05.006

[229] Integrated Genomic Characterization of Pancreatic Ductal Adenocarcinoma. Cancer Cell. 2017;32(2):185-203.e13. 28810144 10.1016/j.ccell.2017.07.007 PMC5964983

[230] Zhang X, Mao T, Zhang B, Xu H, Cui J, Jiao F, et al. Characterization of the genomic landscape in large-scale Chinese patients with pancreatic cancer. EBioMedicine. 2022;77:103897. 35231699 10.1016/j.ebiom.2022.103897 PMC8886010

[231] Keane F, Chou JF, Walch H, Schoenfeld J, Singhal A, Cowzer D, et al. Precision medicine for pancreatic cancer: characterizing the clinicogenomic landscape and outcomes of KRAS G12C-mutated disease. J Natl Cancer Inst. 2024;116(9):1429–38. 38702822 10.1093/jnci/djae095 PMC11378314

[232] Araki O, Tsuda M, Omatsu M, Namikawa M, Sono M, Fukunaga Y, et al. Brg1 controls stemness and metastasis of pancreatic cancer through regulating hypoxia pathway. Oncogene. 2023;42(26):2139–52. 37198398 10.1038/s41388-023-02716-4

[233] Roy N, Malik S, Villanueva KE, Urano A, Lu X, Von Figura G, et al. Brg1 promotes both tumor-suppressive and oncogenic activities at distinct stages of pancreatic cancer formation. Genes Dev. 2015;29(6):658–71. 25792600 10.1101/gad.256628.114 PMC4378197

[234] Liu X, Tian X, Wang F, Ma Y, Kornmann M, Yang Y. BRG1 promotes chemoresistance of pancreatic cancer cells through crosstalking with Akt signalling. Eur J Cancer. 2014;50(13):2251–62. 24953335 10.1016/j.ejca.2014.05.017

[235] Lee CA, Abd-Rabbo D, Reimand J. Functional and genetic determinants of mutation rate variability in regulatory elements of cancer genomes. Genome Biol. 2021;22(1):133. 33941236 10.1186/s13059-021-02318-x PMC8091793

[236] Wang W, Friedland SC, Guo B, O’Dell MR, Alexander WB, Whitney-Miller CL, et al. ARID1A, a SWI/SNF subunit, is critical to acinar cell homeostasis and regeneration and is a barrier to transformation and epithelial-mesenchymal transition in the pancreas. Gut. 2019;68(7):1245–58. 30228219 10.1136/gutjnl-2017-315541 PMC6551318

[237] Kimura Y, Fukuda A, Ogawa S, Maruno T, Takada Y, Tsuda M, et al. ARID1A maintains differentiation of pancreatic ductal cells and inhibits development of pancreatic ductal adenocarcinoma in mice. Gastroenterology. 2018;155(1):194-209.e2. 29604291 10.1053/j.gastro.2018.03.039

[238] Wang SC, Nassour I, Xiao S, Zhang S, Luo X, Lee J, et al. SWI/SNF component ARID1A restrains pancreatic neoplasia formation. Gut. 2019;68(7):1259–70. 30315093 10.1136/gutjnl-2017-315490 PMC6499717

[239] Kawai M, Fukuda A, Ikeda M, Iimori K, Mizukoshi K, Iwane K, et al. Polybromo 1/vimentin axis dictates tumor grade, epithelial-mesenchymal transition, and metastasis in pancreatic cancer. J Clin Invest. 2025;135(11):e177533. 40454484 10.1172/JCI177533 PMC12126225

[240] Cuadrado A, Remeseiro S, Graña O, Pisano DG, Losada A. The contribution of cohesin-SA1 to gene expression and chromatin architecture in two murine tissues. Nucleic Acids Res. 2015;43(6):3056–67. 25735743 10.1093/nar/gkv144 PMC4381060

[241] Ma J, Cui Y, Cao T, Xu H, Shi Y, Xia J, et al. PDS5B regulates cell proliferation and motility via upregulation of Ptch2 in pancreatic cancer cells. Cancer Lett. 2019;460:65–74. 31233836 10.1016/j.canlet.2019.06.014

[242] Bray F, Laversanne M, Sung H, Ferlay J, Siegel RL, Soerjomataram I, et al. Global cancer statistics 2022: GLOBOCAN estimates of incidence and mortality worldwide for 36 cancers in 185 countries. CA Cancer J Clin. 2022;74(3):229–63. 10.3322/caac.21834 38572751

[243] Kim J, Bowlby R, Mungall AJ, Robertson AG, Odze RD, Cherniack AD, et al. Integrated genomic characterization of oesophageal carcinoma. Nature. 2017;541(7636):169–75. 28052061 10.1038/nature20805 PMC5651175

[244] Nakazato H, Takeshima H, Kishino T, Kubo E, Hattori N, Nakajima T, et al. Early-Stage Induction of SWI/SNF Mutations during Esophageal Squamous Cell Carcinogenesis. PLoS ONE. 2016;11(1):e0147372. 26812616 10.1371/journal.pone.0147372 PMC4728064

[245] Korpanty GJ, Eng L, Qiu X, Olusesan Faluyi O, Renouf DJ, Cheng D, et al. Association of BRM promoter polymorphisms and esophageal adenocarcinoma outcome. Oncotarget. 2017;8(17):28093-100. 28427211 10.18632/oncotarget.15890 PMC5438633

[246] Schallenberg S, Bork J, Essakly A, Alakus H, Buettner R, Hillmer AM, et al. Loss of the SWI/SNF-ATPase subunit members SMARCF1 (ARID1A), SMARCA2 (BRM), SMARCA4 (BRG1) and SMARCB1 (INI1) in oesophageal adenocarcinoma. BMC Cancer. 2020;20(1):12. 31906887 10.1186/s12885-019-6425-3 PMC6945480

[247] Luo Q, Wu X, Chang W, Zhao P, Nan Y, Zhu X, et al. ARID1A prevents squamous cell carcinoma initiation and chemoresistance by antagonizing pRb/E2F1/c-Myc-mediated cancer stemness. Cell Death Differ. 2020;27(6):1981–97. 31831874 10.1038/s41418-019-0475-6 PMC7244577

[248] Zhang L, Zheng Y, Chien W, Ziman B, Billet S, Koeffler HP, et al. ARID1A Deficiency Regulates Anti-Tumor Immune Response in Esophageal Adenocarcinoma. Cancers (Basel). 2023;15(22):5377. 38001638 10.3390/cancers15225377 PMC10670331

[249] Li Y, Yang X, Zhu W, Xu Y, Ma J, He C, et al. SWI/SNF complex gene variations are associated with a higher tumor mutational burden and a better response to immune checkpoint inhibitor treatment: a pan-cancer analysis of next-generation sequencing data corresponding to 4591 cases. Cancer Cell Int. 2022;22(1):347. 36371186 10.1186/s12935-022-02757-x PMC9652899

[250] Ehrenhöfer-Wölfer K, Puchner T, Schwarz C, Rippka J, Blaha-Ostermann S, Strobl U, et al. SMARCA2-deficiency confers sensitivity to targeted inhibition of SMARCA4 in esophageal squamous cell carcinoma cell lines. Sci Rep. 2019;9(1):11661. 31406271 10.1038/s41598-019-48152-x PMC6691015

[251] Zhou S, Sun T, Liu L, Guo D, Zhang X, Shen W, et al. The Knockdown of ACTL6A Enhances the Radiosensitivity of Esophageal Squamous Cell Carcinoma by Modulating the Wnt/β-Catenin Signaling Pathway. FASEB J. 2025;39(20):e71047. 41137713 10.1096/fj.202501681R PMC12553302

[252] Yasui K, Imoto I, Fukuda Y, Pimkhaokham A, Yang Z-Q, Naruto T, et al. Identification of target genes within an amplicon at 14q12-q13 in esophageal squamous cell carcinoma. Genes Chromosom Cancer. 2001;32(2):112–8. 11550278 10.1002/gcc.1172

[253] Lin Y, Zhao X, Du Z, Jia Z, Zhou S, Cao G, et al. MicroRNA miR-193b-3p Regulates Esophageal Cancer Progression Through Targeting RSF1. Cells. 2025;14(12):928. 40558555 10.3390/cells14120928 PMC12191309

[254] Liu C, Zou H, Ruan Y, Fang L, Wang B, Cui L, et al. Multiomics Reveals the Immunologic Features and the Immune Checkpoint Blockade Potential of Colorectal Medullary Carcinoma. Clin Cancer Res. 2025;31(4):773–86. 39651997 10.1158/1078-0432.CCR-24-2505 PMC11831109

[255] Johnson RM, Qu X, Lin CF, Huw LY, Venkatanarayan A, Sokol E, et al. ARID1A mutations confer intrinsic and acquired resistance to cetuximab treatment in colorectal cancer. Nat Commun. 2022;13(1):5478. 36117191 10.1038/s41467-022-33172-5 PMC9482920

[256] Mehrvarz Sarshekeh A, Alshenaifi J, Roszik J, Manyam GC, Advani SM, Katkhuda R, et al. ARID1A Mutation May Define an Immunologically Active Subgroup in Patients with Microsatellite Stable Colorectal Cancer. Clin Cancer Res. 2021;27(6):1663–70. 33414133 10.1158/1078-0432.CCR-20-2404 PMC7956157

[257] Wallner M, Herbst A, Behrens A, Crispin A, Stieber P, Göke B, et al. Methylation of serum DNA is an independent prognostic marker in colorectal cancer. Clin Cancer Res. 2006;12(24):7347–52. 17189406 10.1158/1078-0432.CCR-06-1264

[258] Leung WK, To KF, Man EP, Chan MW, Bai AH, Hui AJ, et al. Quantitative detection of promoter hypermethylation in multiple genes in the serum of patients with colorectal cancer. Am J Gastroenterol. 2005;100(10):2274–9. 16181380 10.1111/j.1572-0241.2005.50412.x

[259] Sarogni P, Palumbo O, Servadio A, Astigiano S, D’Alessio B, Gatti V, et al. Overexpression of the cohesin-core subunit SMC1A contributes to colorectal cancer development. J Exp Clin Cancer Res. 2019;38(1):108. 30823889 10.1186/s13046-019-1116-0 PMC6397456

[260] Sasaki M, Miyoshi N, Fujino S, Saso K, Ogino T, Takahashi H, et al. The meiosis-specific cohesin component stromal antigen 3 promotes cell migration and chemotherapeutic resistance in colorectal cancer. Cancer Lett. 2021;497:112–22. 33039558 10.1016/j.canlet.2020.10.006

[261] Chen JY, Ke TW, Chiang SF, Hong WZ, Chang HY, Liang JA, et al. CHK1 inhibition increases the therapeutic response to radiotherapy via antitumor immunity in ARID1A-deficient colorectal cancer. Cell Death Dis. 2025;16(1):584. 40750787 10.1038/s41419-025-07912-6 PMC12317038

[262] Hiramatsu Y, Fukuda A, Ogawa S, Goto N, Ikuta K, Tsuda M, et al. Arid1a is essential for intestinal stem cells through Sox9 regulation. Proc Natl Acad Sci U S A. 2019;116(5):1704–13. 30635419 10.1073/pnas.1804858116 PMC6358682

[263] Zeng X, Yao B, Liu J, Gong GW, Liu M, Li J, et al. The SMARCA4(R1157W) mutation facilitates chromatin remodeling and confers PRMT1/SMARCA4 inhibitors sensitivity in colorectal cancer. NPJ Precis Oncol. 2023;7(1):28. 36922568 10.1038/s41698-023-00367-y PMC10017700

[264] Yao B, Gui T, Zeng X, Deng Y, Wang Z, Wang Y, et al. PRMT1-mediated H4R3me2a recruits SMARCA4 to promote colorectal cancer progression by enhancing EGFR signaling. Genome Med. 2021;13(1):58. 33853662 10.1186/s13073-021-00871-5 PMC8048298

[265] Liu M, Sun T, Li N, Peng J, Fu D, Li W, et al. BRG1 attenuates colonic inflammation and tumorigenesis through autophagy-dependent oxidative stress sequestration. Nat Commun. 2019;10(1):4614. 31601814 10.1038/s41467-019-12573-z PMC6787222

[266] Wang G, Fu Y, Yang X, Luo X, Wang J, Gong J, et al. Brg-1 targeting of novel miR550a-5p/RNF43/Wnt signaling axis regulates colorectal cancer metastasis. Oncogene. 2016;35(5):651–61. 25961913 10.1038/onc.2015.124

[267] Wang G, Fu Y, Hu F, Lan J, Xu F, Yang X, et al. Loss of BRG1 induces CRC cell senescence by regulating p53/p21 pathway. Cell Death Dis. 2017;8(2):e2607. 28182012 10.1038/cddis.2017.1 PMC5386468

[268] Akano I, Hebert JM, Tiedemann RL, Gao Q, Xiao Y, Prescott NA, et al. The SWI/SNF-related protein SMARCA3 is a histone H3K23 ubiquitin ligase that regulates H3K9me3 in cancer. Mol Cell. 2025;85(15):2885-99 e8. 40680746 10.1016/j.molcel.2025.06.020 PMC12327810

[269] Yang Z, Huang D, Meng M, Wang W, Feng J, Fang L, et al. BAF53A drives colorectal cancer development by regulating DUSP5-mediated ERK phosphorylation. Cell Death Dis. 2022;13(12):1049. 36526622 10.1038/s41419-022-05499-w PMC9758165

[270] Yang HJ, Kim EJ, Kim Y, Cho Y, Choi Y, Song SH, et al. ACTL6A depletion induces KLF4-mediated anti-tumorigenic effects in colorectal cancer. Cell Death Dis. 2025;16(1):653. 40877226 10.1038/s41419-025-07946-w PMC12394641

[271] Wang H, Du S, Ji S, Miao G, Yu J, Zhou M, et al. SMARCA1-NPFF axis inhibits colorectal cancer metastasis by blocking epithelial-mesenchymal transition and macrophage-dependent immune reprogramming. Cancer Lett. 2025;631:217933. 40684839 10.1016/j.canlet.2025.217933

[272] Li D, Wang X, Miao H, Liu H, Pang M, Guo H, et al. The lncRNA MIR99AHG directs alternative splicing of SMARCA1 by PTBP1 to enable invadopodia formation in colorectal cancer cells. Sci Signal. 2023;16(803):eadh4210. 10.1126/scisignal.adh4210 37725664

[273] Zhou P, Xiao N, Wang J, Wang Z, Zheng S, Shan S, et al. SMC1A recruits tumor-associated-fibroblasts (TAFs) and promotes colorectal cancer metastasis. Cancer Lett. 2017;385:39–45. 27826041 10.1016/j.canlet.2016.10.041

[274] Siegel RL, Kratzer TB, Giaquinto AN, Sung H, Jemal A. Cancer statistics, 2025. CA Cancer J Clin. 2025;75(1):10–45. 39817679 10.3322/caac.21871 PMC11745215

[275] Caruso G, Weroha SJ, Cliby W. Ovarian Cancer: A Review. JAMA. 2025;334(14):1278–91. 40690248 10.1001/jama.2025.9495

[276] Li J, Han T, Guo D, Kong R, Chen S, Wang M, et al. Molecular profile of SWI/SNF complex in Chinese patients with ovarian cancer. Journal of Clinical Oncology. 2023;41(16_suppl):e17538-e.

[277] Banerjee S, Stewart J, Porta N, Toms C, Leary A, Lheureux S, et al. ATARI trial: ATR inhibitor in combination with olaparib in gynecological cancers with ARID1A loss or no loss (ENGOT/GYN1/NCRI). Int J Gynecol Cancer. 2021;31(11):1471–5. 34518240 10.1136/ijgc-2021-002973 PMC8573414

[278] Zhou W, Liu H, Yuan Z, Zundell J, Towers M, Lin J, et al. Targeting the mevalonate pathway suppresses ARID1A-inactivated cancers by promoting pyroptosis. Cancer Cell. 2023;41(4):740-56.e10. 36963401 10.1016/j.ccell.2023.03.002 PMC10085864

[279] Leitner K, Tsibulak I, Wieser V, Knoll K, Reimer D, Marth C, et al. Clinical impact of EZH2 and its antagonist SMARCA4 in ovarian cancer. Sci Rep. 2020;10(1):20412. 33230143 10.1038/s41598-020-77532-x PMC7684284

[280] Witkowski L, Carrot-Zhang J, Albrecht S, Fahiminiya S, Hamel N, Tomiak E, et al. Germline and somatic SMARCA4 mutations characterize small cell carcinoma of the ovary, hypercalcemic type. Nat Genet. 2014;46(5):438–43. 24658002 10.1038/ng.2931

[281] Jelinic P, Mueller JJ, Olvera N, Dao F, Scott SN, Shah R, et al. Recurrent SMARCA4 mutations in small cell carcinoma of the ovary. Nat Genet. 2014;46(5):424–6. 24658004 10.1038/ng.2922 PMC5699446

[282] Kurman RJ, International Agency for Research on C, World Health O. WHO classification of tumours of female reproductive organs. Fourth edition. Lyon, [France: International Agency for Research on Cancer; 2014.

[283] Leylek TR, Jeusset LM, Lichtensztejn Z, McManus KJ. Reduced Expression of Genes Regulating Cohesion Induces Chromosome Instability that May Promote Cancer and Impact Patient Outcomes. Sci Rep. 2020;10(1):592. 31953484 10.1038/s41598-020-57530-9 PMC6969069

[284] Gou R, Li X, Dong H, Hu Y, Liu O, Liu J, et al. RAD21 Confers Poor Prognosis and Affects Ovarian Cancer Sensitivity to Poly(ADP-Ribose)Polymerase Inhibitors Through DNA Damage Repair. Front Oncol. 2022;12:936550. 35860572 10.3389/fonc.2022.936550 PMC9289200

[285] Shih I-M, Sheu JJ-C, Santillan A, Nakayama K, Yen MJ, Bristow RE, et al. Amplification of a chromatin remodeling gene, Rsf-1/HBXAP, in ovarian carcinoma. Proc Natl Acad Sci U S A. 2005;102(39):14004–9. 16172393 10.1073/pnas.0504195102 PMC1236547

[286] Yang Y-I, Ahn J-H, Lee K-T, Shih I-M, Choi J-H. RSF1 Is a Positive Regulator of NF-κB–Induced Gene Expression Required for Ovarian Cancer Chemoresistance. Can Res. 2014;74(8):2258–69. 10.1158/0008-5472.CAN-13-2459 PMC4352305 24566868

[287] Choi JH, Sheu JJ-C, Guan B, Jinawath N, Markowski P, Wang T-L, et al. Functional analysis of 11q13.5 amplicon identifies Rsf-1 (HBXAP) as a gene involved in paclitaxel resistance in ovarian cancer. Cancer Res. 2009;69(4):1407–15. 19190325 10.1158/0008-5472.CAN-08-3602 PMC2644345

[288] Gorringe KL, Choong DY, Williams LH, Ramakrishna M, Sridhar A, Qiu W, et al. Mutation and methylation analysis of the chromodomain-helicase-DNA binding 5 gene in ovarian cancer. Neoplasia. 2008;10(11):1253–8. 18953434 10.1593/neo.08718 PMC2570601

[289] Wong RR, Chan LK, Tsang TP, Lee CW, Cheung TH, Yim SF, et al. CHD5 Downregulation Associated with Poor Prognosis in Epithelial Ovarian Cancer. Gynecol Obstet Invest. 2011;72(3):203–7. 21860208 10.1159/000323883

[290] Guillemette S, Serra RW, Peng M, Hayes JA, Konstantinopoulos PA, Green MR, et al. Resistance to therapy in BRCA2 mutant cells due to loss of the nucleosome remodeling factor CHD4. Genes Dev. 2015;29(5):489–94. 25737278 10.1101/gad.256214.114 PMC4358401

[291] Chen G, Li W, Guo J, Liu L, Wang Y. Development and Validation of Prognostic Characteristics Associated With Chromatin Remodeling-Related Genes in Ovarian Cancer. Cancer Med. 2025;14(3):e70634. 39932052 10.1002/cam4.70634 PMC11811884

[292] Fonteyne V, Tree A, Castro E, Touijer K, Walz J. Prostate cancer. Lancet. 2026;407(10528):622–36. 41418797 10.1016/S0140-6736(25)02221-4

[293] Cyrta J, Augspach A, De Filippo MR, Prandi D, Thienger P, Benelli M, et al. Role of specialized composition of SWI/SNF complexes in prostate cancer lineage plasticity. Nat Commun. 2020;11(1):5549. 33144576 10.1038/s41467-020-19328-1 PMC7642293

[294] Gokbayrak B, Altintas UB, Lingadahalli S, Morova T, Huang C-CF, Ersoy Fazlioglu B, et al. Identification of selective SWI/SNF dependencies in enzalutamide-resistant prostate cancer. Communications Biology. 2025;8(1):169. 10.1038/s42003-024-07413-w PMC11794516 39905188

[295] Xiao L, Parolia A, Qiao Y, Bawa P, Eyunni S, Mannan R, et al. Targeting SWI/SNF ATPases in enhancer-addicted prostate cancer. Nature. 2022;601(7893):434–9. 34937944 10.1038/s41586-021-04246-z PMC8770127

[296] Saha B, Arase A, Imam SS, Tsao-Wei D, Naritoku WY, Groshen S, et al. Overexpression of E-cadherin and β-Catenin proteins in metastatic prostate cancer cells in bone. Prostate. 2008;68(1):78–84. 18008331 10.1002/pros.20670

[297] Zhang Z, Zhou C, Li X, Barnes SD, Deng S, Hoover E, et al. Loss of <em>CHD1</em> Promotes Heterogeneous Mechanisms of Resistance to AR-Targeted Therapy via Chromatin Dysregulation. Cancer Cell. 2020;37(4):584-98.e11. 32220301 10.1016/j.ccell.2020.03.001 PMC7292228

[298] Calagua C, Ficial M, Jansen CS, Hirz T, Del Balzo L, Wilkinson S, et al. A Subset of Localized Prostate Cancer Displays an Immunogenic Phenotype Associated with Losses of Key Tumor Suppressor Genes. Clin Cancer Res. 2021;27(17):4836–47. 34168052 10.1158/1078-0432.CCR-21-0121 PMC8416924

[299] Zhao D, Zhang M, Huang S, Liu Q, Zhu S, Li Y, et al. CHD6 promotes broad nucleosome eviction for transcriptional activation in prostate cancer cells. Nucleic Acids Res. 2022;50(21):12186–201. 36408932 10.1093/nar/gkac1090 PMC9757051

[300] Bu L, Huang S, Rao Z, Wu C, Sun B-Y, Liu Y, et al. CHD6 eviction of promoter nucleosomes maintains housekeeping transcriptional program in prostate cancer. Molecular Therapy Nucleic Acids. 2024;35(4):102397. 39717618 10.1016/j.omtn.2024.102397 PMC11665337

[301] Damaschke NA, Yang B, Blute ML Jr, Lin CP, Huang W, Jarrard DF. Frequent disruption of chromodomain helicase DNA-binding protein 8 (CHD8) and functionally associated chromatin regulators in prostate cancer. Neoplasia. 2014;16(12):1018–27. 25499215 10.1016/j.neo.2014.10.003 PMC4309256

[302] Menon T, Yates JA, Bochar DA. Regulation of androgen-responsive transcription by the chromatin remodeling factor CHD8. Mol Endocrinol. 2010;24(6):1165–74. 20308527 10.1210/me.2009-0421 PMC2875808

[303] Ishihara K, Oshimura M, Nakao M. CTCF-Dependent Chromatin Insulator Is Linked to Epigenetic Remodeling. Mol Cell. 2006;23(5):733–42. 16949368 10.1016/j.molcel.2006.08.008

[304] Zhu C, Gong Z, Yin P, Huang J, He D, Zhu B, et al. Predictive role of ARID1A and B2M mutations and the antigen presentation pathway in the efficacy of definitive chemoradiotherapy for cervical cancer. The Oncologist. 2025;30(6):oyaf133. 40536270 10.1093/oncolo/oyaf133 PMC12204396

[305] Katagiri A, Nakayama K, Rahman MT, Rahman M, Katagiri H, Ishikawa M, et al. Frequent Loss of Tumor Suppressor ARID1A Protein Expression in Adenocarcinomas/Adenosquamous Carcinomas of the Uterine Cervix. Int J Gynecol Cancer. 2012;22(2):208–12. 22274316 10.1097/IGC.0b013e3182313d78

[306] Cho H, Kim JS-Y, Chung H, Perry C, Lee H, Kim J-H. Loss of ARID1A/BAF250a expression is linked to tumor progression and adverse prognosis in cervical cancer. Hum Pathol. 2013;44(7):1365–74. 23427874 10.1016/j.humpath.2012.11.007

[307] Chang L, Azzolin L, Di Biagio D, Zanconato F, Battilana G, Lucon Xiccato R, et al. The SWI/SNF complex is a mechanoregulated inhibitor of YAP and TAZ. Nature. 2018;563(7730):265–9. 30401838 10.1038/s41586-018-0658-1 PMC7612964

[308] Wang Q, Cao Z, Wei Y, Zhang J, Cheng Z. Potential Role of SWI/SNF Complex Subunit Actin-Like Protein 6A in Cervical Cancer. Front Oncol. 2021;11:724832. 34395295 10.3389/fonc.2021.724832 PMC8358818

[309] Zhao J, Li L, Yang T. MiR-216a-3p suppresses the proliferation and invasion of cervical cancer through downregulation of ACTL6A-mediated YAP signaling. J Cell Physiol. 2020;235(12):9718–28. 32401366 10.1002/jcp.29783

[310] Lee K, Lee A-Y, Kwon YK, Kwon H. Suppression of HPV E6 and E7 expression by BAF53 depletion in cervical cancer cells. Biochem Biophys Res Commun. 2011;412(2):328–33. 21821000 10.1016/j.bbrc.2011.07.098

[311] Fudulu A, Diaconu CC, Iancu IV, Plesa A, Albulescu A, Bostan M, et al. Exploring the role of E6 and E7 oncoproteins in cervical oncogenesis through MBD2/3-NuRD complex chromatin remodeling. Genes (Basel). 2024;15(5):560. 38790189 10.3390/genes15050560 PMC11121560

[312] Brehm A, Nielsen SJ, Miska EA, McCance DJ, Reid JL, Bannister AJ, et al. The E7 oncoprotein associates with Mi2 and histone deacetylase activity to promote cell growth. EMBO J. 1999;18(9):2449–58. 10228159 10.1093/emboj/18.9.2449 PMC1171327

[313] Mehta K, Gunasekharan V, Satsuka A, Laimins LA. Human papillomaviruses activate and recruit SMC1 cohesin proteins for the differentiation-dependent life cycle through association with CTCF insulators. PLoS Pathog. 2015;11(4):e1004763. 25875106 10.1371/journal.ppat.1004763 PMC4395367

[314] Pentland I, Campos-León K, Cotic M, Davies K-J, Wood CD, Groves IJ, et al. Disruption of CTCF-YY1–dependent looping of the human papillomavirus genome activates differentiation-induced viral oncogene transcription. PLoS Biol. 2018;16(10):e2005752. 30359362 10.1371/journal.pbio.2005752 PMC6219814

[315] Hu J, Liu J, Chen A, Lyu J, Ai G, Zeng Q, et al. Ino80 promotes cervical cancer tumorigenesis by activating Nanog expression. Oncotarget. 2016;7(44):72250–62. 27750218 10.18632/oncotarget.12667 PMC5342159

[316] Rozenblatt-Rosen O, Deo RC, Padi M, Adelmant G, Calderwood MA, Rolland T, et al. Interpreting cancer genomes using systematic host network perturbations by tumour virus proteins. Nature. 2012;487(7408):491–5. 22810586 10.1038/nature11288 PMC3408847

[317] Xia L, Wang M, Li H, Tang X, Chen F, Cui J. The effect of aberrant expression and genetic polymorphisms of Rad21 on cervical cancer biology. Cancer Med. 2018;7(7):3393–405. 29797792 10.1002/cam4.1592 PMC6051231

[318] Wagle NS, Nogueira L, Devasia TP, Mariotto AB, Yabroff KR, Islami F, et al. Cancer treatment and survivorship statistics. 2025. CA: A Cancer Journal for Clinicians. 2025;75(4):308–40. 10.3322/caac.70011 PMC12223361 40445120

[319] Piergentili R, Marinelli E, De Paola L, Cucinella G, Billone V, Zaami S, et al. Epigenetics of Endometrial Cancer: The Role of Chromatin Modifications and Medicolegal Implications. Int J Mol Sci. 2025;26(15):7306. 40806438 10.3390/ijms26157306 PMC12347409

[320] Köbel M, Hoang LN, Tessier-Cloutier B, Meng B, Soslow RA, Stewart CJR, et al. Undifferentiated Endometrial Carcinomas Show Frequent Loss of Core Switch/Sucrose Nonfermentable Complex Proteins. Am J Surg Pathol. 2018;42(1):76–83. 28863077 10.1097/PAS.0000000000000941 PMC7995487

[321] Stewart CJ, Crook ML. SWI/SNF complex deficiency and mismatch repair protein expression in undifferentiated and dedifferentiated endometrial carcinoma. Pathology. 2015;47(5):439–45. 26126041 10.1097/PAT.0000000000000270

[322] Guan B, Mao T-L, Panuganti PK, Kuhn E, Kurman RJ, Maeda D, et al. Mutation and Loss of Expression of ARID1A in Uterine Low-grade Endometrioid Carcinoma. Am J Surg Pathol. 2011;35(5):625–32. 21412130 10.1097/PAS.0b013e318212782a PMC3077471

[323] St. Laurent JD, Xu GD, Ying AW, Gokbayrak B, Patil A, Paulo JA, et al. Shifted assembly and function of mSWI/SNF family subcomplexes underlie targetable dependencies in dedifferentiated endometrial carcinomas. Nat Genet. 2025;57(11):2743–55. 41116019 10.1038/s41588-025-02333-9

[324] Raffone A, Travaglino A, Saccone G, Cieri M, Mascolo M, Mollo A, et al. Diagnostic and prognostic value of ARID1A in endometrial hyperplasia: a novel marker of occult cancer. APMIS. 2019;127(9):597–606. 31237034 10.1111/apm.12977

[325] Suryo Rahmanto Y, Shen W, Shi X, Chen X, Yu Y, Yu Z-C, et al. Inactivation of Arid1a in the endometrium is associated with endometrioid tumorigenesis through transcriptional reprogramming. Nat Commun. 2020;11(1):2717. 32483112 10.1038/s41467-020-16416-0 PMC7264300

[326] Kihara A, Amano Y, Matsubara D, Fukushima N, Fujiwara H, Niki T. Infrequent loss of SMARCA4, SMARCA2, and SMARCB1 expression in uterine mesenchymal tumors. Hum Pathol. 2021;116:12–21. 34271067 10.1016/j.humpath.2021.07.001

[327] Karnezis AN, Wang Y, Ramos P, Hendricks WP, Oliva E, D’Angelo E, et al. Dual loss of the SWI/SNF complex ATPases SMARCA4/BRG1 and SMARCA2/BRM is highly sensitive and specific for small cell carcinoma of the ovary, hypercalcaemic type. J Pathol. 2016;238(3):389–400. 26356327 10.1002/path.4633 PMC4832362

[328] Karnezis AN, Hoang LN, Coatham M, Ravn S, Almadani N, Tessier-Cloutier B, et al. Loss of switch/sucrose non-fermenting complex protein expression is associated with dedifferentiation in endometrial carcinomas. Mod Pathol. 2016;29(3):302–14. 26743474 10.1038/modpathol.2015.155 PMC4980656

[329] Kandoth C, Schultz N, Cherniack AD, Akbani R, Liu Y, Shen H, et al. Integrated genomic characterization of endometrial carcinoma. Nature. 2013;497(7447):67–73. 23636398 10.1038/nature12113 PMC3704730

[330] Ye L, Jiang G, Sun Y, Li B. ARNTL-mediated INO80-DHX15 axis reprograms the glycolytic metabolism and augments the progression of endometrial carcinoma. Cell Death Dis. 2025;16(1):463. 40541951 10.1038/s41419-025-07776-w PMC12181345

[331] Le Gallo M, O’Hara AJ, Rudd ML, Urick ME, Hansen NF, O’Neil NJ, et al. Exome sequencing of serous endometrial tumors identifies recurrent somatic mutations in chromatin-remodeling and ubiquitin ligase complex genes. Nat Genet. 2012;44(12):1310–5. 23104009 10.1038/ng.2455 PMC3515204

[332] Li Y, Liu Q, McGrail DJ, Dai H, Li K, Lin SY. CHD4 mutations promote endometrial cancer stemness by activating TGF-beta signaling. Am J Cancer Res. 2018;8(5):903–14. 29888111 PMC5992509

[333] Zhang Q, Zhu F, Tong Y, Shi D, Zhang J. CHD4 R975H mutant activates tumorigenic pathways and promotes stemness and M2-like macrophage polarization in endometrial cancer. Sci Rep. 2024;14(1):18617. 39127769 10.1038/s41598-024-69233-6 PMC11316823

[334] Bachmann IM, Halvorsen OJ, Collett K, Stefansson IM, Straume O, Haukaas SA, et al. EZH2 Expression Is Associated With High Proliferation Rate and Aggressive Tumor Subgroups in Cutaneous Melanoma and Cancers of the Endometrium, Prostate, and Breast. J Clin Oncol. 2006;24(2):268–73. 16330673 10.1200/JCO.2005.01.5180

[335] Krill L, Deng W, Eskander R, Mutch D, Zweizig S, Hoang B, et al. Overexpression of enhance of Zeste homolog 2 (EZH2) in endometrial carcinoma: An NRG Oncology/Gynecologic Oncology Group Study. Gynecol Oncol. 2020;156(2):423–9. 31843273 10.1016/j.ygyno.2019.12.003 PMC7103063

[336] Ihira K, Dong P, Xiong Y, Watari H, Konno Y, Hanley SJ, et al. EZH2 inhibition suppresses endometrial cancer progression via miR-361/Twist axis. Oncotarget. 2017;8(8):13509. 28088786 10.18632/oncotarget.14586 PMC5355116

[337] Sahoo SS, Ramanand SG, Cuevas IC, Gao Y, Lee S, Abbas A, et al. A distinct mechanism of epigenetic reprogramming silences PAX2 and initiates endometrial carcinogenesis. The Journal of clinical investigation. 2025;135(16):e190989. 40829174 10.1172/JCI190989 PMC12352900

[338] Nagarajan S, Rao SV, Sutton J, Cheeseman D, Dunn S, Papachristou EK, et al. ARID1A influences HDAC1/BRD4 activity, intrinsic proliferative capacity and breast cancer treatment response. Nat Genet. 2020;52(2):187–97. 31913353 10.1038/s41588-019-0541-5 PMC7116647

[339] Xu G, Chhangawala S, Cocco E, Razavi P, Cai Y, Otto JE, et al. ARID1A determines luminal identity and therapeutic response in estrogen-receptor-positive breast cancer. Nat Genet. 2020;52(2):198–207. 31932695 10.1038/s41588-019-0554-0 PMC7341683

[340] Sheng WY, Zhu Y, Liu SQ, Huang QY, Qian WF, Cheng JL, et al. A novel SWI/SNF complex promotes triple-negative breast cancer progression. Cell Mol Biol Lett. 2025;30(1):105. 40890601 10.1186/s11658-025-00788-6 PMC12403323

[341] Sun Z, Li Z, Wei Y, Xu L, Hang X, Kang Y. SMARCA4 Inhibits Breast Cancer Progression and Metastasis through RHOA Suppression. Cancer Res. 2025;85(10):1803–18. 39992701 10.1158/0008-5472.CAN-24-2801 PMC12081196

[342] Bochar DA, Wang L, Beniya H, Kinev A, Xue Y, Lane WS, et al. BRCA1 is associated with a human SWI/SNF-related complex: linking chromatin remodeling to breast cancer. Cell. 2000;102(2):257–65. 10943845 10.1016/s0092-8674(00)00030-1

[343] Harte MT, O’Brien GJ, Ryan NM, Gorski JJ, Savage KI, Crawford NT, et al. BRD7, a subunit of SWI/SNF complexes, binds directly to BRCA1 and regulates BRCA1-dependent transcription. Cancer Res. 2010;70(6):2538–47. 20215511 10.1158/0008-5472.CAN-09-2089

[344] Mondal J, Zhang J, Qing F, Li S, Kumar D, Huse JT, et al. Brd7 loss reawakens dormant metastasis initiating cells in lung by forging an immunosuppressive niche. Nat Commun. 2025;16(1):1378. 39910049 10.1038/s41467-025-56347-2 PMC11799300

[345] Sun Y, Xu X, Zhao W, Zhang Y, Chen K, Li Y, et al. RAD21 is the core subunit of the cohesin complex involved in directing genome organization. Genome Biol. 2023;24(1):155. 37381036 10.1186/s13059-023-02982-1 PMC10303866

[346] Yan M, Xu H, Waddell N, Shield-Artin K, Haviv I, McKay MJ, et al. Enhanced RAD21 cohesin expression confers poor prognosis in BRCA2 and BRCAX, but not BRCA1 familial breast cancers. Breast Cancer Res. 2012;14(2):R69. 22537934 10.1186/bcr3176 PMC3446404

[347] Xu H, Yan M, Patra J, Natrajan R, Yan Y, Swagemakers S, et al. Enhanced RAD21 cohesin expression confers poor prognosis and resistance to chemotherapy in high grade luminal, basal and HER2 breast cancers. Breast Cancer Res. 2011;13(1):R9. 21255398 10.1186/bcr2814 PMC3109576

[348] Yun J, Song SH, Kim HP, Han SW, Yi EC, Kim TY. Dynamic cohesin-mediated chromatin architecture controls epithelial-mesenchymal plasticity in cancer. EMBO Rep. 2016;17(9):1343–59. 27466323 10.15252/embr.201541852 PMC5007572

[349] Larcher A, Campi R, Bex A, Bray F, Bukavina L, Jonasch E, et al. Epidemiology of Renal Cancer: Incidence, Mortality, Survival, Genetic Predisposition, and Risk Factors. Eur Urol. 2025;88(4):341–58. 40750496 10.1016/j.eururo.2025.06.005

[350] Pal SK, Ali SM, Yakirevich E, Geynisman DM, Karam JA, Elvin JA, et al. Characterization of Clinical Cases of Advanced Papillary Renal Cell Carcinoma via Comprehensive Genomic Profiling. Eur Urol. 2018;73(1):71–8. 28592388 10.1016/j.eururo.2017.05.033

[351] Varela I, Tarpey P, Raine K, Huang D, Ong CK, Stephens P, et al. Exome sequencing identifies frequent mutation of the SWI/SNF complex gene PBRM1 in renal carcinoma. Nature. 2011;469(7331):539–42. 21248752 10.1038/nature09639 PMC3030920

[352] Miao D, Margolis CA, Gao W, Voss MH, Li W, Martini DJ, et al. Genomic correlates of response to immune checkpoint therapies in clear cell renal cell carcinoma. Science. 2018;359(6377):801–6. 29301960 10.1126/science.aan5951 PMC6035749

[353] Hakimi AA, Attalla K, DiNatale RG, Ostrovnaya I, Flynn J, Blum KA, et al. A pan-cancer analysis of PBAF complex mutations and their association with immunotherapy response. Nat Commun. 2020;11(1):4168. 32820162 10.1038/s41467-020-17965-0 PMC7441387

[354] Yao X, Hong JH, Nargund AM, Ng MSW, Heng HL, Li Z, et al. PBRM1-deficient PBAF complexes target aberrant genomic loci to activate the NF-κB pathway in clear cell renal cell carcinoma. Nat Cell Biol. 2023;25(5):765–77. 37095322 10.1038/s41556-023-01122-y

[355] Calderaro J, Masliah-Planchon J, Richer W, Maillot L, Maille P, Mansuy L, et al. Balanced Translocations Disrupting SMARCB1 Are Hallmark Recurrent Genetic Alterations in Renal Medullary Carcinomas. Eur Urol. 2016;69(6):1055–61. 26433572 10.1016/j.eururo.2015.09.027

[356] Rizzo M, Caliò A, Brunelli M, Pezzicoli G, Ganini C, Martignoni G, et al. Clinico-pathological implications of the 2022 WHO renal cell carcinoma classification. Cancer Treat Rev. 2022;116:102558. 10.1016/j.ctrv.2023.102558 37060647

[357] Jia L, Carlo MI, Khan H, Nanjangud GJ, Rana S, Cimera R, et al. Distinctive mechanisms underlie the loss of SMARCB1 protein expression in renal medullary carcinoma: morphologic and molecular analysis of 20 cases. Mod Pathol. 2019;32(9):1329–43. 30980040 10.1038/s41379-019-0273-1 PMC6731129

[358] Vokshi BH, Davidson G, Tawanaie Pour Sedehi N, Helleux A, Rippinger M, Haller AR, et al. SMARCB1 regulates a TFCP2L1-MYC transcriptional switch promoting renal medullary carcinoma transformation and ferroptosis resistance. Nat Commun. 2023;14(1):3034. 10.1038/s41467-023-38472-y PMC10220073 37236926

[359] Msaouel P, Malouf GG, Su X, Yao H, Tripathi DN, Soeung M, et al. Comprehensive Molecular Characterization Identifies Distinct Genomic and Immune Hallmarks of Renal Medullary Carcinoma. Cancer Cell. 2020;37(5):720-34.e13. 32359397 10.1016/j.ccell.2020.04.002 PMC7288373

[360] Colli LM, Jessop L, Myers TA, Camp SY, Machiela MJ, Choi J, et al. Altered regulation of DPF3, a member of the SWI/SNF complexes, underlies the 14q24 renal cancer susceptibility locus. Am J Hum Genet. 2021;108(9):1590–610. 34390653 10.1016/j.ajhg.2021.07.009 PMC8456159

[361] Cui H, Yi H, Bao H, Tan Y, Tian C, Shi X, et al. The SWI/SNF chromatin remodeling factor DPF3 regulates metastasis of ccRCC by modulating TGF-β signaling. Nat Commun. 2022;13(1):4680. 35945219 10.1038/s41467-022-32472-0 PMC9363427

[362] Alessi JV, Ricciuti B, Spurr LF, Gupta H, Li YY, Glass C, et al. SMARCA4 and Other SWItch/Sucrose NonFermentable Family Genomic Alterations in NSCLC: Clinicopathologic Characteristics and Outcomes to Immune Checkpoint Inhibition. J Thorac Oncol. 2021;16(7):1176–87. 33845210 10.1016/j.jtho.2021.03.024

[363] Redin E, Sridhar H, Zhan YA, Pereira Mello B, Zhong H, Durani V, et al. SMARCA4 controls state plasticity in small cell lung cancer through regulation of neuroendocrine transcription factors and REST splicing. J Hematol Oncol. 2024;17(1):58. 39080761 10.1186/s13045-024-01572-3 PMC11290012

[364] Reisman DN, Sciarrotta J, Wang W, Funkhouser WK, Weissman BE. Loss of BRG1/BRM in human lung cancer cell lines and primary lung cancers: correlation with poor prognosis. Cancer Res. 2003;63(3):560–6. 12566296

[365] Liu G, Gramling S, Munoz D, Cheng D, Azad AK, Mirshams M, et al. Two novel BRM insertion promoter sequence variants are associated with loss of BRM expression and lung cancer risk. Oncogene. 2011;30(29):3295–304. 21478907 10.1038/onc.2011.81 PMC3400140

[366] Liu G, Cuffe S, Liang S, Azad AK, Cheng L, Brhane Y, et al. BRM promoter polymorphisms and survival of advanced non-small cell lung cancer patients in the Princess Margaret cohort and CCTG BR.24 trial. Clin Cancer Res. 2017;23(10):2460–70. 27827316 10.1158/1078-0432.CCR-16-1640 PMC5422138

[367] Concepcion CP, Ma S, LaFave LM, Bhutkar A, Liu M, DeAngelo LP, et al. Smarca4 Inactivation Promotes Lineage-Specific Transformation and Early Metastatic Features in the Lung. Cancer Discov. 2022;12(2):562–85. 34561242 10.1158/2159-8290.CD-21-0248 PMC8831463

[368] Romero OA, Setien F, John S, Gimenez-Xavier P, Gómez-López G, Pisano D, et al. The tumour suppressor and chromatin-remodelling factor BRG1 antagonizes Myc activity and promotes cell differentiation in human cancer. EMBO Mol Med. 2012;4(7):603–16. 22407764 10.1002/emmm.201200236 PMC3407948

[369] Romero OA, Torres-Diz M, Pros E, Savola S, Gomez A, Moran S, et al. MAX inactivation in small cell lung cancer disrupts MYC-SWI/SNF programs and is synthetic lethal with BRG1. Cancer Discov. 2014;4(3):292–303. 24362264 10.1158/2159-8290.CD-13-0799

[370] Xue Y, Meehan B, Fu Z, Wang XQD, Fiset PO, Rieker R, et al. SMARCA4 loss is synthetic lethal with CDK4/6 inhibition in non-small cell lung cancer. Nat Commun. 2019;10(1):557. 30718506 10.1038/s41467-019-08380-1 PMC6362083

[371] Gupta M, Concepcion CP, Fahey CG, Keshishian H, Bhutkar A, Brainson CF, et al. BRG1 Loss Predisposes Lung Cancers to Replicative Stress and ATR Dependency. Cancer Res. 2020;80(18):3841–54. 32690724 10.1158/0008-5472.CAN-20-1744 PMC7501156

[372] Watanabe H, Mizutani T, Haraguchi T, Yamamichi N, Minoguchi S, Yamamichi-Nishina M, et al. SWI/SNF complex is essential for NRSF-mediated suppression of neuronal genes in human nonsmall cell lung carcinoma cell lines. Oncogene. 2006;25(3):470–9. 16247481 10.1038/sj.onc.1209068

[373] Glaros S, Cirrincione GM, Muchardt C, Kleer CG, Michael CW, Reisman D. The reversible epigenetic silencing of BRM: implications for clinical targeted therapy. Oncogene. 2007;26(49):7058–66. 17546055 10.1038/sj.onc.1210514

[374] Vangamudi B, Paul TA, Shah PK, Kost-Alimova M, Nottebaum L, Shi X, et al. The SMARCA2/4 ATPase Domain Surpasses the Bromodomain as a Drug Target in SWI/SNF-Mutant Cancers: Insights from cDNA Rescue and PFI-3 Inhibitor Studies. Cancer Res. 2015;75(18):3865–78. 26139243 10.1158/0008-5472.CAN-14-3798 PMC4755107

[375] Hoffman GR, Rahal R, Buxton F, Xiang K, McAllister G, Frias E, et al. Functional epigenetics approach identifies BRM/SMARCA2 as a critical synthetic lethal target in BRG1-deficient cancers. Proc Natl Acad Sci U S A. 2014;111(8):3128–33. 24520176 10.1073/pnas.1316793111 PMC3939885

[376] Field NR, Dickson KA, Nassif NT, Marsh DJ. SMARCA4 and SMARCA2 co-deficiency: An uncommon molecular signature defining a subset of rare, aggressive and undifferentiated malignancies associated with defective chromatin remodeling. Cancer Lett. 2024;605:217282. 39369768 10.1016/j.canlet.2024.217282

[377] Xue Y, Morris JL, Yang K, Fu Z, Zhu X, Johnson F, et al. SMARCA4/2 loss inhibits chemotherapy-induced apoptosis by restricting IP3R3-mediated Ca(2+) flux to mitochondria. Nat Commun. 2021;12(1):5404. 34518526 10.1038/s41467-021-25260-9 PMC8438089

[378] Wang Y, Meraz IM, Qudratullah M, Kotagiri S, Han Y, Xi Y, et al. Mutation of SMARCA4 Induces Cancer Cell-Intrinsic Defects in the Enhancer Landscape and Resistance to Immunotherapy. Cancer Res. 2025;85(11):1997–2013. 40080526 10.1158/0008-5472.CAN-24-2054 PMC12127800

[379] de Miguel FJ, Gentile C, Feng WW, Silva SJ, Sankar A, Exposito F, et al. Mammalian SWI/SNF chromatin remodeling complexes promote tyrosine kinase inhibitor resistance in EGFR-mutant lung cancer. Cancer Cell. 2023;41(8):1516-34.e9. 37541244 10.1016/j.ccell.2023.07.005 PMC10957226

[380] Papadakis AI, Sun C, Knijnenburg TA, Xue Y, Grernrum W, Hölzel M, et al. SMARCE1 suppresses EGFR expression and controls responses to MET and ALK inhibitors in lung cancer. Cell Res. 2015;25(4):445–58. 25656847 10.1038/cr.2015.16 PMC4387553

[381] Liu X, Li Z, Wang Z, Liu F, Zhang L, Ke J, et al. Chromatin Remodeling Induced by ARID1A Loss in Lung Cancer Promotes Glycolysis and Confers JQ1 Vulnerability. Cancer Res. 2022;82(5):791–804. 34987057 10.1158/0008-5472.CAN-21-0763

[382] Xiao C, Fan T, Wang D, Yin H, Deng Z, Cai W, et al. RAD21-mediated epigenetic regulation promotes lung adenocarcinoma progression and sensitizes cancer cells to ERK-targeted therapy. Cancer Lett. 2025;634:218062. 41005454 10.1016/j.canlet.2025.218062

[383] Alaggio R, Amador C, Anagnostopoulos I, Attygalle AD, Araujo IBdO, Berti E, et al. The 5th edition of the World Health Organization Classification of Haematolymphoid Tumours: Lymphoid Neoplasms. Leukemia. 2022;36(7):1720–48. 10.1038/s41375-022-01620-2 PMC9214472 35732829

[384] Lunning MA, Green MR. Mutation of chromatin modifiers; an emerging hallmark of germinal center B-cell lymphomas. Blood Cancer Journal. 2015;5(10):e361-e. 10.1038/bcj.2015.89 PMC4635197 26473533

[385] Wienand K, Chapuy B, Stewart C, Dunford AJ, Wu D, Kim J, et al. Genomic analyses of flow-sorted Hodgkin Reed-Sternberg cells reveal complementary mechanisms of immune evasion. Blood Adv. 2019;3(23):4065–80. 31816062 10.1182/bloodadvances.2019001012 PMC6963251

[386] Antoniolli M, Solovey M, Hildebrand JA, Freyholdt T, Strobl CD, Bararia D, et al. ARID1A mutations protect follicular lymphoma from FAS-dependent immune surveillance by reducing RUNX3/ETS1-driven FAS-expression. Cell Death Differ. 2025;32(5):899–910. 39843653 10.1038/s41418-025-01445-3 PMC12089402

[387] Ma MCJ, Tadros S, Bouska A, Heavican T, Yang H, Deng Q, et al. Subtype-specific and co-occurring genetic alterations in B-cell non-Hodgkin lymphoma. Haematologica. 2022;107(3):690–701. 33792219 10.3324/haematol.2020.274258 PMC8883549

[388] Yusufova N, Kloetgen A, Teater M, Osunsade A, Camarillo JM, Chin CR, et al. Histone H1 loss drives lymphoma by disrupting 3D chromatin architecture. Nature. 2021;589(7841):299–305. 33299181 10.1038/s41586-020-3017-y PMC7855728

[389] Deng Q, Lakra P, Gou P, Yang H, Meydan C, Teater M, et al. SMARCA4 is a haploinsufficient B cell lymphoma tumor suppressor that fine-tunes centrocyte cell fate decisions. Cancer Cell. 2024;42(4):605-22.e11. 38458188 10.1016/j.ccell.2024.02.011 PMC11003852

[390] Yi S, Yan Y, Jin M, Bhattacharya S, Wang Y, Wu Y, et al. Genomic and transcriptomic profiling reveals distinct molecular subsets associated with outcomes in mantle cell lymphoma. The Journal of Clinical Investigation. 2021;132(3):e153283. 34882582 10.1172/JCI153283 PMC8803323

[391] Andrades A, Peinado P, Alvarez-Perez JC, Sanjuan-Hidalgo J, García DJ, Arenas AM, et al. SWI/SNF complexes in hematological malignancies: biological implications and therapeutic opportunities. Mol Cancer. 2023;22(1):39. 36810086 10.1186/s12943-023-01736-8 PMC9942420

[392] Yan Y, Shan D, Yu Y, Chen W, Zhang X, Wenjie X, et al. TENT5C as a target of chromatin remodeler SMARCA2 participates BTK inhibitor resistance in mantle cell lymphoma by regulating ATP metabolism. Blood. 2024;144(Supplement 1):832.

[393] Mroczek S, Chlebowska J, Kuliński TM, Gewartowska O, Gruchota J, Cysewski D, et al. The non-canonical poly(A) polymerase FAM46C acts as an onco-suppressor in multiple myeloma. Nat Commun. 2017;8(1):619. 28931820 10.1038/s41467-017-00578-5 PMC5606997

[394] Rivas MA, Meydan C, Chin CR, Challman MF, Kim D, Bhinder B, et al. Smc3 dosage regulates B cell transit through germinal centers and restricts their malignant transformation. Nat Immunol. 2021;22(2):240–53. 33432228 10.1038/s41590-020-00827-8 PMC7855695

[395] Rivas MA, Durmaz C, Kloetgen A, Chin CR, Chen Z, Bhinder B, et al. Cohesin Core Complex Gene Dosage Contributes to Germinal Center Derived Lymphoma Phenotypes and Outcomes. Frontiers in Immunology. 2021; 12:688493. 34621263 10.3389/fimmu.2021.688493 PMC8490713

[396] Lu Y, Jiang J, He Z, Bao Z, Chen X, Cheng J. Molecular characteristics and oncogenic role of CHD family genes: a pan-cancer analysis based on bioinformatic and biological analysis. Sci Rep. 2024;14(1):18923. 39143142 10.1038/s41598-024-68644-9 PMC11324730

[397] Yoshida T, Hu Y, Zhang Z, Emmanuel AO, Galani K, Muhire B, et al. Chromatin restriction by the nucleosome remodeler Mi-2β and functional interplay with lineage-specific transcription regulators control B-cell differentiation. Genes Dev. 2019;33(13–14):763–81. 31123064 10.1101/gad.321901.118 PMC6601517

[398] Loughran SJ, Comoglio F, Hamey FK, Giustacchini A, Errami Y, Earp E, et al. Mbd3/NuRD controls lymphoid cell fate and inhibits tumorigenesis by repressing a B cell transcriptional program. J Exp Med. 2017;214(10):3085–104. 28899870 10.1084/jem.20161827 PMC5626393

[399] Gambi G, Boccalatte F, Rodriguez Hernaez J, Lin Z, Nadorp B, Polyzos A, et al. 3D chromatin hubs as regulatory units of identity and survival in human acute leukemia. Mol Cell. 2025;85(1):42-60.e7. 39719705 10.1016/j.molcel.2024.11.040 PMC11934262

[400] Kloetgen A, Thandapani P, Ntziachristos P, Ghebrechristos Y, Nomikou S, Lazaris C, et al. Three-dimensional chromatin landscapes in T cell acute lymphoblastic leukemia. Nat Genet. 2020;52(4):388–400. 32203470 10.1038/s41588-020-0602-9 PMC7138649

[401] Peng L, Li J, Wu J, Xu B, Wang Z, Giamas G, et al. A Pan-Cancer Analysis of SMARCA4 Alterations in Human Cancers. Frontiers in Immunology. 2021;12:762598. 34675941 10.3389/fimmu.2021.762598 PMC8524462

[402] Kang Q, Ma D, Zhao P, Chai X, Huang Y, Gao R, et al. BRG1 promotes progression of B-cell acute lymphoblastic leukemia by disrupting PPP2R1A transcription. Cell Death Dis. 2024;15(8):621. 39187513 10.1038/s41419-024-06996-w PMC11347705

[403] Buscarlet M, Krasteva V, Ho L, Simon C, Hébert J, Wilhelm B, et al. Essential role of BRG, the ATPase subunit of BAF chromatin remodeling complexes, in leukemia maintenance. Blood. 2014;123(11):1720–8. 24478402 10.1182/blood-2013-02-483495 PMC3954053

[404] Lee SI, Celik S, Logsdon BA, Lundberg SM, Martins TJ, Oehler VG, et al. A machine learning approach to integrate big data for precision medicine in acute myeloid leukemia. Nat Commun. 2018;9(1):42. 29298978 10.1038/s41467-017-02465-5 PMC5752671

[405] Fiskus W, Mill CP, Piel J, Collins M, Hentemann M, Cuglievan B, et al. Superior preclinical efficacy of co-treatment with BRG1/BRM and FLT3 inhibitor against AML cells with FLT3 mutations. Blood Cancer J. 2025;15(1):40. 40089460 10.1038/s41408-025-01251-7 PMC11910597

[406] Stratmann S, Yones SA, Sun J, Skaftason A, Mayrhofer M, Norgren N, et al. Genomic and Transcriptomic Characterization of Adult and Pediatric Relapsed Acute Myeloid Leukemia Reveals Novel Therapeutic Targets. Blood. 2020;136:37–8.

[407] Zhang J, Bajari R, Andric D, Gerthoffert F, Lepsa A, Nahal-Bose H, et al. The International Cancer Genome Consortium Data Portal. Nat Biotechnol. 2019;37(4):367–9. 30877282 10.1038/s41587-019-0055-9

[408] Stratmann S, Yones SA, Mayrhofer M, Norgren N, Skaftason A, Sun J, et al. Genomic characterization of relapsed acute myeloid leukemia reveals novel putative therapeutic targets. Blood Adv. 2021;5(3):900–12. 33560403 10.1182/bloodadvances.2020003709 PMC7876890

[409] Ren T, Wang J, Tang W, Chen D, Wang S, Zhang X, et al. ARID1A has prognostic value in acute myeloid leukemia and promotes cell proliferation via TGF-β1/SMAD3 signaling. Clin Exp Med. 2023;23(3):777–85. 35867200 10.1007/s10238-022-00863-8

[410] Han L, Madan V, Mayakonda A, Dakle P, Woon TW, Shyamsunder P, et al. Chromatin remodeling mediated by ARID1A is indispensable for normal hematopoiesis in mice. Leukemia. 2019;33(9):2291–305. 30858552 10.1038/s41375-019-0438-4 PMC6756219

[411] Yaser H, Gözde T, Aditya H, Shabnam K, Johan B, Esmat Kamali D, et al. The chromatin-remodeling factor CHD4 is required for maintenance of childhood acute myeloid leukemia. Haematologica. 2018;103(7):1169–81. 29599201 10.3324/haematol.2017.183970 PMC6029541

[412] Sperlazza J, Rahmani M, Beckta J, Aust M, Hawkins E, Wang SZ, et al. Depletion of the chromatin remodeler CHD4 sensitizes AML blasts to genotoxic agents and reduces tumor formation. Blood. 2015;126(12):1462–72. 26265695 10.1182/blood-2015-03-631606 PMC4573869

[413] Hsu J, Huang HT, Lee CT, Choudhuri A, Wilson NK, Abraham BJ, et al. CHD7 and Runx1 interaction provides a braking mechanism for hematopoietic differentiation. Proc Natl Acad Sci U S A. 2020;117(38):23626–35. 32883883 10.1073/pnas.2003228117 PMC7519295

[414] Zhen T, Kwon EM, Zhao L, Hsu J, Hyde RK, Lu Y, et al. Chd7 deficiency delays leukemogenesis in mice induced by Cbfb-MYH11. Blood. 2017;130(22):2431–42. 29018080 10.1182/blood-2017-04-780106 PMC5709785

[415] Yang D, Zhang T, Yang Y, Zhang D, Zhou Z, Wang N, et al. Integration of CRISPR-Cas9 screens and multi-omics profiling reveals chromodomain helicase DNA binding protein 7-angiopoietin-1 as a novel multidrug resistance axis in acute myeloid leukemia. Interdisciplinary Medicine. 2025;3(5):e70053.

[416] Chang TC, Chen W, Qu C, Cheng Z, Hedges D, Elsayed A, et al. Genomic Determinants of Outcome in Acute Lymphoblastic Leukemia. J Clin Oncol. 2024;42(29):3491–503. 39121442 10.1200/JCO.23.02238 PMC11458106

[417] Xu X, Chan AKN, Li M, Liu Q, Mattson N, Pangeni Pokharel S, et al. ACTR5 controls CDKN2A and tumor progression in an INO80-independent manner. Sci Adv. 2022;8(51):eadc8911. 36563143 10.1126/sciadv.adc8911 PMC9788768

[418] Eckardt J-N, Stasik S, Röllig C, Sauer T, Scholl S, Hochhaus A, et al. Alterations of cohesin complex genes in acute myeloid leukemia: differential co-mutations, clinical presentation and impact on outcome. Blood Cancer J. 2023;13(1):18. 36693840 10.1038/s41408-023-00790-1 PMC9873811

[419] Kon A, Shih L-Y, Minamino M, Sanada M, Shiraishi Y, Nagata Y, et al. Recurrent mutations in multiple components of the cohesin complex in myeloid neoplasms. Nat Genet. 2013;45(10):1232–7. 23955599 10.1038/ng.2731

[420] Thota S, Viny AD, Makishima H, Spitzer B, Radivoyevitch T, Przychodzen B, et al. Genetic alterations of the cohesin complex genes in myeloid malignancies. Blood. 2014;124(11):1790–8. 25006131 10.1182/blood-2014-04-567057 PMC4162108

[421] Fischer A, Hernández-Rodríguez B, Mulet-Lazaro R, Nuetzel M, Hölzl F, van Herk S, et al. STAG2 mutations reshape the cohesin-structured spatial chromatin architecture to drive gene regulation in acute myeloid leukemia. Cell Rep. 2024;43(8):114498. 39084219 10.1016/j.celrep.2024.114498

[422] Uddin MS, Mamun AA, Alghamdi BS, Tewari D, Jeandet P, Sarwar MS, et al. Epigenetics of glioblastoma multiforme: From molecular mechanisms to therapeutic approaches. Semin Cancer Biol. 2022;83:100–20. 33370605 10.1016/j.semcancer.2020.12.015

[423] Liau BB, Sievers C, Donohue LK, Gillespie SM, Flavahan WA, Miller TE, et al. Adaptive Chromatin Remodeling Drives Glioblastoma Stem Cell Plasticity and Drug Tolerance. Cell Stem Cell. 2017;20(2):233-46.e7. 27989769 10.1016/j.stem.2016.11.003 PMC5291795

[424] Mota M, Sweha SR, Pun M, Natarajan SK, Ding Y, Chung C, et al. Targeting SWI/SNF ATPases in H3.3K27M diffuse intrinsic pontine gliomas. Proc Natl Acad Sci U S A. 2023;120(18):e2221175120. 37094128 10.1073/pnas.2221175120 PMC10161095

[425] Panditharatna E, Marques JG, Wang T, Trissal MC, Liu I, Jiang L, et al. BAF Complex Maintains Glioma Stem Cells in Pediatric H3K27M Glioma. Cancer Discov. 2022;12(12):2880–905. 36305736 10.1158/2159-8290.CD-21-1491 PMC9716260

[426] Mo Y, Duan S, Zhang X, Hua X, Zhou H, Wei HJ, et al. Epigenome Programming by H3.3K27M Mutation Creates a Dependence of Pediatric Glioma on SMARCA4. Cancer Discov. 2022;12(12):2906–29. 10.1158/2159-8290.CD-21-1492 PMC9722525 36305747

[427] Erkek S, Johann PD, Finetti MA, Drosos Y, Chou HC, Zapatka M, et al. Comprehensive Analysis of Chromatin States in Atypical Teratoid/Rhabdoid Tumor Identifies Diverging Roles for SWI/SNF and Polycomb in Gene Regulation. Cancer Cell. 2019;35(1):95-110.e8. 30595504 10.1016/j.ccell.2018.11.014 PMC6341227

[428] Li C, Wang T, Gu J, Qi S, Li J, Chen L, et al. SMARCC2 mediates the regulation of DKK1 by the transcription factor EGR1 through chromatin remodeling to reduce the proliferative capacity of glioblastoma. Cell Death Dis. 2022;13(11):990. 36418306 10.1038/s41419-022-05439-8 PMC9684443

[429] Strub T, Ballotti R, Bertolotto C. The, “ART” of Epigenetics in Melanoma: From histone “Alterations, to Resistance and Therapies.” Theranostics. 2020;10(4):1777–97. 32042336 10.7150/thno.36218 PMC6993228

[430] Guo D, Lui GYL, Lai SL, Wilmott JS, Tikoo S, Jackett LA, et al. RAB27A promotes melanoma cell invasion and metastasis via regulation of pro-invasive exosomes. Int J Cancer. 2019;144(12):3070–85. 30556600 10.1002/ijc.32064

[431] Guo D, Jain R, Hwang JS, Weninger W, Beaumont KA, Tikoo S. RAB27A/Melanophilin Blocker Inhibits Melanoma Cell Motility and Invasion. J Investig Dermatol. 2020;140(7):1470-3.e3. 31978414 10.1016/j.jid.2019.12.023

[432] Guo D, Jurek R, Beaumont KA, Sharp DS, Tan SY, Mariana A, et al. Invasion-Block and S-MARVEL: A high-content screening and image analysis platform identifies ATM kinase as a modulator of melanoma invasion and metastasis. Proc Natl Acad Sci U S A. 2023;120(47):e2303978120. 37963252 10.1073/pnas.2303978120 PMC10666109

[433] Moran B, Silva R, Perry AS, Gallagher WM. Epigenetics of malignant melanoma. Semin Cancer Biol. 2018;51:80–8. 29074395 10.1016/j.semcancer.2017.10.006

[434] Becker TM, Haferkamp S, Dijkstra MK, Scurr LL, Frausto M, Diefenbach E, et al. The chromatin remodelling factor BRG1 is a novel binding partner of the tumor suppressor p16INK4a. Mol Cancer. 2009;8:4. 19149898 10.1186/1476-4598-8-4 PMC2644676

[435] Bock VL, Lyons JG, Huang XX, Jones AM, McDonald LA, Scolyer RA, et al. BRM and BRG1 subunits of the SWI/SNF chromatin remodelling complex are downregulated upon progression of benign skin lesions into invasive tumours. Br J Dermatol. 2011;164(6):1221–7. 21564052 10.1111/j.1365-2133.2011.10267.x

[436] Lin H, Wong RP, Martinka M, Li G. BRG1 expression is increased in human cutaneous melanoma. Br J Dermatol. 2010;163(3):502–10. 20491765 10.1111/j.1365-2133.2010.09851.x

[437] Saladi SV, Keenen B, Marathe HG, Qi H, Chin KV, de la Serna IL. Modulation of extracellular matrix/adhesion molecule expression by BRG1 is associated with increased melanoma invasiveness. Mol Cancer. 2010;9:280. 20969766 10.1186/1476-4598-9-280 PMC3098014

[438] Lin H, Wong RP, Martinka M, Li G. Loss of SNF5 expression correlates with poor patient survival in melanoma. Clin Cancer Res. 2009;15(20):6404–11. 19808872 10.1158/1078-0432.CCR-09-1135

[439] Soshnikova NV, Tatarskiy EV, Tatarskiy VV, Klimenko NS, Shtil AA, Nikiforov MA, et al. PHF10 subunit of PBAF complex mediates transcriptional activation by MYC. Oncogene. 2021;40(42):6071–80. 34465901 10.1038/s41388-021-01994-0 PMC8863208

[440] Grossi E, Nguyen CB, Carcamo S, Kirigin Callaú V, Moran S, Filipescu D, et al. The SWI/SNF PBAF complex facilitates REST occupancy at repressive chromatin. Mol Cell. 2025;85(9):1714-29.e7. 40252649 10.1016/j.molcel.2025.03.026 PMC12048221

[441] Shen CH, Kim SH, Trousil S, Frederick DT, Piris A, Yuan P, et al. Loss of cohesin complex components STAG2 or STAG3 confers resistance to BRAF inhibition in melanoma. Nat Med. 2016;22(9):1056–61. 27500726 10.1038/nm.4155 PMC5014622

[442] Pan D, Kobayashi A, Jiang P, Ferrari de Andrade L, Tay RE, Luoma AM, et al. A major chromatin regulator determines resistance of tumor cells to T cell-mediated killing. Science. 2018;359(6377):770–5. 29301958 10.1126/science.aao1710 PMC5953516

[443] Okada M, Yamasaki S, Nakazato H, Hirahara Y, Ishibashi T, Kawamura M, et al. ARID1A-Deficient Tumors Acquire Immunogenic Neoantigens during the Development of Resistance to Targeted Therapy. Cancer Res. 2024;84(17):2792–805. 39228255 10.1158/0008-5472.CAN-23-2846

[444] Zhang W, Ruan X, Huang Y, Zhang W, Xu G, Zhao J, et al. SETMAR Facilitates the Differentiation of Thyroid Cancer by Regulating SMARCA2-Mediated Chromatin Remodeling. Adv Sci (Weinh). 2024;11(32):e2401712. 38900084 10.1002/advs.202401712 PMC11348079

[445] Landa I, Ibrahimpasic T, Boucai L, Sinha R, Knauf JA, Shah RH, et al. Genomic and transcriptomic hallmarks of poorly differentiated and anaplastic thyroid cancers. J Clin Invest. 2016;126(3):1052–66. 26878173 10.1172/JCI85271 PMC4767360

[446] Ibrahimpasic T, Xu B, Landa I, Dogan S, Middha S, Seshan V, et al. Genomic Alterations in Fatal Forms of Non-Anaplastic Thyroid Cancer: Identification of MED12 and RBM10 as Novel Thyroid Cancer Genes Associated with Tumor Virulence. Clin Cancer Res. 2017;23(19):5970–80. 28634282 10.1158/1078-0432.CCR-17-1183 PMC5626586

[447] Landa I, Pozdeyev N, Korch C, Marlow LA, Smallridge RC, Copland JA, et al. Comprehensive Genetic Characterization of Human Thyroid Cancer Cell Lines: A Validated Panel for Preclinical Studies. Clin Cancer Res. 2019;25(10):3141–51. 30737244 10.1158/1078-0432.CCR-18-2953 PMC6522280

[448] Saqcena M, Leandro-Garcia LJ, Maag JLV, Tchekmedyian V, Krishnamoorthy GP, Tamarapu PP, et al. SWI/SNF Complex Mutations Promote Thyroid Tumor Progression and Insensitivity to Redifferentiation Therapies. Cancer Discov. 2021;11(5):1158–75. 33318036 10.1158/2159-8290.CD-20-0735 PMC8102308

[449] Modena P, Lualdi E, Facchinetti F, Galli L, Teixeira MR, Pilotti S, et al. SMARCB1/INI1 tumor suppressor gene is frequently inactivated in epithelioid sarcomas. Cancer Res. 2005;65(10):4012–9. 15899790 10.1158/0008-5472.CAN-04-3050

[450] Ahn J, Ko M, Lee C, Kim J, Yoon H, Seong RH. Srg3, a mouse homolog of BAF155, is a novel p53 target and acts as a tumor suppressor by modulating p21(WAF1/CIP1) expression. Oncogene. 2011;30(4):445–56. 20935679 10.1038/onc.2010.424

[451] Kato H, Tjernberg A, Zhang W, Krutchinsky AN, An W, Takeuchi T, et al. SYT associates with human SNF/SWI complexes and the C-terminal region of its fusion partner SSX1 targets histones. J Biol Chem. 2002;277(7):5498–505. 11734557 10.1074/jbc.M108702200

[452] de Bruijn DR, Allander SV, van Dijk AH, Willemse MP, Thijssen J, van Groningen JJ, et al. The synovial-sarcoma-associated SS18-SSX2 fusion protein induces epigenetic gene (de)regulation. Cancer Res. 2006;66(19):9474–82. 17018603 10.1158/0008-5472.CAN-05-3726

[453] Svejstrup JQ. Synovial sarcoma mechanisms: a series of unfortunate events. Cell. 2013;153(1):11–2. 23540685 10.1016/j.cell.2013.03.015

[454] McBride MJ, Pulice JL, Beird HC, Ingram DR, D’Avino AR, Shern JF, et al. The SS18-SSX Fusion Oncoprotein Hijacks BAF Complex Targeting and Function to Drive Synovial Sarcoma. Cancer Cell. 2018;33(6):1128-41.e7. 29861296 10.1016/j.ccell.2018.05.002 PMC6791822

[455] Taulli R, Foglizzo V, Morena D, Coda DM, Ala U, Bersani F, et al. Failure to downregulate the BAF53a subunit of the SWI/SNF chromatin remodeling complex contributes to the differentiation block in rhabdomyosarcoma. Oncogene. 2014;33(18):2354–62. 23728344 10.1038/onc.2013.188

[456] Su XA, Ma D, Parsons JV, Replogle JM, Amatruda JF, Whittaker CA, et al. RAD21 is a driver of chromosome 8 gain in Ewing sarcoma to mitigate replication stress. Genes Dev. 2021;35(7–8):556–72. 33766983 10.1101/gad.345454.120 PMC8015718

[457] Surdez D, Zaidi S, Grossetête S, Laud-Duval K, Ferre AS, Mous L, et al. STAG2 mutations alter CTCF-anchored loop extrusion, reduce cis-regulatory interactions and EWSR1-FLI1 activity in Ewing sarcoma. Cancer Cell. 2021;39(6):810-26.e9. 33930311 10.1016/j.ccell.2021.04.001

[458] Liu Y, Xu H, Van der Jeught K, Li Y, Liu S, Zhang L, et al. Somatic mutation of the cohesin complex subunit confers therapeutic vulnerabilities in cancer. J Clin Invest. 2018;128(7):2951–65. 29649003 10.1172/JCI98727 PMC6025969

[459] Li H, Gigi L, Zhao D. CHD1, a multifaceted epigenetic remodeler in prostate cancer. Front Oncol. 2023;13:1123362. 36776288 10.3389/fonc.2023.1123362 PMC9909554

[460] Mandal J, Mandal P, Wang TL, Shih IM. Treating ARID1A mutated cancers by harnessing synthetic lethality and DNA damage response. J Biomed Sci. 2022;29(1):71. 36123603 10.1186/s12929-022-00856-5 PMC9484255

[461] Fukuda K, Nanjo S, Takeuchi S, Chakrabarti T, Brown T, Dev Sahadevan SW, et al. Targeting WEE1 to Overcome ARID1A Mutation-Driven Osimertinib Resistance in EGFR-Mutant Lung Cancer. J Thorac Oncol. 2025;20(10):1459–74. 40518016 10.1016/j.jtho.2025.06.007 PMC13310150

[462] Zhang C, Zhu Y, Ai L, Wang J, Yu S, Wang Y, et al. Targeting WEE1 in ARID1A/TP53 Concurrent Mutant Colorectal Cancer by Exploiting RLoop Accumulation and DNA Repair Deficiencies. Adv Sci (Weinh). 2026;13(9):e12074. 41319275 10.1002/advs.202512074 PMC12904052

[463] Odnokoz O, Banerjee A, Cui X, Zeng L, Uddin A, Li C, et al. Disruption of ARID1B Recruitment to the Nuclear Pore Complex as a New Anticancer Therapeutic Strategy. Adv Sci (Weinh). 2025;12(36):e15585. 40671262 10.1002/advs.202415585 PMC12463049

[464] Lebedev T, Kousar R, Patrick B, Usama M, Lee MK, Tan M, et al. Targeting ARID1A-Deficient Cancers: An Immune-Metabolic Perspective. Cells. 2023;12(6):952. 36980292 10.3390/cells12060952 PMC10047504

[465] Banerjee S, Leary A, Stewart JR, Dewan M, Lheureux S, Clamp AR, et al. 34O ATR inhibitor alone (ceralasertib) or in combination with olaparib in gynaecological cancers with ARID1A loss or no loss: results from the ENGOT/GYN1/NCRI ATARI trial. ESMO Open. 2023;8(1, Supplement 1):100814.

[466] Zhang X, Wang Z, He Y, Wang K, Xiang C, Liu Y, et al. ARID1A loss enhances sensitivity to c-MET inhibition by dual targeting of GPX4 and iron homeostasis, inducing ferroptosis. Cell Death Differ. 2025;32(11):2009–21. 40369167 10.1038/s41418-025-01510-x PMC12572266

[467] Xing T, Li L, Chen Y, Ju G, Li G, Zhu X, et al. Targeting the TCA cycle through cuproptosis confers synthetic lethality on ARID1A-deficient hepatocellular carcinoma. Cell Rep Med. 2023;4(11):101264. 37939712 10.1016/j.xcrm.2023.101264 PMC10694624

[468] Wang Z, Zhang X, Luo Y, Song Y, Xiang C, He Y, et al. Therapeutic targeting of ARID1A-deficient cancer cells with RITA (reactivating p53 and inducing tumor apoptosis). Cell Death Dis. 2024;15(5):375. 38811536 10.1038/s41419-024-06751-1 PMC11136964

[469] Qiu Q, Ding Y, Han J, Guo X, Zhang J, Chen Y, et al. EZH2 deficiency suppresses colorectal cancer progression by inhibiting the mismatch repair pathway and consequently reducing extrachromosomal circular DNA formation. Cell Death Dis. 2026. 10.1038/s41419-026-08919-3. PMC13443750 42236695

[470] Bitler BG, Aird KM, Garipov A, Li H, Amatangelo M, Kossenkov AV, et al. Synthetic lethality by targeting EZH2 methyltransferase activity in ARID1A-mutated cancers. Nat Med. 2015;21(3):231–8. 25686104 10.1038/nm.3799 PMC4352133

[471] Miller RE, Brough R, Bajrami I, Williamson CT, McDade S, Campbell J, et al. Synthetic Lethal Targeting of ARID1A-Mutant Ovarian Clear Cell Tumors with Dasatinib. Mol Cancer Ther. 2016;15(7):1472–84. 27364904 10.1158/1535-7163.MCT-15-0554

[472] Wu C, Lyu J, Yang EJ, Liu Y, Zhang B, Shim JS. Targeting AURKA-CDC25C axis to induce synthetic lethality in ARID1A-deficient colorectal cancer cells. Nat Commun. 2018;9(1):3212. 30097580 10.1038/s41467-018-05694-4 PMC6086874

[473] Lanzi C, Arrighetti N, Pasquali S, Cassinelli G. Targeting EZH2 in SMARCB1-deficient sarcomas: Advances and opportunities to potentiate the efficacy of EZH2 inhibitors. Biochem Pharmacol. 2023;215:115727. 37541451 10.1016/j.bcp.2023.115727

[474] Zhang Y, Wang H, Zhan Z, Gan L, Bai O. Mechanisms of HDACs in cancer development. Front Immunol. 2025;16:1529239. 40260239 10.3389/fimmu.2025.1529239 PMC12009879

[475] Howard TP, Arnoff TE, Song MR, Giacomelli AO, Wang X, Hong AL, et al. MDM2 and MDM4 Are Therapeutic Vulnerabilities in Malignant Rhabdoid Tumors. Cancer Res. 2019;79(9):2404–14. 30755442 10.1158/0008-5472.CAN-18-3066 PMC6497578

[476] Cooper GW, Hong AL. SMARCB1-Deficient Cancers: Novel Molecular Insights and Therapeutic Vulnerabilities. Cancers (Basel). 2022;14(15):3645. 35892904 10.3390/cancers14153645 PMC9332782

[477] Hong AL, Tseng YY, Wala JA, Kim WJ, Kynnap BD, Doshi MB, et al. Renal medullary carcinomas depend upon SMARCB1 loss and are sensitive to proteasome inhibition. Elife. 2019;8:e44161. 30860482 10.7554/eLife.44161 PMC6436895

[478] Zhang Z, Li J, Guo H, Wang F, Ma L, Du C, et al. BRM transcriptionally regulates miR-302a-3p to target SOCS5/STAT3 signaling axis to potentiate pancreatic cancer metastasis. Cancer Lett. 2019;449:215–25. 30790683 10.1016/j.canlet.2019.02.031

[479] Wanior M, Kramer A, Knapp S, Joerger AC. Exploiting vulnerabilities of SWI/SNF chromatin remodelling complexes for cancer therapy. Oncogene. 2021;40(21):3637–54. 33941852 10.1038/s41388-021-01781-x PMC8154588

[480] Yau EH, Kummetha IR, Lichinchi G, Tang R, Zhang Y, Rana TM. Genome-Wide CRISPR Screen for Essential Cell Growth Mediators in Mutant KRAS Colorectal Cancers. Cancer Res. 2017;77(22):6330–9. 28954733 10.1158/0008-5472.CAN-17-2043 PMC5690866

[481] Papillon JPN, Nakajima K, Adair CD, Hempel J, Jouk AO, Karki RG, et al. Discovery of Orally Active Inhibitors of Brahma Homolog (BRM)/SMARCA2 ATPase Activity for the Treatment of Brahma Related Gene 1 (BRG1)/SMARCA4-Mutant Cancers. J Med Chem. 2018;61(22):10155–72. 30339381 10.1021/acs.jmedchem.8b01318

[482] Vaswani RG, Huang DS, Anthony N, Xu L, Centore R, Schiller S, et al. Discovery of FHD-286, a First-in-Class, Orally Bioavailable, Allosteric Dual Inhibitor of the Brahma Homologue (BRM) and Brahma-Related Gene 1 (BRG1) ATPase Activity for the Treatment of SWItch/Sucrose Non-Fermentable (SWI/SNF) Dependent Cancers. J Med Chem. 2025;68(2):1772–92. 39801091 10.1021/acs.jmedchem.4c02535

[483] DiNardo CD, Fathi AT, Kishtagari A, Bhalla KN, Quintás-Cardama A, Reilly SA, et al. A Phase I Study of FHD-286, a Dual BRG1/BRM (SMARCA4/SMARCA2) Inhibitor, in Patients with Advanced Myeloid Malignancies. Clin Cancer Res. 2025;31(12):2327–38. 40238563 10.1158/1078-0432.CCR-24-3790

[484] Martin BJE, Ablondi EF, Goglia C, Mimoso CA, Espinel-Cabrera PR, Adelman K. Global identification of SWI/SNF targets reveals compensation by EP400. Cell. 2023;186(24):5290-307.e26. 37922899 10.1016/j.cell.2023.10.006 PMC11307202

[485] Lee JY, Brooks N, Antonakos B, Perria B, Langan C, Jones BD, et al. Abstract PR015: Discovery of LY4050784 (FHD-909), a selective BRM (SMARCA2) ATPase inhibitor for the treatment of BRG1(SMARCA4) mutant cancers. Molecular Cancer Therapeutics. 2024;23(6_Supplement):PR015-PR.

[486] Zhu J, Bai X, Xiong Y, Xie C, Chen Y, Sun H. Discovery of Novel Selective Inhibitors of SMARCA2 ATPase Domain by Virtual Screening and Biological Evaluation. ACS Med Chem Lett. 2025;16(10):2032–40. 41089483 10.1021/acsmedchemlett.5c00459 PMC12516363

[487] Cantley J, Ye X, Rousseau E, Januario T, Hamman BD, Rose CM, et al. Selective PROTAC-mediated degradation of SMARCA2 is efficacious in SMARCA4 mutant cancers. Nat Commun. 2022;13(1):6814. 36357397 10.1038/s41467-022-34562-5 PMC9649729

[488] Hulse M, Wang M, Xu C, Carter J, Agarwal A, Lu L, et al. PRT3789 Is a First-in-Human SMARCA2-Selective Degrader That Induces Synthetic Lethality in SMARCA4-Mutated Cancers. Cancer Res. 2026;86(1):213–35. 40991405 10.1158/0008-5472.CAN-25-1141

[489] Botta GP, Kato S, Patel H, Fanta P, Lee S, Okamura R, et al. SWI/SNF complex alterations as a biomarker of immunotherapy efficacy in pancreatic cancer. JCI Insight. 2021;6(18):e150453. 34375311 10.1172/jci.insight.150453 PMC8492298

[490] Liu XD, Kong W, Peterson CB, McGrail DJ, Hoang A, Zhang X, et al. PBRM1 loss defines a nonimmunogenic tumor phenotype associated with checkpoint inhibitor resistance in renal carcinoma. Nat Commun. 2020;11(1):2135. 32358509 10.1038/s41467-020-15959-6 PMC7195420

[491] Cui L, Liu R, Han S, Zhang C, Wang B, Ruan Y, et al. Targeting Arachidonic Acid Metabolism Enhances Immunotherapy Efficacy in ARID1A-Deficient Colorectal Cancer. Cancer Res. 2025;85(5):925–41. 39652583 10.1158/0008-5472.CAN-24-1611 PMC11873721

[492] Bagheri M, Mohamed GA, Mohamed Saleem MA, Ognjenovic NB, Lu H, Kolling FW, et al. Pharmacological induction of chromatin remodeling drives chemosensitization in triple-negative breast cancer. Cell Rep Med. 2024;5(4):101504. 38593809 10.1016/j.xcrm.2024.101504 PMC11031425

[493] Gao F, Li P, Kong X, Song T, Han Q, Zhou S. ARID1A-mutated cervical cancer depends on the activation of YAP signaling. Front Biosci (Landmark Ed). 2021;26(12):1411–21. 34994156 10.52586/5035

[494] Tolcher AW, Lakhani NJ, McKean M, Lingaraj T, Victor L, Sanchez-Martin M, et al. A phase 1, first-in-human study of IK-930, an oral TEAD inhibitor targeting the Hippo pathway in subjects with advanced solid tumors. Journal of Clinical Oncology. 2022;40(16_suppl):TPS3168-TPS.

[495] Yap TA, Kwiatkowski DJ, Dagogo-Jack I, Offin M, Zauderer MG, Kratzke R, et al. YAP/TEAD inhibitor VT3989 in solid tumors: a phase 1/2 trial. Nat Med. 2025;31(12):4281–90. 41111090 10.1038/s41591-025-04029-3

[496] Garralda E, Florescu M, Aerts JG, Yoshida T, Postel-Vinay S, Dagogo-Jack I, et al. 919O A first-in-human study of oral IAG933 in adult patients with advanced mesothelioma and other solid tumours. Ann Oncol. 2025;36:S562.

[497] Brien GL, Remillard D, Shi J, Hemming ML, Chabon J, Wynne K, et al. Targeted degradation of BRD9 reverses oncogenic gene expression in synovial sarcoma. Elife. 2018;7:e41305. 30431433 10.7554/eLife.41305 PMC6277197

[498] Vallurupalli M, Gorelov R, Garcia JS, Morgan EA, Ebert B, Tothova Z. Talazoparib treatment preferentially depletes cohesin-mutant clones in new *in vivo* models of cohesin-mutant myeloid diseases. Blood. 2019;134(Supplement_1):560.

[499] Ji M, Yu D, Liu X, Wang L, Zhang D, Yang Z, et al. Glutathione-dependent degradation of SMARCA2/4 for targeted lung cancer therapy with improved selectivity. Eur J Med Chem. 2024;277:116751. 39128328 10.1016/j.ejmech.2024.116751

[500] Byun WS, Zhuang Z, Hnatiuk AP, Jin C, Jiang Z, Baek K, et al. Discovery of BRD9 Molecular Glue Degraders That Spare Cardiomyocytes. J Am Chem Soc. 2025;147(39):35481–92. 40960846 10.1021/jacs.5c09857

[501] Baker Dockrey SA, Chandra P, Chen H, Chen S, Cheung TK, Cosino E, et al. Antibody-Mediated Delivery of BRM/BRG1 Protein Degraders Affords Strong Antitumor Efficacy in Multiple BRM-Dependent Non-Small Cell Lung Cancer Xenograft Models. J Med Chem. 2026;69(10):12583–618. 42144767 10.1021/acs.jmedchem.6c00592

[502] Helming KC, Wang X, Wilson BG, Vazquez F, Haswell JR, Manchester HE, et al. ARID1B is a specific vulnerability in ARID1A-mutant cancers. Nat Med. 2014;20(3):251–4. 24562383 10.1038/nm.3480 PMC3954704

[503] Megino-Luque C, Sisó P, Mota-Martorell N, Navaridas R, de la Rosa I, Urdanibia I, et al. ARID1A-deficient cells require HDAC6 for progression of endometrial carcinoma. Mol Oncol. 2022;16(11):2235–59. 35167193 10.1002/1878-0261.13193 PMC9168762

[504] Tsuda M, Fukuda A, Kawai M, Araki O, Seno H. The role of the SWI/SNF chromatin remodeling complex in pancreatic ductal adenocarcinoma. Cancer Sci. 2021;112(2):490–7. 33301642 10.1111/cas.14768 PMC7894000

[505] Shankari G, Prabhu D, Sureshan M, Jeyakanthan J, Rajamanikandan S. A Review of ARID1A's Role in Breast Cancer Progression: Context-Dependent Mechanisms and Therapeutic Implications. Cancers (Basel). 2025;18(1):142. 41514650 10.3390/cancers18010142 PMC12785034

[506] Zhang FL, Li DQ. Targeting Chromatin-Remodeling Factors in Cancer Cells: Promising Molecules in Cancer Therapy. Int J Mol Sci. 2022;23(21):12815. 36361605 10.3390/ijms232112815 PMC9655648

[507] Malone HA, Roberts CWM. Chromatin remodellers as therapeutic targets. Nat Rev Drug Discov. 2024;23(9):661–81. 39014081 10.1038/s41573-024-00978-5 PMC11534152

[508] Gourisankar S, Krokhotin A, Wenderski W, Crabtree GR. Context-specific functions of chromatin remodellers in development and disease. Nat Rev Genet. 2024;25(5):340–61. 38001317 10.1038/s41576-023-00666-x PMC11867214

[509] Zhang N, Coutinho LE, Pati D. PDS5A and PDS5B in Cohesin Function and Human Disease. Int J Mol Sci. 2021;22(11):5868. 34070827 10.3390/ijms22115868 PMC8198109

[510] Patnaik E, Madu C, Lu Y. Epigenetic Modulators as Therapeutic Agents in Cancer. Int J Mol Sci. 2023;24(19):14964. 37834411 10.3390/ijms241914964 PMC10573652

[511] Ren L, Yang Y, Li W, Yang H, Zhang Y, Ge B, et al. Recent advances in epigenetic anticancer therapeutics and future perspectives. Front Genet. 2022;13:1085391. 36685834 10.3389/fgene.2022.1085391 PMC9845602

[512] Neefjes J, Gurova K, Sarthy J, Szabo G, Henikoff S. Chromatin as an old and new anticancer target. Trends Cancer. 2024;10(8):696–707. 38825423 10.1016/j.trecan.2024.05.005 PMC11479676

[513] Chaudhri A, Lizee G, Hwu P, Rai K. Chromatin Remodelers Are Regulators of the Tumor Immune Microenvironment. Cancer Res. 2024;84(7):965–76. 38266066 10.1158/0008-5472.CAN-23-2244

[514] Ghorbian S. Cancer cell plasticity and therapeutic resistance: mechanisms, crosstalk, and translational perspectives. Hereditas. 2025;162(1):188. 41013839 10.1186/s41065-025-00564-8 PMC12465352

[515] Zhong L, Li Y, Xiong L, Wang W, Wu M, Yuan T, et al. Small molecules in targeted cancer therapy: advances, challenges, and future perspectives. Signal Transduct Target Ther. 2021;6(1):201. 34054126 10.1038/s41392-021-00572-w PMC8165101

[516] Liu B, Zhou H, Tan L, Siu KTH, Guan XY. Exploring treatment options in cancer: Tumor treatment strategies. Signal Transduct Target Ther. 2024;9(1):175. 39013849 10.1038/s41392-024-01856-7 PMC11252281

[517] Gou X, He S, Wang B, Zhang L, Cheng Y, Gao X. Integrating PROTAC-based targeted protein degradation with nanodelivery systems to overcome cancer therapeutic resistance. Adv Drug Deliv Rev. 2026;229:115755. 41386500 10.1016/j.addr.2025.115755

[518] Montano-Samaniego M, Bravo-Estupinan DM, Mendez-Guerrero O, Alarcon-Hernandez E, Ibanez-Hernandez M. Strategies for Targeting Gene Therapy in Cancer Cells With Tumor-Specific Promoters. Front Oncol. 2020;10:605380. 33381459 10.3389/fonc.2020.605380 PMC7768042

[519] Dragovich PS. Degrader-antibody conjugates. Chem Soc Rev. 2022;51(10):3886–97. 35506708 10.1039/d2cs00141a

[520] Tsai PF, Dell'Orso S, Rodriguez J, Vivanco KO, Ko KD, Jiang K, et al. A Muscle-Specific Enhancer RNA Mediates Cohesin Recruitment and Regulates Transcription In trans. Mol Cell. 2018;71(1):129–41 e8. 10.1016/j.molcel.2018.06.008 PMC6082425 29979962

[521] Horsfield JA. Full circle: a brief history of cohesin and the regulation of gene expression. FEBS J. 2023;290(7):1670–87. 35048511 10.1111/febs.16362

[522] Grubert F, Srivas R, Spacek DV, Kasowski M, Ruiz-Velasco M, Sinnott-Armstrong N, et al. Landscape of cohesin-mediated chromatin loops in the human genome. Nature. 2020;583(7818):737–43. 32728247 10.1038/s41586-020-2151-x PMC7410831

[523] Swett AD, Tothova Z. Cohesin mutations and chromatin changes in cancer. Int J Cancer. 2026;158(2):368–81. 40063003 10.1002/ijc.35378 PMC12576943

[524] Burke MR, Smith AR, Zheng G. Overcoming cancer drug resistance utilizing PROTAC technology. Front Cell Dev Biol. 2022;10:872729. 35547806 10.3389/fcell.2022.872729 PMC9083012

[525] Kargbo RB. SMARCA2/4 PROTAC for targeted protein degradation and cancer therapy. ACS Med Chem Lett. 2020;11(10):1797–8. 33062156 10.1021/acsmedchemlett.0c00347 PMC7549100

[526] Zoppi V, Hughes SJ, Maniaci C, Testa A, Gmaschitz T, Wieshofer C, et al. Iterative design and optimization of initially inactive proteolysis targeting chimeras (PROTACs) identify VZ185 as a potent, fast, and selective von Hippel-Lindau (VHL) based dual degrader probe of BRD9 and BRD7. J Med Chem. 2019;62(2):699–726. 30540463 10.1021/acs.jmedchem.8b01413 PMC6348446

[527] Peng X, Hu Z, Zeng L, Zhang M, Xu C, Lu B, et al. Overview of epigenetic degraders based on PROTAC, molecular glue, and hydrophobic tagging technologies. Acta Pharm Sin B. 2024;14(2):533–78. 38322348 10.1016/j.apsb.2023.09.003 PMC10840439

[528] Schreiber SL. The Rise of Molecular Glues. Cell. 2021;184(1):3–9. 33417864 10.1016/j.cell.2020.12.020

[529] Zhang Y, Li L, Xu J, Che C, Jia J, Lu H, et al. A Novel Paradigm for Targeting Challenging Targets: Advancing Technologies and Future Directions of Molecular Glue Degraders. Molecules. 2026;31(3);459. 10.3390/molecules31030459 PMC12898511 41683435

[530] Liu Y, Ullah I, Yuan Y, Wang J. Harnessing targeted protein degradation to potentiate cancer immunotherapy: from molecular mechanisms to delivery strategies. Adv Drug Deliv Rev. 2026;230:115776. 41525883 10.1016/j.addr.2026.115776

[531] Zhang Y, Gu W, Chen W, Zhu J, Fan L, Zhang L, et al. A Dual-Targeted Molecule for Disease-Activatable Proteolysis Targeting Chimeras and Targeted Radionuclide Therapy of Cancer. J Am Chem Soc. 2025;147(9):7897–907. 39989465 10.1021/jacs.4c18398

[532] Sarott RC, Gourisankar S, Karim B, Nettles S, Yang H, Dwyer BG, et al. Relocalizing transcriptional kinases to activate apoptosis. Science. 2024;386(6717):eadl5361. 39361741 10.1126/science.adl5361 PMC11629774

[533] Gourisankar S, Krokhotin A, Ji W, Liu X, Chang CY, Kim SH, et al. Rewiring cancer drivers to activate apoptosis. Nature. 2023;620(7973):417–25. 37495688 10.1038/s41586-023-06348-2 PMC10749586

[534] Huang K. Chemical inducers of proximity: precision tools for apoptosis in transcriptional regulation. Signal Transduct Target Ther. 2024;9(1):364. 39702508 10.1038/s41392-024-02089-4 PMC11659315

[535] Li Q, Qian W, Zhang Y, Hu L, Chen S, Xia Y. A new wave of innovations within the DNA damage response. Signal Transduct Target Ther. 2023;8(1):338. 37679326 10.1038/s41392-023-01548-8 PMC10485079

[536] Buckley-Benbow L, Agnarelli A, Bellelli R. “Where is my gap”: mechanisms underpinning PARP inhibitor sensitivity in cancer. Biochem Soc Trans. 2025;53(1):225–34. 39927794 10.1042/BST20241633 PMC12203949

[537] Hein KZ, Stephen B, Fu S. Therapeutic Role of Synthetic Lethality in ARID1A-Deficient Malignancies. J Immunother Precis Oncol. 2024;7(1):41–52. 38327752 10.36401/JIPO-22-37 PMC10846636

[538] Bhamidipati D, Haro-Silerio JI, Yap TA, Ngoi N. PARP inhibitors: enhancing efficacy through rational combinations. Br J Cancer. 2023;129(6):904–16. 37430137 10.1038/s41416-023-02326-7 PMC10491787

[539] Lee SG, Kim J, Jeong E, Myung K. DNA damage response inhibitors in cancer therapy: Mechanisms, clinical development, and combination strategies. DNA Repair (Amst). 2025;153:103887. 40865183 10.1016/j.dnarep.2025.103887

[540] Mumbach MR, Rubin AJ, Flynn RA, Dai C, Khavari PA, Greenleaf WJ, et al. HiChIP: efficient and sensitive analysis of protein-directed genome architecture. Nat Methods. 2016;13(11):919–22. 27643841 10.1038/nmeth.3999 PMC5501173

[541] Hansen KL, Adachi AS, Braccioli L, Kadvani S, Boileau RM, Martinovic M, et al. Synergy between regulatory elements can render cohesin dispensable for distal enhancer function. Science. 2026;391(6785):eadt4221. 41308125 10.1126/science.adt4221 PMC13446962

[542] Nora EP, Caccianini L, Fudenberg G, So K, Kameswaran V, Nagle A, et al. Molecular basis of CTCF binding polarity in genome folding. Nat Commun. 2020;11(1):5612. 33154377 10.1038/s41467-020-19283-x PMC7645679

[543] Tufail M, Jiang CH, Li N. Immune evasion in cancer: mechanisms and cutting-edge therapeutic approaches. Signal Transduct Target Ther. 2025;10(1):227. 40739089 10.1038/s41392-025-02280-1 PMC12311175

[544] Motz GT, Coukos G. Deciphering and reversing tumor immune suppression. Immunity. 2013;39(1):61–73. 23890064 10.1016/j.immuni.2013.07.005 PMC3782392

[545] Vandivier RW, Ghosh M. Understanding the Relevance of the Mouse Cigarette Smoke Model of COPD: Peering through the Smoke. Am J Respir Cell Mol Biol. 2017;57(1):3–4. 28665224 10.1165/rcmb.2017-0110ED PMC5516286

[546] Sun G, Li Z, Rong D, Zhang H, Shi X, Yang W, et al. Single-cell RNA sequencing in cancer: applications, advances, and emerging challenges. Mol Ther Oncolytics. 2021;21:183–206. 34027052 10.1016/j.omto.2021.04.001 PMC8131398

[547] Ianevski A, Nader K, Driva K, Senkowski W, Bulanova D, Moyano-Galceran L, et al. Single-cell transcriptomes identify patient-tailored therapies for selective co-inhibition of cancer clones. Nat Commun. 2024;15(1):8579. 39362905 10.1038/s41467-024-52980-5 PMC11450203

[548] Zhao B, Lin J, Rong L, Wu S, Deng Z, Fatkhutdinov N, et al. ARID1A promotes genomic stability through protecting telomere cohesion. Nat Commun. 2019;10(1):4067. 31492885 10.1038/s41467-019-12037-4 PMC6731242

